# Transcriptome analysis reveals gene expression changes of pigs infected with non-lethal African swine fever virus

**DOI:** 10.1590/1678-4685-GMB-2023-0037

**Published:** 2023-10-13

**Authors:** Wen Feng, Lei Zhou, Heng Du, Edward Okoth, Raphael Mrode, Wenjiao Jin, Zhengzheng Hu, Jian-Feng Liu

**Affiliations:** 1 State Key Laboratory of Animal Biotech Breeding, Beijing, China.; 2 Ministry of Agriculture, Key Laboratory of Animal Genetics, Breeding and Reproduction, Beijing, China.; 3 China Agricultural University, Frontiers Science Center for Molecular Design Breeding (MOE), Beijing, China.; 4 China Agricultural University, College of Animal Science and Technology, Beijing, China.; 5 Yulin University, College of Life Sciences, Shaanxi, China.; 6 International Livestock Research Institute, Nairobi, Kenya.

**Keywords:** ASFV, RNA-Sequencing, transcriptome, gene expression, non-lethal

## Abstract

African swine fever (ASF) is an important viral disease of swine caused by the African swine fever virus (ASFV), which threatens swine production profoundly. To better understand the gene expression changes when pig infected with ASFV, RNA sequencing was performed to characterize differentially expressed genes (DEGs) of six tissues from Kenya domestic pigs and Landrace × Yorkshire (L/Y) pigs infected with ASFV Kenya1033 in vivo. As results, a total of 209, 522, 34, 505, 634 and 138 DEGs (q-value < 0.05 and |Log2foldchange| values >2) were detected in the kidney, liver, mesenteric lymph node, peripheral blood mononuclear cell, submandibular lymph node and spleen, respectively. The expression profiles of DEGs shared in the multiple tissues illustrated variation in regulation function in the different tissues. Functional annotation analysis and interaction of proteins encoded by DEGs revealed that genes including *IFIT1, IFITM1, MX1, OASL, ISG15, SAMHD1, IFINA1, S100A12* and *S100A8* enriched in the immune and antivirus pathways were significantly changed when the hosts were infected with ASFV. The genes mentioned could play crucial roles in the process of the reaction to non-lethal ASF infection, which may will help to improve the ASF tolerance in the pig population through molecular breeding strategies.

## Introduction

African swine fever (ASF) is an acute, febrile, and highly infectious disease caused by the African swine fever virus (ASFV) in pigs. This transboundary animal disease can spread by live or dead pigs, domestic or wild, and pork products. Due to the high environmental resistance of ASFV, transmission can also occur via contaminated feed and fomites (non-living objects) such as shoes, clothes, vehicles, knives, equipment, etc. Currently, it is one of the most threatening diseases for the global swine industry. ASF outbreaks have been reported in many countries in Africa and Europe, South America, and Asia. In 2018, the outbreak of ASF in China, which is the biggest pork producer in the world, has caused lots of economic losses.

ASFV is an enveloped virus that contains a length of 170-193 kb double-stranded DNA genome. More than 50 different proteins encoded by 150-167 genes participate in genome replication and viral infection (Dixon *et al.*, 2013). Many types of genotypes were detected in the globe ASFVs. Genotypes IX and X are most populated in eastern Africa, and the genotype I ASFV was detected in Southeast Africa, Russia, eastern Europe, and East Asia (King *et al.*, 2011; Bishop *et al.*, 2015; Njau *et al.*, 2021; Peter *et al.*, 2021). As a highly conserved gene, the ASFV P72 gene is usually used in ASFV identification in vitro (Bastos *et al.*, 2003; King *et al.*, 2003).

ASFV encodes many proteins that suppress the innate immune response, such as the main antiviral response type I interferon (IFN). The ASFV A528R protein inhibits the induction of the NF-κB, the type I IFN-induced signaling pathway, and the effect of type I and type II interferon stimulation in the IFN response (Correia *et al.*, 2013). Besides that, ASFV MGF360-12L protein was found significantly to inhibit the mRNA transcription and promoter activity of IFN-β and NF-κB (Zhuo *et al.*, 2021). These findings may reveal reasons for the ability of ASFV to escape the innate immune response of the host. 

RNA-Seq technology has been applied to the transcriptome study of pigs infected with both a highly virulent (Georgia 2007 strain, GRG) and low (OURT) virulent ASFV (Jaing *et al.*, 2017). RNA-Seq detected the expression of ASFV genes from the whole blood of the GRG, but not the OURT pigs, consistent with the pathotypes of these strains and the replication of GRG in circulating monocytes. This study mainly focused on the ASFV genes and upregulated pig differential expressed genes after pigs were infected with ASFV.

To detect the gene expression changes of pigs infected with at non-lethal African swine fever virus, two pig populations, the Kenya domestic pigs and the Landrace × Yorkshire pigs, infected with low dose of ASFV Kenya1033 in vivo were conducted. ASFV Kenya1033 belongs to genotype IX and was obtained from a domestic pig in western Kenya (Onzere *et al.*, 2018). RNA-Sequencing on the five tissues and peripheral blood mononuclear cell (PBMC) of these two pig populations were used to characterize the changes of genes expression between ASFV infected pigs and control pigs. These attempts were made to identify the gene expression changes of pigs infected with at non-lethal ASFV.

## Material and Methods

### Preparation of samples

Six Yorkshire × Landrace crossed (L/Y) pigs and six Kenyan domestic pigs ages at 9-10.5 months used in this study were from a pig farm in Homabay, Kenya. These 12 pigs were used for infection trial *in vivo*. Before infection, all pigs were acclimatized to animal rooms for one month. Four L/Y pigs and four Kenyan domestic pigs in one Animal Biosafety Laboratory Level 3 (ABSL3) were prepared for inoculating with the ASFV, while two L/Y and two Kenyan domestic pigs in another ABSL3 were kept healthy as control pigs. Before virus infection, PCR was used to screen for the conservative sequence of the P72 gene of ASFV to confirm whether the pigs were clean from prior exposure to ASFV. The primer set (PPA1, 5′-AGTTATGGGAAACCCGACCC-3′; PPA2, 5′-CCCTGAATCGGAGCATCCT-3′), with the product length of 257 bp and PCR system was followed by a previous study to characterize ASFV (Aguero *et al.*, 2003). The PCR results showed that all 12 pigs were ASFV free.

### ASFV infection

Animal work of virus infection and related samples collection and treatments were approved by the Institutional Animal Care and Use Committee (IACUC) of International Livestock Research Institute (ILRI) (ref no. IACUC-RC2018-17). All procedures were conducted according to the ILRI IACUC protocol 11.

Four Kenya domestic pigs and four L/Y pigs were infected with ASFV Kenya 1033 genotype IX by intramuscular injection. The titer was calculated using the Spearman and Kärber algorithm (Hierholzer and Killington, 1996) and are expressed as HAD_50_/mL (Enjuanes *et al.*, 1976) in this study, which is 10^2.7^/ml. For the ASFV-inoculated pigs, whole blood was collected before inoculation then on day 2, 5, 7, 9, 12, 14, 16, 19, 21, and 23, for peripheral blood mononuclear cell (PBMC) isolation. All 12 pigs used in the trial were humanly euthanased using gunshots (0.22 caliber, head) on Day 23 post infection. Five tissues including kidney, liver, mesenteric lymph node (MSLN), submandibular lymph node (SMLN), and spleen were collected from these 12 pigs immediately after slaughter and stored in liquid nitrogen for DNA and RNA extraction.

### RNA sequencing

Total RNAs were isolated from liver, kidney, MSLN, PBMC, SMLN and spleen according to the standard protocols of the Trizol method (Invitrogen, Carlsbad, CA, United States). RNA degradation and contamination were monitored on 1% agarose gels. The total RNA concentration was measured using Qubit RNA Assay Kit in a Qubit 2.0 Fluorometer (Life Technologies, Carlsbad, CA, United States). RNA samples that met the criteria of RNA integrity number (RIN) value ≧7.0 and a total RNA amount of ≧5 μg, were included for RNA sequencing. RNA sequencing libraries were constructed using the Kapa RiboErase (Roche, Basel, Switzerland), with 3 μg rRNA-depleted RNA, according to the manufacturer. Then, libraries were sequenced using the Illumina NovaSeq 6000 S4 platform according to the manufacturer, with a data size per sample of a minimum of 5G clean reads (corresponding to 150 bp paired-end reads). The sequenced DNA-Seq and RNA-Seq raw data are available from NCBI Sequences Read Archive with the BioProject number PRJNA691462.

### Reads alignment to the reference genome Sus Scrofa 11.1

The RNA-Seq raw data were trimmed based on the quality control for downstream analyses by following steps: Firstly, BBmap (Version v0.38) automatically detected the adapter sequence of reads and removed those reads containing Illumina adapters (Brian, 2014). The Q20, Q30, and GC content of the clean data were also calculated by FastQC for quality control and filtering (Andrews, 2010). Secondly, the resulting sequences were mapped to the reference genome (Sus scrofa 11.1) by HISAT2 (Dobin *et al.*, 2013). NCBI Sus scrofa11.1 (https://www.ncbi.nlm.nih.gov/assembly/GCF_000003025.6) annotation was used as the transcript model reference for the alignment, as well as for all protein-coding genes and isoform expression-level quantifications. Finally, FeatureCounts (from subread v2.0.1) was used to calculate the number of the read counts (Liao *et al.*, 2014).

### Differentially expressed genes analysis

In this study, two population were merged into one for differential expressed genes (DEGs) detection. DEGs were screened between the 8 infectious pigs and 4 control pigs. The false discovery rate (FDR) was calculated by the DESeq2 package of R v4.0.2, termed as q-values for differential expression analysis. Genes met the criteria q < 0.05 and |log2 FoldChange| >2 were considered DEGs. The functional GO, KEGG and Reactome pathways enrichment of DEGs were clustered using KOBAS3.0 (http://kobas.cbi.pku.edu.cn/kobas3/genelist/). The pathways with q < 0.05 were considered significant. 

### Protein-protein interaction (PPI) network establishment

Protein-protein interaction (PPI) networks were constructed using the Search Tool for the Retrieval of Interacting Genes (STRING) online database (Szklarczyk *et al.*, 2019), with an interaction score >0.9 set as the cut-off value. 

### The expression of fluorescent protein in 3D4/21 cells after been transfected plasmid

To investigate the function of gene *IFIT1* on ASFV, the expression of fluorescent protein in porcine alveolar macrophages 3D4/21 cells transfected with pEGFP-C1-IFIT1 were detected.

The porcine alveolar macrophages 3D4/21 used in this study were purchased from the American Type Culture Collection (ATCC CRL-2843). The whole experiment was completed in the Institute of Military Veterinary Medicine, Academy of Military Medical Science. The ASFV strain SY18 of genotype II (GenBank accession number MH766894) used in this study was supplied by the Institute of Military Veterinary Medicine, Academy of Military Medical Science. The culture and viral titer determination of ASFV SY18 were described in the previous paper by Fan et al (Fan *et al.*, 2020). 

The pEGFP-C1-IFIT1 and bland control pEGFP-C1 vectors were constructed by TsingKe Biological Technology, Beijing, China. Primary alveolar macrophages 3D4/21 were seeded in 6-well plates at a concentration of 2 × 10^6^/mL and incubated at 37 ℃ with 5% CO_2_ in RPMI 1640 maintenance medium. When the cells density was around 70~90%, the pEGFP-C1-IFIT1 and blank control pEGFP-C1 vectors were transfected into cells according to the lipo3000 instructions, and incubated at 37 ℃ with 5% CO_2_ for 24 hours. Then the cells were infected with ASFV strain SY18 (MOI =5) and were digested and harvested by centrifugation at 48 h post-infection. The cells were observed using a confocal laser scanning fluorescence microscope (Olympus LSCMFV500).

Animals work of virus infection and related samples collection and treatments were approved by the Institutional Animal Care and Use Committee (IACUC) of International Livestock Research Institute (ILRI) (ref no. IACUC-RC2018-17).

## Results

### Clinical presentation and gross pathology of ASFV infection

**Figure 1 -:**
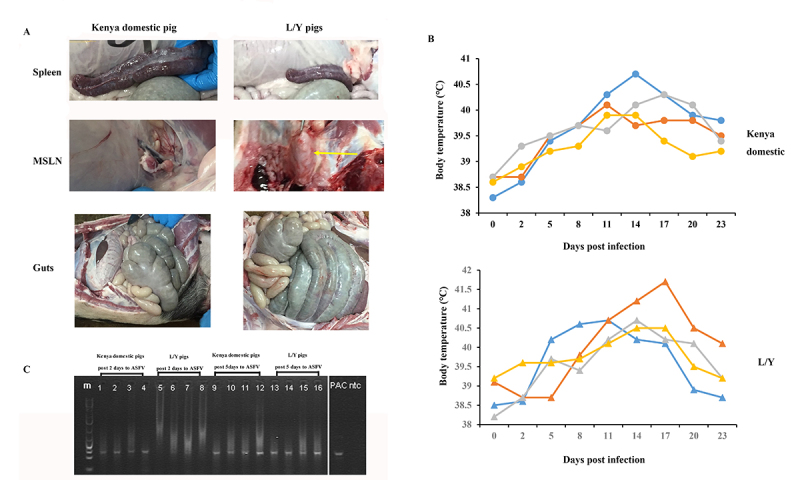
(A) Clinical presentation of tissues after ASFV infection. MSLN is short for mesenteric lymph node. (B) The body temperatures for each individual in Kenya domestic and L/Y pigs. ● represent Kenya domestic pigs and ▲ represent L/Y pigs. **(C)** ASFV detection by PCR in blood. M represents Marker; 1-4 represent Kenya domestic pigs after ASFV infection on Day 2; 5-8 represent L/Y pigs after ASFV infection on Day 2; 9-12 represent Kenya domestic pigs after ASFV infection on Day 5; 13-16 represent L/Y pigs after ASFV infection on Day 5. PAC: ASFV positive control; NTC: ASFV negative control. The white line represents that PAC and NTC were from different parts of the same gel and the unrelated lanes to this study were removed **(** Figure S1).

For both Kenya domestic and Landrace × Yorkshire (L/Y) pigs after ASFV infection, only mild or non-specific clinical sign was observed, including less appetite and skin lesions. Lesions occurred most on the legs and heads. At the Day 23 after ASFV infection, all ASFV infectious pigs showed splenomegaly and emorrhagic spleen. A multifocal hemorrhagic lymphadenitis can also be observed with multiple lymph nodes in all body areas showing the hemorrhages and the “marble” appearance. Multifocal pneumonia was also observed with patches of consolidation and dark color in the liver. Hemorrhages and enlaergement were also seen in the gastrointestinal tract. Despite this, all infected pigs were survived (Figure 1A). The body temperatures showed similar trends between Kenya domestic pigs and L/Y pigs. At days 14 post infection, body temperature of pigs reached peaks. At days 23 post infection, their body temperature gradually returned to normal body temperature (Figure 1B, which was cropped from Figure S1). At the Day 5 after ASFV infection, gene *P72* of ASFV were detected from PBMCs of all infected pigs, which means pigs were successfully infected with ASFV (Figure 1C).

### Sequencing and gene expression statistics

Altogether 76 samples of six tissues from six Kenya domestic pigs, six L/Y pigs were subjected to RNA sequencing. Two samples out of 76 had sequencing failure and thus were excluded in the analysis. The numbers of sequencing reads obtained from the 74 samples are summarized in Table S1. An average of 45.63 M reads were obtained from the L/Y pig samples, and 45.15 M reads from the Kenyan domestic pig samples. The average Q30 percentages of two breeds were 91.72% and 90.61%, respectively. All mapped reads were used to estimate gene expression.

### The whole genome-wide transcriptional changes of ASFV infected pigs

There was no significant difference between ASFV infected Kenya domestic pigs and L/Y pigs (Supplementary Data S1), and for this reason the two populations were considered as a single study population for purposes of screening for the differentially expressed genes (DEGs). 

The DEGs identified in each tissue using Q value and fold change values criteria is described in Table 1 and Table S2. A total of 209, 522, 34, 505, 634 and 138 DEGs that met the criteria of Q value < 0.05 and |Log2foldchange| values >2 was identified in the kidney, liver, mesenteric lymph node (MSLN), Peripheral blood mononuclear cell (PBMC), submandibular lymph node (SMLN) and spleen, respectively, when comparison was made between ASFV infected pigs and control pigs. The kidney, liver, PBMC, and SMLN, had more upregulated genes than the downregulated genes. In contrast, more downregulated genes than the upregulated genes were observed in the MSLN and spleen. The DEG results indicated that SMLN respond more strongly to infection with ASFV compared to kidney, liver, MSLN, PBMC and spleen. 

**Table 1 -:** Numbers of differentially expressed genes identified in different tissues in the comparison of ASFV infected and control pigs.

Criteria	Expression Models	Kidney	Liver	MSLN	PBMC	SMLN	Spleen
q <0.05	Total	3759	3183	79	1845	805	1533
	up	1589	1636	21	1153	595	440
	down	2170	1547	58	692	210	1093
q <0.05 |Log2 Fold change| >2	Total	209	522	34	505	634	138
	Up	162	420	11	322	553	43
	Down	47	102	23	183	81	95
q <0.05 |Log2 Fold change| >5	Total	2	18	3	16	118	1
	up	1	16	3	4	118	1
	down	1	2	0	12	0	0

Note: Up represents the upregulated genes comparing the ASFV infected pigs with control pigs; Down represents the downregulated genes comparing the ASFV infected pigs with control pigs.

**Figure 2 -:**
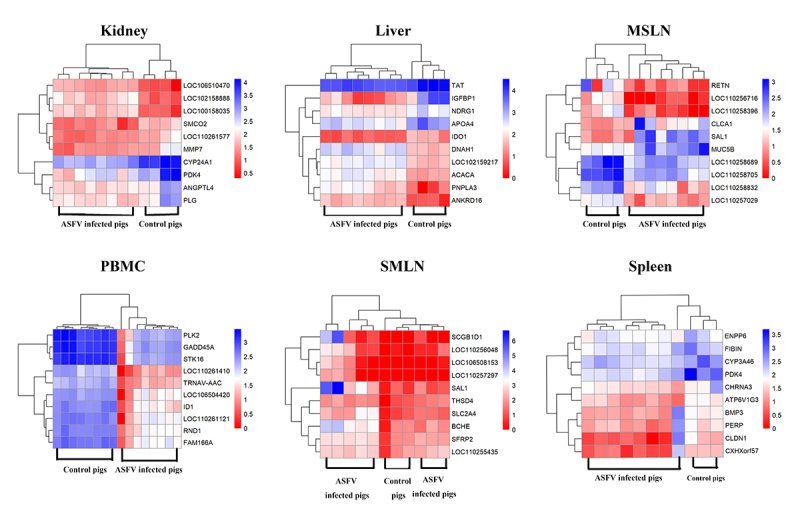
Heatmap of the top 10 DEGs of different tissues. The reads counts were transformed by log (10+1) (rows) are clustered using hierarchical clustering. MSLN is short for mesenteric lymph node; PBMC is short for peripheral blood mononuclear cell; SMLN is short for submandibular lymph node. Due to RNA sequencing failure, only 11 samples of SMLN and Spleen were used in this study. Red color means up-regulation, blue color means down-regulation. The darker the color, the higher the expression.

The expression profiles of the top 10 DEGs in these six tissues are shown in the Figure 2. When pigs were infected with the ASFV, 7 of the top 10 DEGs were downregulated in the kidney and MSLN. In the liver, the number of upregulated and downregulated DEGs were equal. All the top 10 DEGs in the spleen were downregulated, while all the DEGs were upregulated in the PBMC and SMLN. Among these DEGs, *SCGB1D1, LOC106508153, LOC110257297, SAL1, LOC110256048, BCHE,* and *SLC2A4* were expressed at very high levels (ranging from 6.35 to 27.05-fold when compared), and more interestingly, these DEGs were all found in the SMLN. Among the top 10 DEGs in each tissue, many known antiviral genes were identified, such as I*D1, MMP7, RETN, GADD45A, ATP6V1G3, MUC5B* and *SCGB1D1.*

### The key DEGs in different pig tissues in response to ASFV infection

DEGs shared in the six tissues are described in the Table 2. No DEG was shared in all the six tissues. Only one gene, *FARP2* (FERM, ARH/RhoGEF and Pleckstrin Domain Protein 2), was shared in four tissues that included liver, PBMC, SMLN and spleen. In the PBMC, the expression of *FARP2* decreased with ASFV infection compared with the control pigs. In the meanwhile, its expression increased in the liver, SMLN and spleen. Gene *FARP2* is involved in the Rho guanyl-nucleotide exchange factor activity and in the development and progression of cancer (Radu *et al.*, 2014). Altogether 10 DEGs were shared in three different tissues. Among these genes, *IL17B, LOC106510256, SAL1, AATK* and *TAT* showed same expression trends in different tissues (Table 3). Whilst, the gene expression trends of *SLC22A3, SFRP2, NMNAT2,* and *S100A12* varied in different tissues when pigs ASFV infected.

**Table 2 -:** The DEGs shared in different tissues.

Shared tissues	Number of genes	Genes
Liver PBMC SMLN Spleen	1	*FARP2*
Kidney Liver PBMC	1	*LOC100515788*
Kidney Liver SMLN	2	*SLC22A3 IL17B*
Kidney Liver Spleen	1	*LOC106510256*
Kidney SMLN Spleen	1	*SFRP2*
Liver MSLN SMLN	1	*SAL1*
Liver PBMC SMLN	1	*AATK*
Liver PBMC Spleen	3	*NMNAT2 TAT S100A12*
Kidney Liver	31	*LOC100621797 LOC110260835 RNASE6 MTHFR TAS2R39 LOC100515508 ENOSF1 VTCN1 PAN2 LOC102162170 LOC110256117 SSC5D NELFCD DNMT3A UNKL LOC110260623 LOC106509613 LOC100525821 MUC6 MYLK LOC102157481 LRRC74B LOC100158035 APOA4 FAM57B FAM209B LOC110261349 IFITM1 ADARB2 LOC100512969 LOC102159152*
Kidney PBMC	8	*LOC110259044 SLCO4A1 TELO2 CAMK1D LOC110260427 UBXN6 NCALD ING1*
Kidney SMLN	7	*LOC106506037 IQANK1 DPT LOC110256824 TIAM2 LOC110255698 GLI2*
Kidney Spleen	6	*LOC110260090 LOC106504762 FRMD4A PDK4 LOC100739719 LPL*
Liver MSLN	2	*LOC110257759 LOC100516039*
Liver PBMC	16	*DUSP22 LOC110258800 LOC106506012 LOC110256151 ITPR1 LOC110255523 LOC106504547 SFXN1 TRNAE-UUC SLC4A9 GSTM3 ARG2 DNAH8 LOC110261018 LOC102166913 HOPX*
Liver SMLN	29	*CBR2 PCSK1 RAB40B SUSD2 GHR CYP4F55 THRB PIGR LOC106507937 LOC110255743 PAQR4 LOC106505335 LOC102165183 LOC100514340 LOC102159217 LOC106508570 SNX7 NCKAP5 RNF152 KCNJ12 LOC100525628 LOC110260108 LOC100038328 LOC110257243 LOC110260785 LOC100524382 LOC106505659 PRDM7 ABLIM1*
Liver Spleen	5	*BMP3 NR1H3 LOC102159510 LOC100156325 LOC110261066*
MSLN PBMC	2	*RETN CEBPE*
MSLN SMLN	8	*CSPG4 LOC110258825 PHEROC LOC110258831 SCGB1D1 MX1 LOC110258832 LOC110258673*
MSLN Spleen	1	*CLCF1*
PBMC SMLN	14	*FAM46B GFPT2 NPTX2 LOC106504420 LOC110261655 SASH1 LOC110255861 ID4 LOC100154873 EBF2 LOC110260253 ITGA7 DPY19L1 RETREG1*
PBMC Spleen	2	*SLFNL1 NCOA2*
SMLN Spleen	7	*KRT8 EGFLAM PDE1A EPHX1 KRT7 MAP1B MTMR3*

**Table 3 -:** The expression profiles of DEGs shared in the four or three tissues.

	*FARP2*	*LOC100515788*	*SLC22A3*	*IL17B*	*LOC106510256*	*SFRP2*	*SAL1*	*AATK*	*NMNAT2*	*TAT*	*S100A12*
Kidney		-	-	+	+	-					
Liver	+	-	+	+	+		+	+	+	-	-
MSLN							+				
PBMC	-	-						+	-	-	+
SMLN	+		+	+		+	+	+			
Spleen	+				+	-			-	-	-

Note: “+” means this gene was upregulated in this tissue comparing the ASFV infected pigs with control pigs; “-” “+” means this gene was downregulated in this tissue comparing the ASFV infected pigs with control pigs; Blank space means this gene was not significant DEG in this tissue comparing the ASFV infected pigs with control pigs.

Kidney had 31, 8, 7 and 6 DEGs shared with liver, PBMC, SMLN and spleen, respectively. Liver had 2, 16, 29, and 5 DEGs shared with MSLN, PBMC, SMLN and spleen, respectively. MSLN had 2, 8 and 1 shared DEGs with PBMC, SMLN and spleen, respectively. PBMC had 14 and 2 DGESs shared with SMLN and spleen. There were 7 DEGs shared between SMLN and spleen. Among these genes, *MX1* and *IFITM1* were involved in defense response to virus. MX1 was downregulated and shared in both MSLN and SMLN. At the same time, *IFITM1* shared in the kidney and liver was also downregulated in both tissues. 

### Functional annotation of DEGs involved in ASFV

The KOBAS3.0 analysis of DEGs (Q < 0.05 and |Log2foldchange| > 2) in different tissues for comparison of the ASFV infected pigs and control pigs was carried out for the functional clusters. The GO analysis revealed 37, 144, 96, 224, 304, and 96 pathways in the kidney, liver, MSLN, PBMC, MSLN and spleen, respectively (Table S3-S8). Among these, three GO terms were related to virus pathways, including defense response to virus (GO:0051607), modulation by virus of host process (GO:0019048), negative regulation of viral genome replication (GO:0045071), and response to virus (GO:0009615). Gene *OASL* and *MX1* play important roles in these pathways related to the virus. Besides, these two genes were also enriched into the type I Interferon Signaling Pathway (GO:0060337) and response to type I Interferon (GO:0034340), which participate the regulation of pig when the host infected with ASFV (Bowie and Unterholzner, 2008). Total of 34 DEGs were enriched into 3 GO terms related to immune response and inflammatory response, including inflammatory response (GO:0006954), innate immune response (GO:0045087), and positive regulation of inflammatory response (GO:0050729). In the PBMC, three NF-κB related pathways were clustered, including the negative regulation of NF-κB transcription factor activity (GO:0032088), positive regulation of NF-κB transcription factor activity (GO:0051092), and positive regulation of NIK/ NF-κB signaling (GO:1901224). Gene *S100A12* was found involved in these immune and NF-Κb related pathways. 

**Figure 3 -:**
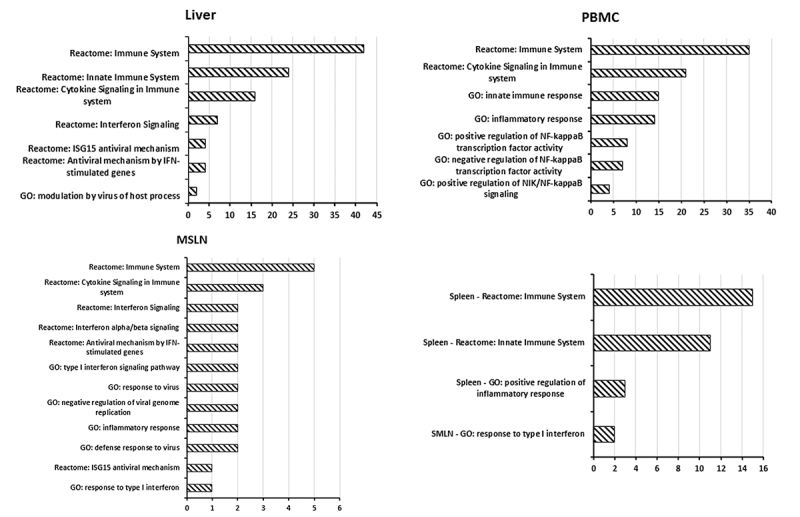
Immune and antivirus related GO and Reactome pathways enriched by KOBAS3.0. No significant immune or antivirus pathways were found in the kidney. Only one and three immune and antivirus related GO pathways were found in the SMLN and spleen, thus, pathways enriched in these two tissues were described together (right bottom). MSLN is short for mesenteric lymph node; PBMC is short for peripheral blood mononuclear cell; SMLN is short for submandibular lymph node.

A total of 3, 22, 4, 23, 37 and 12 KEGG (Kanehisa and Goto, 2000; Kanehisa *et al.*, 2021) pathways were enriched in the kidney, liver, MSLN, PBMC, MSLN and spleen, respectively (Table S3-S8). But no immune or antivirus KEGG pathways using our DEGs were clustered, thus, Reactome analysis was performed. DEGs from the kidney, liver, MSLN, PBMC, MSLN and spleen were enriched into 7, 52, 33, 53, 94, and 23 Reactome pathways, respectively (Table S3-S8). Among these pathways, two were related to the antivirus, which were antiviral mechanism by IFN-stimulated genes (R-HSA-1169410) and ISG15 antiviral mechanism (R-HSA-1169408). Pathways of cytokine signaling in immune system pathway (R-HSA-1280215), immune system pathway (R-HSA-168256), innate immune system pathway (R-HSA-168249), interferon alpha/beta signaling pathway (R-HSA-909733), and interferon signaling pathway (R-HSA-913531) were associated with the immune and inflammatory response. The significant pathways related to immune response and inflammatory response are showed in the Figure 3.

### The protein-to-protein networks of DEGs enriched after infected with ASFV

**Figure 4 -:**
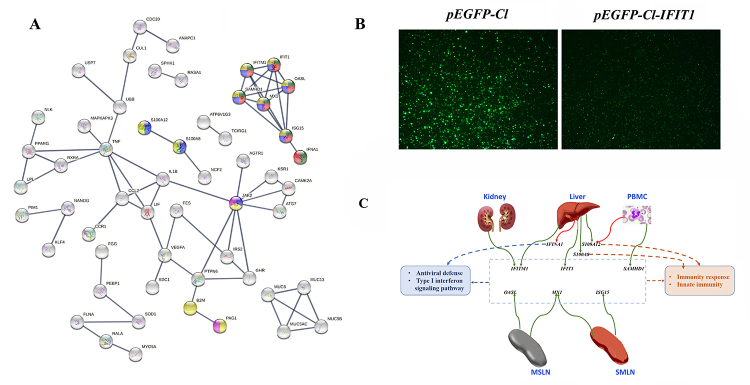
Protein-protein interaction (PPI) predicted networks of DEGs. (A) The PPI network analysis of 118 immune related genes using STRING.11 (confidence 0.9). Each node represents one protein. All the annotated proteins are shown as white nodes and colored nodes represent the proteins involved in immune or antivirus related pathways: red, antiviral defense; yellow, immunity; blue, innate immunity; green, type I interferon signaling pathway; and purple, adaptive immunity. (B) The expression of fluorescent protein in 3D4/21cells transfected plasmid after infected with ASFV SY18. The stronger of fluorescence intensity represents that the more protein of ASFV. (C) The gene expression from significant interaction PPI network in different tissues. The solid red lines mean the DEG was upregulated; the solid green lines mean the DEG is downregulated; The dotted lines mean the pathways that the DEGs were involved in. MSLN is short for mesenteric lymph node; PBMC is short for peripheral blood mononuclear cell; SMLN is short for subman-dibular lymph node. Due to RNA sequencing failure, only 11 samples of SMLN and Spleen were used in this study.

To confirm interaction of tissues, protein to protein analysis of DEGs enriched in the immune related pathways was performed. String 3.0 was used to analyze whether the protein interaction proteins identified in the STRING were interconnected (highest confidence 0.9, Figure 4A). Proteins encoded by *IFIT1, IFITM1, OASL, SAMHD1, MX1,* and *ISG15* are involved in the response to antiviral defense, innate immunity, immunity and type I interferon signaling pathway. Interestingly, these genes were all downregulated when the pigs were infected with ASFV. Gene *S100A12* was downregulated in the liver and spleen but upregulated in the PBMC. Gene *JAK2* was also upregulated in the PBMC after pig infected with PBMC.

### Gene IFIT1 inhibits the replication of ASFV in the cell line 3D4/21

To determine the replication efficiency of ASFV treated *in vitro* with the overexpression of gene *IFIT1*, cell line 3D4/21 were infected with SY18 (MOI = 5). After 48h ASFV infection of cell 3D4/21, the intensity of fluorescence and the proportion of fluorescent cells reflected of the pEGFP-Cl-IFIT1 expression vector significantly increased comparing to the pEGFP-Cl expression vector that transfected to cell 3D4/21 (Fig. 4B). The intensity of fluorescence reflected the inhibitory effect of overexpression of gene *IFIT1* on the virus.

## Discussion

African swine fever leads to negative economic impacts on the pig industry all around world, and it was first reported in Kenya. Several studies reported that some domestic pigs in Kenya showed ASF tolerance (The rate of incidence is less than 50%) (Okoth *et al.*, 2013; Mujibi *et al.*, 2018). Aim of this study was to elucidate whether Kenya domestic pigs have environmental adaptability to ASF and further to characterize the gene expression changes when the pigs were affected with non-lethal ASFV. RNA sequencing was performed to characterize the transcriptome changes from PBMC in Kenya domestic pigs and Landrace × Yorkshire (L/Y) pigs infected with ASFV. As results, only 3 DEGs were identified in the PBMC of Kenya domestic pigs when compared to L/Y pigs before ASFV infection. One month after ASFV infection, there were still only 2 DEGs observed when the two pig populations were compared (Supplementary materials). The results of the clinical presentation and gross pathology also showed no significant difference between Kenya domestic and L/Y pigs infected with ASFV. Taken together, Kenya domestic pigs and L/Y pigs were merged into one population for further analysis.

After merging these two pig populations, a total of 8 ASFV infected pigs and 4 control pigs were used in our transcriptome data. These 8 ASFV infected pigs were compared to 4 control pigs for tissues including kidney, liver, MSLN, SMLN, and spleen. For PBMC, which were isolated from blood, we used RNA data from before and after ASFV infection for each of the 8 infected pigs. Altogether, more than 2000 DEGs were identified in 6 tissues of ASFV infected pigs compared to control pigs. Among these genes, a total of 149 genes were shared in 4, 3 or 2 tissues (Table 2). For these shared DEGs, some of them showed same expression trends in some tissues, others showed opposite trends in other tissues. These genes show their various regulation function in various tissues when the pigs are infected with ASFV. Next, enriched pathways and interaction analysis of proteins encoded by DEGs revealed that genes *IFIT1, IFITM1, MX1, OASL, ISG15, SAMHD1, S100A12* and *IFINA1* were involved in the immunity response or antiviral response (Fig. 4A). Genes *IFIT1, IFITM1, MX1, OASL, ISG15* and *SAMHD1* were all involved in the antiviral defense and type I interferon signaling pathway. The interferon (IFN) system is an early innate antiviral host defense mechanism that occurs shortly after infection by pathogens and before the onset of adaptive immunity. ASFV interferes with the signal transduction pathway that controls the transcription of cytokines. Previous studies have shown that several *in vitro* ASFV proteins can suppress the immune response by reducing interferon induction, interferon response and NF-κB activation (Correia *et al.*, 2013). The ASFV A528R protein inhibits the induction of the *NF-κB* and *IRF3* branches of the type I IFN-induced signaling pathway and the effect of type I and type II interferon stimulation on the IFN response (Correia *et al.*, 2013). *MxA* genes, which were also regulated by IFN pathways, were found to inhibit the replication of ASFV (Netherton *et al.*, 2009). A previous study reported that recombinant porcine IFNs have high antiviral activity against ASFV, and therefore *IFNs* could significantly trigger the production of a variety of IFN-induced genes, including *IFIT1, IFITM3, Mx-1, OASL, ISG15, et al.* (Fan *et al.*, 2020). Obviously, there are 4 overlapping genes in our study. Besides IFN-induced genes, *S100* proteins (*S100A12*, and *S100A8*) was also found to play important roles when hosts are under ASFV invasion. When the study of pigs infected with both a highly virulent (Georgia 2007 strain, GRG) and low (OURT) virulent ASFV was compared with our study, there were 45 and 26 overlapping genes in the current study, such as *IFIT1*, and *S100A8.* In the report of gene expression in macrophages infected with African swine fever virus CN/GS/2018 strain (Cackett *et al.*, 2022), there were 110 overlapping genes with our study. A similar study which was using transcriptome data to characterize the immune response between pigs acutely infected, dead and asymptomatic infection of ASFV in pigs, altogether 67 overlapping immune genes were shared (Sun *et al.*, 2021). Unfortunately, there were no common genes shared in these four studies, which show the complicated immune response when pigs were under ASFV attack (Table S9).

Interestingly, in our study, most of the *IFN* induced genes were downregulated (Figure 4C). But in the previous study, the expression of *IFIT1* was upregulated (Jaing *et al.*, 2017; Cackett *et al.*, 2022). Considering that DEGs in three studies were detected in different tissues and different environment, these results were acceptable. Using the expression of fluorescent protein in *3D4/21* cells transfected plasmid after infected with ASFV SY18 (Figure 4B), overexpression of gene *IFIT1* proved its inhibition of the replication of ASFV.

In our study, all pigs survived to day 23 days post ASFV infection, then all the animals were euthanised. The animals in this study displayed clinical signs that are similar to the low dose group (1 HAD_50_) reported by Hemmink, clinical signs were much milder than the titer of 10^2^ HAD_50_ (Hemmink *et al.*, 2022). ASFV is known to infect pigs of all ages. Most studies prefer using younger pigs for ASFV experimental infection. It has been reported that differences exist between 12 and 18-week old domestic pigs in susceptibility to a moderately virulent ASFV strain (Post *et al.*, 2017), which also differs from another previous study which showed no mortalities in 12-week old pigs (de Carvalho Ferreira *et al.*, 2012). To our best knowledge, pigs (9-10.5 months) used in our study have the oldest age in ASFV experimental infections. Jaing *et al.* (2017) reported the different responses to low and high pathogenic ASFV. Low pathogenic ASFV showed no or few clinical signs; whereas, high pathogenic virus produced clinical signs consistent with acute ASF. The titer in our study is HAD_50_= 10^2.7^/ml, which obviously was a subacute pathogenic titer, which is consistent with a previous review on pathology and pathogenesis of ASF infection in Swine (Salguero, 2020). Determining whether there are age-related or pathogenicity differences of ASFV in the immune transcriptome following ASFV infection in Kenya domestic and L/Y pigs would need to be addressed by a separate study.

In conclusion, we performed RNA-seq in Kenyan domestic pigs and L/Y pigs infected with ASFV to investigate gene expression changes when the pigs were infected with low dose of ASFV. After comparing the shared and specific differentially expressed genes of the two populations, the expression profiles of DEGs shared in the different tissues illustrated the various regulation function in the different tissues. Functional annotation analysis and interaction of proteins encoded by DEGs revealed that the gene expression of DEGs enriched in the immune and antivirus pathways were significantly decreased when the hosts were under ASFV invasion. Proteins encoded by ASFV could inhibit the activity of immune and antivirus regulatory genes, which maybe the reason pigs show weak resistance to ASFV. At last, seven IFN induced genes and two S100 family genes could play crucial roles in the process when the pigs under ASFV attack.

## Supplementary material

The following online material is available for this article:

Supplementary Data S1 - Differentially expressed genes analysis between Kenya domestic pigs and L/Y pigs.

Figure S1 - The original figure that Figure 1B was cropped from.

Table S1 - Summaries of RNA sequencing reads.

Table S2 - The DEGs list of our study.

Table S3 - The enriched pathways of DEGs in kidney using KOBAS 3.0.

Table S4 - The enriched pathways of DEGs in liver using KOBAS 3.0.

Table S5 - The enriched pathways of DEGs in MSLN using KOBAS 3.0.

Table S6 - The enriched pathways of DEGs in PBMC using KOBAS 3.0.

Table S7 - The enriched pathways of DEGs in SMLN using KOBAS 3.0.

Table S8 - The enriched pathways of DEGs in spleen using KOBAS 3.0.

Table S9 - Overlapping genes with our study.
