# Supplementary material for: Transcriptome analysis reveals gene expression changes of pigs infected with non-lethal African swine fever virus
Source: Genet Mol Biol. 2023 Oct 13;46(3):e20230037. doi: 10.1590/1678-4685-GMB-2023-0037 (PMC10578457; doi:10.1590/1678-4685-GMB-2023-0037)
Supplement: Supplementary Data S1 - [file 1415-4757-GMB-46-3-e20230037-s1.pdf]

Genetics and Molecular Biology

https://doi.org/10.1590/1678-4685-GMB-2023-0037

Supplementary Material to "Transcriptome analysis reveals gene expression changes of pigs infected with non-lethal African swine fever virus"

Supplementary Data S1 - Differentially expressed genes analysis between Kenya domestic pigs and L/Y pigs.

To confirm whether Kenya domestic pigs have adaptability to ASF and further to find resistant genes of pigs to ASFV, RNA sequencing was performed to characterize the transcriptome changes from PBMC in Kenya domestic pigs and Landrace × Yorkshire (L/Y) pigs infected with ASFV. Two groups of comparison were carried out for differential expressed genes (DEGs) detection in this study, which were the ASFV infectious of Kenya domestic pigs vs. the ASFV infectious of L/Y pigs ($IN_{KD/LY}$ comparison), and the control group of Kenya domestic pigs vs. the control group of L/Y pigs ($CO_{KD/LY}$ comparison).

As results, only three DEGs in PBMC were found in the $CO_{KD/LY}$ comparison, which reveals that the genetic distance in Kenya domestic and L/Y pigs are close. After ASFV infection, only 3 DEGs were identified between Kenya domestic pigs and L/Y pigs.

Table 1 - The number of DEGs identified in the PBMC between Kenya domestic pigs and L/Y pigs.

		**$IN_{KD/LY}$ comparison**	**$CO_{KD/LY}$ comparison**
	Up	2	2
PBMC	Down	0	1
	Total	2	3
