# Supplementary material for: Transcriptome analysis reveals gene expression changes of pigs infected with non-lethal African swine fever virus
Source: Genet Mol Biol. 2023 Oct 13;46(3):e20230037. doi: 10.1590/1678-4685-GMB-2023-0037 (PMC10578457; doi:10.1590/1678-4685-GMB-2023-0037)
Supplement: Table S1 - [file 1415-4757-GMB-46-3-e20230037-s3.pdf]

Genetics and Molecular Biology

https://doi.org/10.1590/1678-4685-GMB-2023-0037

Supplementary Material to "Transcriptome analysis reveals gene expression changes of pigs infected with non-lethal African swine fever virus"

Table S1 - Summaries of RNA sequencing reads.

Sample ID	Group	Breed	Paried Reads	Average % Q30
KD1_Kidney	Control_pigs	Kenyan domestic	41679006	93.35
KD1_Liver	Control_pigs	Kenyan domestic	39103879	93.48
KD1_MSLN	Control_pigs	Kenyan domestic	44491554	91.98
KD1_SMLN	Control_pigs	Kenyan domestic	37168077	92.09
KD2_Kidney	Control_pigs	Kenyan domestic	39070140	91.92
KD2_Liver	Control_pigs	Kenyan domestic	39618527	89.14
KD2_MSLN	Control_pigs	Kenyan domestic	37403481	91.75
KD2_SMLN	Control_pigs	Kenyan domestic	42037998	78.32
KD2_Spleen	Control_pigs	Kenyan domestic	33886057	93.38
L/Y1_Kidney	Control_pigs	Landrace × Yorkshire	32846310	92.02
L/Y1_Liver	Control_pigs	Landrace × Yorkshire	36400694	93.13
L/Y1_MSLN	Control_pigs	Landrace × Yorkshire	40065267	92.51
L/Y1_SMLN	Control_pigs	Landrace × Yorkshire	41111751	90.67
L/Y1_Spleen	Control_pigs	Landrace × Yorkshire	46831364	92.46
L/Y2_Kidney	Control_pigs	Landrace × Yorkshire	49881148	93.48
L/Y2_Liver	Control_pigs	Landrace × Yorkshire	40878008	93
L/Y2_MSLN	Control_pigs	Landrace × Yorkshire	49553191	91.76
L/Y2_Spleen	Control_pigs	Landrace × Yorkshire	51463850	92.93
KD3_Kidney	Infectious_pigs	Kenyan domestic	47228680	93.06
KD3_Liver	Infectious_pigs	Kenyan domestic	39967422	91.29
KD3_MSLN	Infectious_pigs	Kenyan domestic	46798725	89.18
KD3_PBMC_04	infectious_pigs	Kenyan domestic	46467579	90.16
KD3_PBMC_05	infectious_pigs	Kenyan domestic	37994495	91.46
KD3_SMLN	infectious_pigs	Kenyan domestic	42422548	89.69
KD3_Spleen	infectious_pigs	Kenyan domestic	50083833	90.11
KD4_Kidney	infectious_pigs	Kenyan domestic	47273463	91.85
KD4_Liver	infectious_pigs	Kenyan domestic	45339390	91.68
KD4_MSLN	infectious_pigs	Kenyan domestic	27899183	90.62
KD4_PBMC_04	infectious_pigs	Kenyan domestic	47803838	91.61
KD4_PBMC_05	infectious_pigs	Kenyan domestic	52668816	76.93
KD4_SMLN	infectious_pigs	Kenyan domestic	50511999	87.44

Sample ID	Group	Breed	Paried Reads	Average % Q30
KD4_Spleen	infectious_pigs	Kenyan domestic	52363816	90.99
KD5_Kidney	infectious_pigs	Kenyan domestic	49469349	92.48
KD5_Liver	infectious_pigs	Kenyan domestic	53520767	90.1
KD5_MSLN	infectious_pigs	Kenyan domestic	45726341	91.31
KD5_PBMC_04	infectious_pigs	Kenyan domestic	48554137	92.7
KD5_PBMC_05	infectious_pigs	Kenyan domestic	46404291	90.31
KD5_SMLN	infectious_pigs	Kenyan domestic	53423923	92.93
KD5_Spleen	infectious_pigs	Kenyan domestic	50802647	89.13
KD6_Kidney	infectious_pigs	Kenyan domestic	51920609	91.59
KD6_Liver	infectious_pigs	Kenyan domestic	54045166	92.06
KD6_MSLN	infectious_pigs	Kenyan domestic	39854371	92.52
KD6_PBMC_04	infectious_pigs	Kenyan domestic	43127612	91.63
KD6_PBMC_05	infectious_pigs	Kenyan domestic	49328669	92.28
KD6_SMLN	Infectious_pigs	Kenyan domestic	49654405	91.33
KD6_Spleen	Infectious_pigs	Kenyan domestic	45338705	90.59
L/Y3_Kidney	Infectious_pigs	Landrace × Yorkshire	57760701	93.75
L/Y3_Liver	Infectious_pigs	Landrace × Yorkshire	50765882	92
L/Y3_MSLN	Infectious_pigs	Landrace × Yorkshire	47505609	92.22
L/Y3_PBMC_04	infectious_pigs	Landrace × Yorkshire	48884019	92.25
L/Y3_PBMC_05	infectious_pigs	Landrace × Yorkshire	45795016	92.86
L/Y3_SMLN	Infectious_pigs	Landrace × Yorkshire	49752937	89.23
L/Y3_Spleen	Infectious_pigs	Landrace × Yorkshire	44429928	91.86
L/Y4_Kidney	Infectious_pigs	Landrace × Yorkshire	38811913	92.8
L/Y4_Liver	Infectious_pigs	Landrace × Yorkshire	41773474	91.11
L/Y4_MSLN	Infectious_pigs	Landrace × Yorkshire	44423902	92.86
L/Y4_PBMC_04	infectious_pigs	Landrace × Yorkshire	54782776	92.42
L/Y4_PBMC_05	infectious_pigs	Landrace × Yorkshire	43933138	92.09
L/Y4_SMLN	Infectious_pigs	Landrace × Yorkshire	41800676	92.5
L/Y4_Spleen	Infectious_pigs	Landrace × Yorkshire	61655853	91.4
L/Y5_Kidney	Infectious_pigs	Landrace × Yorkshire	52497152	93.62
L/Y5_Liver	Infectious_pigs	Landrace × Yorkshire	43538274	92.49
L/Y5_MSLN	Infectious_pigs	Landrace × Yorkshire	30562368	87.82
L/Y5_PBMC_04	infectious_pigs	Landrace × Yorkshire	41232638	91.59
L/Y5_PBMC_05	infectious_pigs	Landrace × Yorkshire	45674612	91.38
L/Y5_SMLN	Infectious_pigs	Landrace × Yorkshire	47148470	91.8
L/Y5_Spleen	Infectious_pigs	Landrace × Yorkshire	45962808	91.66
L/Y6_Kidney	Infectious_pigs	Landrace × Yorkshire	47211172	91.73
L/Y6_Liver	Infectious_pigs	Landrace × Yorkshire	49163397	92.7
L/Y6_MSLN	Infectious_pigs	Landrace × Yorkshire	55298009	89.89
L/Y6_PBMC_04	infectious_pigs	Landrace × Yorkshire	35388477	89.89
L/Y6_PBMC_05	infectious_pigs	Landrace × Yorkshire	38865728	83.93
L/Y6_SMLN	Infectious_pigs	Landrace × Yorkshire	40297635	92.12
L/Y6_Spleen	Infectious_pigs	Landrace × Yorkshire	54529070	91.61
