# Supplementary material for: Transcriptome analysis reveals gene expression changes of pigs infected with non-lethal African swine fever virus
Source: Genet Mol Biol. 2023 Oct 13;46(3):e20230037. doi: 10.1590/1678-4685-GMB-2023-0037 (PMC10578457; doi:10.1590/1678-4685-GMB-2023-0037)
Supplement: Table S2 - [file 1415-4757-GMB-46-3-e20230037-s4.pdf]

Genetics and Molecular Biology
https://doi.org/10.1590/1678-4685-GMB-2023-0037

Supplementary Material to "Transcriptome analysis reveals gene expression changes of pigs infected with non-lethal African swine fever virus"

Table S2 - The DEGs list of our study.

tissue	transcript_id	log2FoldChange	regulation	pvalue	padj	gene_sympo
kidney	rna44904	-5.145992246	down	2.71607E-09	8.72324E-06	PDK4
kidney	rna49827	-4.367637508	down	0.000286552	0.007034136	LOC100626416
kidney	rna70931	-3.670127395	down	8.54965E-25	1.27527E-20	CYP24A1
kidney	rna20114	-3.409009233	down	2.50841E-05	0.002069958	LOC100514834
kidney	rna56663	-3.269114873	down	0.002212388	0.019007574	LOC100739719
kidney	rna195	-3.148075135	down	2.92412E-09	8.72324E-06	PLG
kidney	rna43174	-3.046450707	down	5.45253E-09	9.85095E-06	MMP7
kidney	rna10444	-2.889712526	down	6.1738E-09	9.85095E-06	ANGPTL4
kidney	rna8982	-2.840449535	down	2.1915E-05	0.001969183	LYVE1
kidney	rna42286	-2.715827005	down	0.000459055	0.008689419	LOC100515788
kidney	rna43568	-2.714019444	down	9.94854E-07	0.000372048	APOA4
kidney	rna58246	-2.620119075	down	0.000129465	0.004829574	COX17
kidney	rna78496	-2.588877625	down	0.000654058	0.010478975	LOC110259135
kidney	rna40655	-2.559934449	down	0.002240564	0.019097286	FGB
kidney	rna37367	-2.545519933	down	3.93661E-08	3.45403E-05	LOC110261577
kidney	rna19698	-2.544834983	down	2.62468E-06	0.000664475	FABP4
kidney	rna20306	-2.534470712	down	9.02584E-05	0.004030822	DPT
kidney	rna59035	-2.494195116	down	1.28961E-06	0.000409273	LOC102158685
kidney	rna37872	-2.462607471	down	0.000205353	0.006077472	RNASE6
kidney	rna74144	-2.431483899	down	5.91318E-05	0.003105668	LOC110257723
kidney	rna9666	-2.413895685	down	0.000457046	0.008689419	LOC100739101
kidney	rna59642	-2.407670395	down	1.31821E-05	0.001614708	LPL
kidney	rna43434	-2.405825339	down	2.32476E-07	0.00012929	PLET1
kidney	rna25042	-2.401757067	down	1.16459E-07	9.14263E-05	SMCO2
kidney	rna19644	-2.387484453	down	3.30328E-05	0.002391829	CA3
kidney	rna40574	-2.384072927	down	0.000228639	0.006367244	CXCL10
kidney	rna35153	-2.371505423	down	0.000881472	0.012118002	LOC100154508
kidney	rna11703	-2.355879569	down	0.000567199	0.009780735	THBS4
kidney	rna68324	-2.326679774	down	9.27347E-06	0.001340073	LOC110257331
kidney	rna35149	-2.308554643	down	4.15624E-05	0.002649335	LOC100154071
kidney	rna40661	-2.252812544	down	1.07455E-06	0.000390926	SFRP2
kidney	rna59764	-2.197740856	down	0.000154949	0.005288829	ADAM7
kidney	rna32754	-2.176626781	down	0.000118136	0.004674045	RNF125
kidney	rna35234	-2.16526683	down	0.000341679	0.00784075	LOC106504234
kidney	rna56800	-2.140308773	down	1.89919E-06	0.000544776	TM4SF4
kidney	rna4069	-2.125393375	down	1.05678E-05	0.001454692	RPL36AL

tissue	transcript_id	log2FoldChange	regulation	pvalue	padj	gene_sympo
kidney	rna36927	-2.091064175	down	0.001860481	0.017548988	PLIN1
kidney	rna41300	-2.066024889	down	0.003715852	0.024810049	LOC100737472
kidney	rna32753	-2.052182866	down	0.000264301	0.006785399	RNF125
kidney	rna35152	-2.046585078	down	5.74127E-06	0.000990962	LOC100525821
kidney	rna37101	-2.04435374	down	0.0002855	0.007034136	RPP25
kidney	rna6659	-2.039060032	down	0.002456531	0.020022738	FCN2
kidney	rna6712	-2.033811722	down	1.74324E-05	0.001805708	IFITM1
kidney	rna59036	-2.0296954	down	0.011614556	0.047659618	LOC102158685
kidney	rna59758	-2.022046539	down	1.22239E-06	0.000396372	STC1
kidney	rna197	-2.021363057	down	2.41755E-05	0.002022092	SLC22A3
kidney	rna38897	-2.01923329	down	5.04531E-06	0.00095668	SERPINA3-2
kidney	rna60391	2.001100321	up	0.000992315	0.012893181	PITPNM2
kidney	rna74783	2.005219536	up	0.000489964	0.008978254	ARMCX4
kidney	gene26056	2.010099399	up	5.80453E-05	0.003090833	LOC106506037
kidney	gene4482	2.011118048	up	0.000275356	0.00693953	LOC110259404
kidney	rna22936	2.012455742	up	0.000910014	0.012328576	SHANK3
kidney	rna47839	2.013567235	up	0.0080864	0.038572671	FRMD4A
kidney	rna62770	2.013802828	up	0.000607897	0.010211025	LOC110256824
kidney	rna78307	2.017140054	up	0.007353009	0.036559159	LOC110259044
kidney	rna18594	2.017522424	up	0.000395584	0.008285351	IQANK1
kidney	rna26734	2.02317745	up	0.001578673	0.016283872	LOC110260827
kidney	rna21923	2.026070556	up	0.000163384	0.005454418	VTCN1
kidney	rna15076	2.026836596	up	3.53939E-05	0.002479384	SEPT12
kidney	rna31022	2.028828847	up	0.000284592	0.007034136	MTHFR
kidney	rna70023	2.030001309	up	0.000258374	0.006718188	NOL4L
kidney	rna77308	2.042718355	up	0.002627564	0.02061691	AHNAK2
kidney	rna48129	2.042833898	up	4.02854E-05	0.002649335	GJD4
kidney	rna48144	2.045592469	up	0.000799803	0.011460505	CAMK1D
kidney	rna48089	2.048429753	up	0.000191568	0.005863351	LOC110255706
kidney	rna68760	2.053643981	up	0.000848584	0.011796345	LOC106506544
kidney	rna13430	2.05405798	up	0.000729711	0.010950072	LOC106509613
kidney	rna18523	2.054257199	up	0.000124035	0.004749244	WDR97
kidney	rna76954	2.056608186	up	7.09353E-05	0.003503547	LOC110258199
kidney	rna2083	2.070432401	up	0.000281752	0.007018701	MYO5B
kidney	rna27259	2.075883367	up	0.001151763	0.013899434	LOC100739735
kidney	rna13558	2.077621724	up	0.000742878	0.011018947	LOC110259841
kidney	rna67457	2.079651804	up	0.000274298	0.00693953	DNAJB3
kidney	rna62531	2.084300192	up	0.001341292	0.015071087	LOC110256662
kidney	rna53734	2.086091147	up	0.000613784	0.01022928	LOC110256117
kidney	rna48678	2.086223422	up	0.0035261	0.024170635	SLC7A1
kidney	rna544	2.097083992	up	0.001593095	0.016366296	ZC2HC1B
kidney	rna17484	2.103641369	up	0.000196811	0.00591862	TMEM178A
kidney	rna48588	2.105127337	up	0.000532827	0.009495398	LOC102162170
kidney	rna51597	2.10717834	up	5.2612E-06	0.000959275	KRT39
kidney	rna72122	2.117624023	up	0.008665906	0.040173738	LOC106508208
kidney	rna34931	2.124468801	up	0.000402948	0.008359227	LOC100153329
kidney	rna53724	2.127232545	up	1.07811E-05	0.001454692	LOC102157639

tissue	transcript_id	log2FoldChange	regulation	pvalue	padj	gene_sympo
kidney	rna56024	2.129010657	up	0.000108475	0.004422126	LOC106507990
kidney	rna7190	2.132388288	up	0.000886235	0.012161067	RPS6KB2
kidney	rna19336	2.133795024	up	0.004799212	0.028692162	NCALD
kidney	rna48384	2.138795797	up	0.000932958	0.012525649	ADARB2
kidney	rna70957	2.138966225	up	0.007134331	0.035819045	FAM209B
kidney	rna53723	2.141539942	up	0.001078508	0.013385693	LOC102157481
kidney	rna15560	2.14286908	up	0.00138581	0.015245679	TELO2
kidney	rna50319	2.154577677	up	0.00333997	0.02338248	CEP295NL
kidney	rna48589	2.158496843	up	0.001006086	0.012948035	LOC110255794
kidney	rna69529	2.161409801	up	0.000531961	0.009495283	LOC102160165
kidney	rna17827	2.165256668	up	0.002764193	0.021197498	MAPRE3
kidney	rna7000	2.169621309	up	0.000835251	0.01172023	LOC102159152
kidney	rna64000	2.17276051	up	5.72992E-05	0.003090833	LOC102163817
kidney	rna15589	2.176404963	up	0.000951728	0.012606932	UNKL
kidney	rna46612	2.18083621	up	0.000141325	0.005018187	ESRRG
kidney	rna77860	2.181199581	up	0.000380464	0.008136675	LOC102164320
kidney	rna19326	2.182061763	up	0.001345921	0.015071668	NCALD
kidney	rna30077	2.182441662	up	0.000794613	0.011440587	SSC5D
kidney	gene10385	2.187313788	up	0.004110824	0.026350261	LOC110261349
kidney	rna24334	2.197801473	up	0.004515434	0.027637344	PAN2
kidney	rna49144	2.212439802	up	0.000175547	0.005625246	LOC110255812
kidney	rna9395	2.214501201	up	0.001457993	0.015591072	UBA52
kidney	rna27647	2.216571694	up	5.77797E-05	0.003090833	LOC106507572
kidney	rna52466	2.219965373	up	0.000136639	0.004929109	C12H17orf50
kidney	rna6564	2.232353728	up	0.005891983	0.032109907	LOC110256518
kidney	rna34747	2.243976573	up	0.000424693	0.008459989	JARID2
kidney	gene1814	2.253756567	up	9.93433E-06	0.001397929	LOC106506973
kidney	rna62010	2.254127395	up	7.54412E-05	0.003641686	RHOBTB1
kidney	rna65208	2.263710218	up	0.001931977	0.017839204	C15H4orf47
kidney	rna12032	2.264744555	up	4.77307E-05	0.002859242	LOC102162728
kidney	rna42171	2.271793374	up	0.00133295	0.015006561	LOC110255363
kidney	rna13232	2.280320945	up	9.55481E-05	0.004215521	IL17B
kidney	rna23244	2.28203162	up	5.33788E-06	0.000959275	LOC110260835
kidney	rna19329	2.284926054	up	0.000390511	0.008195822	NCALD
kidney	gene7189	2.285749972	up	5.69836E-05	0.003090833	LOC100526166
kidney	rna69806	2.299897552	up	0.001358216	0.015141363	GNRH2
kidney	rna27183	2.301936489	up	2.33588E-07	0.00012929	LOC106510470
kidney	rna15714	2.311038001	up	5.5509E-05	0.003090833	NME4
kidney	rna61542	2.313321523	up	2.07094E-05	0.001913018	LRRC74B
kidney	rna36127	2.316858727	up	0.000633405	0.010291969	LOC110261516
kidney	rna26157	2.317518905	up	0.000742475	0.011018947	LOC110260776
kidney	rna53241	2.319734194	up	0.002526949	0.020206984	ZMYND15
kidney	rna17905	2.324515548	up	8.40138E-05	0.003945879	DNMT3A
kidney	rna76123	2.325694881	up	0.001250993	0.01442007	GAB3
kidney	rna64844	2.328381765	up	0.00046255	0.008728804	GLI2
kidney	rna19337	2.339234437	up	0.000226262	0.006367244	LOC110260427
kidney	rna27260	2.340091996	up	0.00070809	0.010827003	LOC100621820

tissue	transcript_id	log2FoldChange	regulation	pvalue	padj	gene_sympo
kidney	rna46597	2.343423403	up	4.839E-05	0.00287564	ESRRG
kidney	rna44539	2.355854539	up	0.000125438	0.004773043	PM20D1
kidney	rna49850	2.361817375	up	0.000671671	0.010506733	ING1
kidney	rna14033	2.369933332	up	0.001426201	0.015448957	RASA4B
kidney	rna41183	2.373905482	up	0.001551221	0.016092645	TRPC3
kidney	rna52664	2.385221756	up	1.67528E-06	0.00048997	NOS2
kidney	rna71704	2.393123124	up	5.84628E-05	0.003092311	TAS2R39
kidney	rna71539	2.393899182	up	7.54746E-07	0.000312717	WDR86
kidney	rna53735	2.401178085	up	1.57384E-05	0.001713534	LOC110256117
kidney	rna26922	2.407665227	up	0.000946016	0.012565246	LOC110260879
kidney	rna3093	2.410558417	up	1.17925E-06	0.000396372	SPTBN5
kidney	rna58089	2.419624851	up	0.000420263	0.008425595	MYLK
kidney	rna19327	2.423387253	up	0.000475323	0.008840305	NCALD
kidney	rna7253	2.433950998	up	0.000100623	0.004276035	C2H11orf80
kidney	rna53585	2.438518993	up	0.008256055	0.039081981	DNAH9
kidney	rna71707	2.45163202	up	4.50523E-06	0.000906048	LOC100621797
kidney	rna52754	2.452552716	up	4.71453E-05	0.002845153	LOC100515508
kidney	rna60779	2.452647837	up	0.000500632	0.009084465	C14H12orf49
kidney	rna27080	2.499975661	up	0.000978106	0.012764153	ATP2C2
kidney	rna32570	2.502773325	up	0.00031525	0.007490203	ENOSF1
kidney	rna52693	2.50490049	up	0.000346402	0.00789303	LOC106505483
kidney	rna73253	2.506906523	up	0.008253805	0.039081981	GPR143
kidney	rna49836	2.510914368	up	3.44747E-06	0.000756212	COL4A2
kidney	rna10742	2.515848181	up	0.006740208	0.034608244	UBXN6
kidney	rna15042	2.517954079	up	0.00078567	0.01137732	LOC110260090
kidney	rna78219	2.522241612	up	0.001731991	0.016983729	LOC100525026
kidney	rna46032	2.526201393	up	0.002478453	0.020091632	ZNF648
kidney	rna13227	2.526794775	up	6.38179E-05	0.003248834	IL17B
kidney	rna54538	2.528072104	up	0.001246301	0.014414036	LOC110256583
kidney	rna54338	2.539608234	up	0.001240189	0.01437347	CCR8
kidney	rna34927	2.55399814	up	1.89026E-05	0.00186723	SCGN
kidney	rna64001	2.563575874	up	0.001887528	0.017673801	LOC110256796
kidney	rna33585	2.566773037	up	2.34033E-07	0.00012929	LOC102158888
kidney	rna46721	2.574812025	up	0.004250727	0.026809238	DUSP10
kidney	rna6834	2.577780067	up	9.689E-06	0.001376392	MUC6
kidney	rna6995	2.589115285	up	0.000112705	0.004543515	LOC100627067
kidney	rna50356	2.591719688	up	0.000154935	0.005288829	TMC8
kidney	rna35062	2.598296052	up	0.006184919	0.032971497	LOC110261659
kidney	rna39458	2.599622888	up	0.000164527	0.005454418	LOC110262057
kidney	rna68338	2.610042971	up	0.000126457	0.004777589	LOC110257245
kidney	rna23245	2.613067516	up	2.60989E-05	0.002104275	LOC110260623
kidney	rna17692	2.635603206	up	0.000938294	0.012552434	ALK
kidney	rna50372	2.650555864	up	0.000143988	0.005053455	TNRC6C
kidney	gene9317	2.678329382	up	0.012000439	0.048614489	LOC110260705
kidney	rna36667	2.679494394	up	4.03677E-11	2.00708E-07	LOC100158035
kidney	rna52309	2.7048646	up	0.003005364	0.022192082	USP32
kidney	rna24820	2.707588564	up	0.012039572	0.048693671	LOC110260706

tissue	transcript_id	log2FoldChange	regulation	pvalue	padj	gene_sympo
kidney	rna43353	2.717497038	up	0.004875954	0.0289281	COLCA2
kidney	rna43921	2.732479739	up	0.000135591	0.004920871	UBASH3B
kidney	rna17361	2.735448469	up	1.3361E-05	0.001614708	LOC110260021
kidney	rna24094	2.739639889	up	1.51648E-05	0.001675539	HOXC4
kidney	rna49967	2.76638062	up	1.20517E-06	0.000396372	LOC106504762
kidney	rna46035	2.792150174	up	0.000444639	0.008624484	GLUL
kidney	rna42324	2.798833509	up	0.004808775	0.028714049	LOC100513367
kidney	rna52662	2.867115397	up	0.000148731	0.005171267	NOS2
kidney	rna51037	2.888945507	up	1.4057E-05	0.001650973	ACE
kidney	rna47947	2.89966554	up	0.000113439	0.004560207	LOC110255698
kidney	rna71174	2.902862194	up	0.001308882	0.014857366	SLCO4A1
kidney	rna322	2.9039772	up	0.000129591	0.004829574	TIAM2
kidney	rna75370	2.939654863	up	0.000371284	0.008122208	LOC110257857
kidney	rna14492	2.952377784	up	8.54111E-05	0.003968821	FAM57B
kidney	rna60715	2.988191069	up	0.000130421	0.004829574	TMEM233
kidney	gene12479	3.026428765	up	0.000133404	0.004896114	LOC100512969
kidney	gene9613	3.056940308	up	8.963E-05	0.004030822	LOC100516803
kidney	rna48307	3.076881631	up	0.000612241	0.01022928	LOC106505211
kidney	rna71088	3.161036883	up	0.000134247	0.004896114	NELFCD
kidney	rna72162	3.190829405	up	0.000180682	0.00569779	LOC110257580
kidney	rna49598	3.197301598	up	0.000682066	0.010575565	LOC110255845
kidney	gene29824	3.2526602	up	0.006522248	0.034004143	LOC100738057
kidney	gene19861	3.397605535	up	0.003573942	0.024353094	LOC110256086
kidney	rna53008	3.532775281	up	0.001674972	0.016711631	LOC100627505
kidney	rna78117	3.544147505	up	0.000106886	0.004422126	LOC110258915
kidney	rna5187	3.677606019	up	0.000380763	0.008136675	FAM205C
kidney	rna6592	3.726341212	up	1.3227E-05	0.001614708	FAM163B
kidney	rna39609	3.7555896	up	4.06834E-05	0.002649335	LOC110261972
kidney	rna67947	3.760093888	up	0.002326015	0.019377261	LOC106506472
kidney	rna42640	3.814619329	up	0.003720742	0.024831584	LOC102161477
kidney	rna76992	4.796044368	up	0.006884902	0.035097468	LOC110258225
kidney	rna22898	5.830942246	up	0.000403503	0.008359227	LOC106510256
liver	rna72922	-7.984991365	down	4.42101E-13	2.10101E-09	IGFBP1
liver	rna49599	-5.371045029	down	9.43023E-06	0.0007511	LOC100738425
liver	rna6836	-4.943256217	down	3.18788E-05	0.001561843	MUC5AC
liver	rna78450	-4.798163251	down	5.82322E-06	0.000580571	LOC110259106
liver	id768417	-4.64708281	down	0.000108401	0.003206373	LOC110256717
liver	rna29313	-4.452469268	down	0.000289755	0.006064499	FGF21
liver	rna28283	-3.579617749	down	0.000378493	0.007134279	HAMP
liver	rna3122	-3.543912988	down	0.000611257	0.009300634	CHAC1
liver	rna74728	-3.462813789	down	0.000325677	0.006512163	LOC110257759
liver	rna35271	-3.374404569	down	0.010603899	0.048439533	LOC100513601
liver	rna21361	-3.327568607	down	1.17756E-07	3.57202E-05	S100A8
liver	rna43568	-3.276811699	down	5.69671E-10	9.02422E-07	APOA4
liver	rna49660	-3.235752106	down	0.000154201	0.004116942	SLC15A1
liver	rna54512	-3.228440839	down	0.002460435	0.020627862	CYP8B1
liver	rna63003	-3.184018268	down	4.52714E-06	0.000488966	LOC100157486

tissue	transcript_id	log2FoldChange	regulation	pvalue	padj	gene_sympo
liver	rna64115	-3.12332588	down	5.26144E-05	0.002060779	DMBT1
liver	rna72925	-3.045584649	down	0.000851933	0.011342536	ADCY1
liver	rna69295	-2.940168054	down	2.05577E-09	1.95394E-06	IDO1
liver	rna52456	-2.939095034	down	2.70917E-05	0.001425261	LOC100516039
liver	rna43564	-2.935814838	down	3.03762E-08	1.23916E-05	APOA5
liver	rna10487	-2.847099596	down	0.001196457	0.013712134	CD209
liver	rna20279	-2.831173107	down	0.000171493	0.004424679	SELL
liver	rna4068	-2.784861914	down	1.08062E-05	0.000815152	LRR1
liver	rna58272	-2.762107673	down	0.000307804	0.00627805	UPK1B
liver	rna21346	-2.760563135	down	9.01496E-08	2.92105E-05	S100A2
liver	rna34541	-2.754359887	down	5.21526E-05	0.002048317	F13A1
liver	rna71375	-2.754260275	down	0.000479246	0.00821227	VIPR2
liver	rna3643	-2.717838935	down	3.98129E-05	0.001784947	RNF152
liver	rna72924	-2.69303712	down	0.000556444	0.008994576	LOC106506857
liver	rna21362	-2.664672661	down	9.35704E-05	0.002912738	S100A12
liver	rna58976	-2.659302544	down	0.001729461	0.016967384	BTG3
liver	rna38890	-2.61128642	down	0.00599337	0.034547825	LOC106504547
liver	rna38201	-2.576354277	down	5.21465E-05	0.002048317	ARG2
liver	rna34982	-2.553072091	down	9.27245E-06	0.000742823	LOC110261735
liver	rna26099	-2.53118662	down	7.73181E-05	0.002575523	SLC38A2
liver	rna70583	-2.516912351	down	0.000265612	0.005732517	SLPI
liver	rna21379	-2.51091399	down	0.001129977	0.013172592	LOC100157968
liver	rna70412	-2.490229942	down	0.001336181	0.01466507	LBP
liver	rna54649	-2.485871369	down	1.64781E-08	8.03293E-06	CCR1
liver	rna51445	-2.469803872	down	2.03586E-05	0.001245718	G6PC
liver	rna77795	-2.465668328	down	0.001876329	0.017774636	LOC110258667
liver	rna38896	-2.435078042	down	0.000371964	0.007042615	LOC100156325
liver	rna15333	-2.42098306	down	0.00368918	0.025656895	PAQR4
liver	rna62176	-2.399796725	down	2.32531E-06	0.000304147	SRGN
liver	rna14660	-2.389037001	down	3.04206E-08	1.23916E-05	NDUFAB1
liver	rna27356	-2.389017392	down	1.57659E-09	1.72903E-06	TAT
liver	rna22253	-2.374715495	down	2.94195E-05	0.00150715	GSTM3
liver	rna6712	-2.362272873	down	3.28363E-05	0.001597774	IFITM1
liver	rna68086	-2.350118096	down	0.000912405	0.011655274	FST
liver	rna23803	-2.336524039	down	0.007909426	0.040483248	TMBIM6
liver	rna35269	-2.332508305	down	0.000853458	0.011342536	LOC100515902
liver	rna35152	-2.317807994	down	1.9081E-06	0.000259084	LOC100525821
liver	rna37872	-2.289657557	down	5.06076E-05	0.002031918	RNASE6
liver	rna44934	-2.28061335	down	0.001535314	0.015919255	ASNS
liver	rna21137	-2.253895474	down	4.95022E-06	0.000515148	EFNA1
liver	rna36082	-2.253240742	down	1.29571E-05	0.000927059	PIM1
liver	rna7929	-2.245338406	down	0.004556607	0.029276045	LOC110259471
liver	rna9832	-2.241200932	down	1.38073E-05	0.000956634	ADGRE3
liver	rna55941	-2.23237904	down	0.001720492	0.016939957	EIF4E3
liver	rna41959	-2.206415167	down	0.000320156	0.006446982	BMP3
liver	rna56788	-2.183848676	down	0.009534716	0.04563157	HPS3
liver	rna61497	-2.18018637	down	3.62672E-05	0.001700861	SDF2L1

tissue	transcript_id	log2FoldChange	regulation	pvalue	padj	gene_sympo
liver	rna34880	-2.179819457	down	0.005138235	0.031480799	DCDC2
liver	rna77177	-2.17934897	down	0.003848153	0.026401883	LOC100518848
liver	rna3601	-2.176872914	down	1.42793E-07	4.1547E-05	SERPINB7
liver	rna40651	-2.173963289	down	0.000716959	0.01023191	FGG
liver	rna44667	-2.172828394	down	8.80132E-06	0.000721151	STEAP4
liver	rna52257	-2.170743256	down	0.000688898	0.009981317	CLTC
liver	rna70608	-2.165504983	down	0.009052427	0.044123231	WFDC2
liver	rna15779	-2.16336743	down	0.000485565	0.008270853	LOC110259958
liver	rna59316	-2.162784675	down	0.000128647	0.003603367	ETS2
liver	rna8776	-2.152893088	down	0.000261469	0.005682567	NUCB2
liver	rna3714	-2.134744639	down	2.81885E-06	0.00034349	MIR122
liver	rna2892	-2.133934863	down	1.56008E-05	0.001034512	B2M
liver	rna44593	-2.126831071	down	3.66434E-05	0.001710415	PIGR
liver	rna3288	-2.122556168	down	0.00029975	0.006154193	ACTC1
liver	rna35157	-2.122344815	down	0.002086433	0.018874544	LOC110261664
liver	rna23923	-2.12206959	down	0.000725749	0.010275081	KRT5
liver	gene12972	-2.115623791	down	2.38476E-05	0.001317179	LOC110261480
liver	rna36458	-2.112089217	down	8.85563E-06	0.000721455	CLIC5
liver	rna2307	-2.108849974	down	0.003071325	0.023124803	PCLAF
liver	rna54535	-2.10172867	down	3.21165E-05	0.001568101	ABHD5
liver	rna63036	-2.098561788	down	4.2325E-05	0.001847354	IFIT1
liver	id968230	-2.089261478	down	0.003429593	0.024676135	LOC110258820
liver	rna39817	-2.08755604	down	0.005960797	0.034423983	UCHL1
liver	rna32039	-2.07783889	down	2.55669E-05	0.001365198	GJA4
liver	rna58978	-2.072559042	down	2.74065E-08	1.18405E-05	C13H21orf91
liver	rna18828	-2.070048286	down	1.73859E-09	1.77051E-06	NDRG1
liver	rna26096	-2.062021594	down	0.002033389	0.018631125	SLC38A1
liver	rna77972	-2.06139797	down	0.003357896	0.024383699	LOC100038328
liver	rna40652	-2.058213646	down	0.000980264	0.012158009	FGG
liver	rna46101	-2.050808788	down	8.44327E-08	2.79943E-05	NCF2
liver	rna42286	-2.045552797	down	0.00372981	0.025810909	LOC100515788
liver	rna70443	-2.033036813	down	0.005419516	0.03234931	FAM83D
liver	rna29187	-2.032047678	down	9.31983E-08	2.95273E-05	C5AR1
liver	rna25534	-2.031478979	down	0.004632159	0.029592559	CLEC2D
liver	rna1804	-2.029814491	down	0.006792297	0.037027888	LOC100152150
liver	rna47587	-2.027528563	down	0.005504869	0.032714846	MKX
liver	rna5457	-2.022625732	down	0.002811506	0.022133432	GALNT12
liver	rna39438	-2.018106057	down	0.000188752	0.004712849	HS3ST1
liver	rna40370	-2.016106411	down	0.00012229	0.003459314	LOC100624541
liver	rna49830	-2.009610317	down	8.87454E-07	0.00015789	IRS2
liver	rna26433	2.002677331	up	0.00324758	0.023841783	LOC102160458
liver	rna7579	2.002972234	up	0.002932581	0.022526839	FLRT1
liver	rna50051	2.004057	up	1.47731E-05	0.000993494	LOC110255920
liver	rna56022	2.007145844	up	0.000251985	0.005544058	LOC106505659
liver	rna36858	2.007998701	up	0.000159797	0.004226768	LOC106510116
liver	rna60844	2.011661692	up	7.40414E-06	0.000676279	TPCN1
liver	rna32570	2.01170469	up	0.00205262	0.018687231	ENOSF1

tissue	transcript_id	log2FoldChange	regulation	pvalue	padj	gene_sympo
liver	rna5407	2.012182565	up	0.000503989	0.008493341	CCDC180
liver	rna15067	2.012505112	up	0.008052934	0.040893579	ROGDI
liver	rna28915	2.013105142	up	0.005255299	0.031855782	ZNF283
liver	gene5460	2.013280546	up	0.002879216	0.022357831	LOC102158711
liver	rna24210	2.013652614	up	0.001924226	0.018131982	LOC110260681
liver	rna48038	2.014087767	up	5.48704E-06	0.000561434	NEBL
liver	rna72034	2.017026877	up	0.000509757	0.008550118	LOC110257573
liver	rna6100	2.018455796	up	0.010222355	0.047474632	LOC110256243
liver	rna48337	2.02041854	up	1.13346E-06	0.000183634	AKR1E2
liver	rna1597	2.020730968	up	0.005996225	0.034547825	SEC63
liver	rna49589	2.023076462	up	0.006279503	0.035386115	GPR180
liver	rna53594	2.024821285	up	0.000539484	0.008820436	LOC110256111
liver	rna18259	2.026666551	up	0.001863161	0.01768514	RNF144A
liver	rna50834	2.027115447	up	3.78095E-09	2.99472E-06	LOC100626206
liver	rna1386	2.027842988	up	0.000770128	0.01061868	BACH2
liver	rna18813	2.029708716	up	0.009196472	0.044687832	LOC106509939
liver	rna49890	2.029950627	up	0.000129992	0.003633919	ATP11A
liver	rna40431	2.030098129	up	0.002726588	0.02166832	LOC106507788
liver	rna72678	2.034085732	up	7.3532E-07	0.000141668	CHN2
liver	rna61431	2.035819762	up	0.007168415	0.03827719	SUSD2
liver	rna46041	2.040606094	up	0.005147446	0.031510152	LOC110255523
liver	rna2675	2.041886305	up	8.5023E-05	0.002737181	MAPK6
liver	rna15018	2.047104364	up	9.93432E-07	0.000170643	LOC106509702
liver	rna53922	2.052492395	up	0.009260337	0.044875091	COL6A6
liver	rna69645	2.052644332	up	0.001051738	0.012718086	LOC110257410
liver	rna40287	2.053950325	up	0.000514297	0.008616132	LOC102161749
liver	rna67079	2.054722361	up	1.78054E-06	0.000246458	ASIC4
liver	rna48739	2.055395676	up	0.004560376	0.029287066	LOC110255743
liver	rna52373	2.057150951	up	0.002986216	0.022791478	ACACA
liver	rna39043	2.058520335	up	0.001571435	0.016141924	WDR25
liver	rna28259	2.05919627	up	0.000429039	0.007665183	FXYD7
liver	rna12714	2.059510668	up	0.000160128	0.004227683	KLHL3
liver	rna3073	2.059600239	up	0.009649657	0.045934944	GANC
liver	rna53723	2.06149323	up	0.001716142	0.016932202	LOC102157481
liver	rna63924	2.061586219	up	2.72936E-07	7.07499E-05	ABLIM1
liver	rna12894	2.066336502	up	0.000474086	0.008173358	SLC4A9
liver	gene21777	2.068671443	up	7.94235E-12	1.88723E-08	LOC102159217
liver	rna16944	2.069732878	up	0.007365908	0.038748115	SERTAD2
liver	rna46544	2.071626632	up	0.005399993	0.032318624	LOC110255621
liver	rna31755	2.072304951	up	0.00197411	0.0183588	TAF12
liver	rna77948	2.074272381	up	0.003217209	0.02372879	LOC110258800
liver	rna42145	2.075242791	up	5.95103E-05	0.002203735	DNHD1
liver	rna75724	2.0755658	up	0.008006915	0.040784063	FGF13
liver	rna44893	2.07556599	up	0.010127778	0.047264068	ASB4
liver	rna7764	2.076081544	up	0.000147955	0.004011534	LOC100519643
liver	rna17478	2.079891691	up	0.0060016	0.034547825	THUMPD2
liver	rna70957	2.081371104	up	0.006591384	0.036406999	FAM209B

tissue	transcript_id	log2FoldChange	regulation	pvalue	padj	gene_sympo
liver	rna29074	2.082770817	up	0.001492866	0.015672891	SNRPD2
liver	rna28632	2.085076546	up	9.82436E-06	0.000769593	DLL3
liver	rna16288	2.085843766	up	0.002185372	0.019268306	LOC102163181
liver	rna10851	2.086802445	up	0.000365924	0.007012065	DOHH
liver	rna42067	2.093497387	up	0.009109458	0.044325439	LOC110262320
liver	rna36611	2.093543502	up	0.000169101	0.004391393	ELOVL5
liver	rna60843	2.093633066	up	8.36259E-06	0.000706117	TPCN1
liver	rna22267	2.09447554	up	0.001176092	0.013511316	LOC110260354
liver	rna25975	2.095043878	up	0.006305572	0.035448949	MICAL3
liver	rna1688	2.096745599	up	0.000369502	0.007042615	FIG4
liver	rna56043	2.099862259	up	0.000223538	0.005156921	ITPR1
liver	rna33507	2.101756694	up	2.93239E-07	7.46554E-05	FGGY
liver	rna19710	2.102876364	up	3.13591E-06	0.000369493	PAG1
liver	rna17905	2.10422408	up	0.00981038	0.046463388	DNMT3A
liver	rna32677	2.10473322	up	2.28617E-06	0.000301796	TTC39C
liver	rna26764	2.107224444	up	1.49439E-07	4.26109E-05	SYT1
liver	rna61542	2.108930933	up	0.001889428	0.01787497	LRRC74B
liver	gene17637	2.110431508	up	0.003574631	0.025255359	LOC102165183
liver	rna10720	2.110663899	up	0.000696314	0.010058111	DPP9-AS1
liver	rna53737	2.116148497	up	0.001630903	0.016531585	LOC110256117
liver	rna42076	2.117454592	up	0.004284867	0.028190749	PPFIBP2
liver	rna15589	2.11773528	up	0.001694822	0.016825862	UNKL
liver	gene4337	2.118431446	up	0.009481593	0.045438343	LOC110259384
liver	rna34886	2.11930399	up	0.010025651	0.04697793	KIAA0319
liver	rna67980	2.120110119	up	2.44431E-05	0.001320022	GHR
liver	rna1680	2.121147161	up	7.47742E-05	0.002520227	ZBTB24
liver	rna29335	2.121285259	up	0.001616862	0.016455999	LOC110261018
liver	rna46709	2.122142412	up	0.007789422	0.040062695	MARC1
liver	gene17894	2.124060567	up	0.001631475	0.016531585	LOC100514001
liver	rna39172	2.124212616	up	7.13016E-05	0.002455428	FGFRL1
liver	rna52908	2.125399617	up	0.000386287	0.007227411	SERPINF1
liver	rna10785	2.126270532	up	0.000722761	0.010263356	ZBTB7A
liver	rna49472	2.12647227	up	0.001783241	0.017239403	LOC102158131
liver	rna1127	2.127254355	up	0.000680089	0.009934456	RSPH4A
liver	gene27916	2.129742939	up	0.002558195	0.020939717	LOC100524542
liver	rna39155	2.130349735	up	0.003029998	0.022929237	TMEM175
liver	rna40352	2.130775681	up	0.007194769	0.038303143	LOC110262115
liver	rna40944	2.139767708	up	0.000200309	0.004870946	LOC110262151
liver	rna33651	2.13976832	up	0.004589062	0.029391851	TRNAK-UUU
liver	rna6431	2.14278567	up	0.002393401	0.020299063	NUP214
liver	rna670	2.143760093	up	0.004732326	0.029972799	MAP3K5
liver	rna15506	2.147168946	up	0.002993778	0.022812554	NUBP2
liver	rna29482	2.148540222	up	0.007201871	0.038326641	VRK3
liver	rna68827	2.151192005	up	0.006138446	0.034997969	LOC110257243
liver	rna21921	2.152895637	up	0.00980854	0.046463388	VTCN1
liver	gene18635	2.153564311	up	2.44121E-05	0.001320022	LOC106505335
liver	rna48632	2.155701923	up	0.007765092	0.040024195	CDX2

tissue	transcript_id	log2FoldChange	regulation	pvalue	padj	gene_sympo
liver	rna30074	2.157038828	up	0.002689439	0.021596622	SSC5D
liver	rna47444	2.15787968	up	2.59235E-09	2.17407E-06	FAM205A
liver	gene15318	2.161038453	up	9.23657E-05	0.002881527	LOC110262127
liver	rna37588	2.164255171	up	0.000246354	0.005470816	LOC110261592
liver	rna55273	2.164523777	up	3.36429E-05	0.001609553	ACY1
liver	rna36111	2.164778777	up	0.000299247	0.006154193	DNAH8
liver	rna67510	2.16516765	up	0.000347385	0.006821856	PRLH
liver	rna2853	2.167363115	up	0.001661926	0.016650795	SQOR
liver	rna25959	2.170271364	up	0.001351869	0.014770503	BID
liver	rna14642	2.173929326	up	0.005161538	0.031547836	LOC110260108
liver	rna29639	2.174880823	up	0.00399449	0.027080096	CEACAM18
liver	rna40335	2.179111066	up	0.008160621	0.041285358	LOC100525933
liver	rna9487	2.180352184	up	1.67097E-05	0.001075598	RPL18A
liver	rna53673	2.184240307	up	0.000526317	0.008698373	CENPV
liver	rna18241	2.185796118	up	0.005770868	0.033774739	LOC106509914
liver	rna46247	2.186346508	up	4.87219E-08	1.7811E-05	LOC106507881
liver	rna46227	2.186368076	up	5.89539E-05	0.002194533	LOC106507881
liver	rna61582	2.18662858	up	0.002098565	0.01889289	TSSK2
liver	rna3824	2.188580304	up	0.006498018	0.036041922	LOC110261941
liver	rna48040	2.191740957	up	0.002702418	0.021596622	NEBL
liver	rna196	2.192754053	up	0.000138802	0.003820275	SLC22A3
liver	rna53838	2.194271714	up	0.000145474	0.00396562	RAI1
liver	rna10700	2.195980166	up	0.000103917	0.00313223	KDM4B
liver	rna53671	2.196369188	up	0.003486286	0.024916136	UBB
liver	rna53593	2.202887728	up	0.002240079	0.019499173	LOC110256111
liver	rna276	2.203604969	up	0.000775545	0.010663343	ZDHHC14
liver	rna75729	2.209351187	up	0.00035561	0.006907266	LOC110257819
liver	rna18998	2.210210857	up	0.005030937	0.03118009	TATDN1
liver	rna56988	2.211116632	up	0.000416135	0.007509914	LOC102162136
liver	rna9682	2.214709067	up	3.8951E-06	0.000433848	CYP4F55
liver	rna38752	2.216475507	up	0.002568664	0.020986061	EFCAB11
liver	gene26611	2.21753379	up	0.000188247	0.004712849	LOC110257525
liver	rna70189	2.217736706	up	0.000202289	0.004888381	MYH7B
liver	rna74284	2.218040634	up	1.44839E-06	0.000215061	PFKFB1
liver	rna26955	2.219883871	up	0.003401152	0.024576901	MVD
liver	rna70511	2.221623789	up	7.04942E-05	0.002443056	SGK2
liver	rna4636	2.224116441	up	0.004675298	0.029743739	IFNA1
liver	rna64304	2.22440983	up	0.000622553	0.009422223	LOC106506157
liver	rna67959	2.224445447	up	0.002978379	0.022776431	MROH2B
liver	rna23760	2.228356013	up	0.004313554	0.028298411	DNAJC22
liver	rna63457	2.228648866	up	5.98493E-05	0.002210547	LOC110256606
liver	rna53603	2.231329015	up	8.7274E-05	0.002761024	LOC110256111
liver	rna53197	2.231402758	up	0.000800195	0.010854782	GP1BA
liver	rna28752	2.232047109	up	0.004684543	0.029775985	LOC110260992
liver	rna75288	2.232340552	up	4.81277E-05	0.001983112	LOC102165589
liver	gene22633	2.23750781	up	0.000454731	0.007946012	LOC100739639
liver	rna24418	2.241728357	up	0.008051042	0.040893579	LOC100512656

tissue	transcript_id	log2FoldChange	regulation	pvalue	padj	gene_sympo
liver	rna36193	2.242210214	up	0.009894426	0.046627679	PGC
liver	rna14492	2.243898072	up	0.001719403	0.016939957	FAM57B
liver	rna31456	2.245301608	up	0.0004343	0.007730105	TCEA3
liver	rna34185	2.246200994	up	0.006751112	0.036905909	LOC100524382
liver	rna8883	2.246464762	up	0.001997795	0.018423396	LOC106507279
liver	rna64700	2.250829123	up	0.000893155	0.011531269	NCKAP5
liver	rna44283	2.254153488	up	0.001639957	0.01659434	SNX19
liver	rna48384	2.256064282	up	0.000908814	0.011651949	ADARB2
liver	rna7616	2.257526301	up	2.41244E-05	0.001317179	LOC110259242
liver	rna13234	2.257716678	up	2.98111E-05	0.00150715	LOC100514340
liver	rna23244	2.2588043	up	2.72374E-08	1.18405E-05	LOC110260835
liver	rna63948	2.262460315	up	1.60135E-05	0.001047269	ABLIM1
liver	rna39286	2.269156841	up	0.002476947	0.020687655	SH3BP2
liver	rna6834	2.271019501	up	0.000445627	0.007862996	MUC6
liver	rna71046	2.274669564	up	0.000222019	0.005130189	STX16
liver	rna65178	2.282211295	up	0.000206465	0.004972237	ACSL1
liver	rna67977	2.282894107	up	4.77312E-07	0.000107025	GHR
liver	rna57709	2.285019627	up	0.007447498	0.039079491	LOC110256384
liver	rna15014	2.28514347	up	0.002853877	0.022282432	PMM2
liver	rna7617	2.287708213	up	5.80108E-07	0.000118151	LOC110255291
liver	rna73028	2.289717981	up	0.00024919	0.005499546	LOC110257518
liver	rna70182	2.289998644	up	0.002132389	0.019061908	ACSS2
liver	gene17604	2.291686482	up	0.002774342	0.02191065	LOC106505091
liver	rna19207	2.293618241	up	0.005604959	0.033185174	LOC110260239
liver	rna68547	2.298908644	up	0.007773414	0.040038137	LOC102164818
liver	rna63597	2.300059636	up	0.000950424	0.011959569	PSD
liver	rna46080	2.305436277	up	0.003162599	0.023483945	NMNAT2
liver	rna70330	2.306112102	up	0.003551599	0.025197051	SLA2
liver	rna19054	2.30860053	up	0.004167042	0.02768384	LOC102162152
liver	gene18575	2.309808757	up	1.6874E-05	0.001078803	LOC100514422
liver	rna71936	2.31014635	up	0.006699009	0.036762037	LOC106506761
liver	rna11507	2.318368758	up	1.79422E-05	0.00113468	SFXN1
liver	gene23184	2.318693239	up	0.000883862	0.011497458	LOC100155384
liver	rna6939	2.321466817	up	0.002784957	0.021952864	TH
liver	rna8201	2.323586583	up	0.001091963	0.01294108	NR1H3
liver	rna75082	2.324294642	up	0.006917259	0.037438206	ACSL4
liver	rna25058	2.324630425	up	0.00066922	0.009866666	STK38L
liver	rna11973	2.326664493	up	1.36561E-05	0.000956634	PCSK1
liver	rna72677	2.327951722	up	8.60688E-05	0.002739023	CHN2
liver	rna7000	2.329095244	up	0.003699542	0.025666363	LOC102159152
liver	rna53597	2.334433464	up	0.000440969	0.007809816	LOC110256111
liver	gene10523	2.336042317	up	0.000625001	0.009449245	LOC110260970
liver	gene4666	2.336903937	up	0.00302769	0.022929237	LOC110259439
liver	rna5900	2.338966235	up	0.000317293	0.006407439	TTLL11
liver	rna33503	2.349077167	up	1.40107E-05	0.000960336	FGGY
liver	rna24821	2.356616042	up	4.11031E-05	0.001825566	LOC110260707
liver	rna32544	2.357974769	up	0.004916833	0.030685404	METTL4

tissue	transcript_id	log2FoldChange	regulation	pvalue	padj	gene_sympo
liver	rna25056	2.360459968	up	0.001166194	0.013430073	STK38L
liver	rna52175	2.364774668	up	3.35201E-05	0.001609078	LOC110256168
liver	rna53284	2.366379732	up	7.31006E-07	0.000141668	CLEC10A
liver	rna40336	2.373860426	up	0.003590908	0.025291946	LOC100738056
liver	rna53730	2.380662353	up	0.000141015	0.00385962	LOC110256119
liver	rna67709	2.387843862	up	0.000747357	0.010417739	FARP2
liver	gene28406	2.387904618	up	0.000336224	0.00668288	LOC110258065
liver	rna12623	2.388901133	up	0.002180243	0.019266345	JADE2
liver	rna48383	2.389133893	up	6.32625E-05	0.002294996	ADARB2
liver	rna66546	2.390432667	up	0.000699042	0.010066914	CFLAR
liver	rna50245	2.396637152	up	3.83922E-05	0.001748747	AATK
liver	rna33501	2.39687318	up	0.000366895	0.007021242	FGGY
liver	rna26554	2.401273983	up	2.54313E-09	2.17407E-06	LOC102157618
liver	rna73024	2.413525158	up	0.000778805	0.010663343	LOC106506868
liver	rna34070	2.416574231	up	0.002364729	0.020187988	ST3GAL3
liver	rna40268	2.419233127	up	0.000427758	0.007661498	HOPX
liver	rna59334	2.427560823	up	0.000524186	0.008698373	LOC102166913
liver	rna27716	2.428119696	up	9.54173E-09	6.47792E-06	LOC110260940
liver	rna36194	2.430997474	up	0.003515362	0.025046737	PGC
liver	rna7274	2.433426655	up	4.23711E-05	0.001847354	RBM4
liver	gene10173	2.433493287	up	0.000252374	0.005544058	LOC110261266
liver	rna56207	2.433691885	up	0.006561047	0.036270199	ATG7
liver	rna53315	2.444417748	up	0.000556309	0.008994576	PHF23
liver	rna52364	2.449935444	up	9.43568E-10	1.22295E-06	ACACA
liver	rna30070	2.453509181	up	0.000210505	0.005018685	ISOC2
liver	gene18484	2.461716468	up	0.000453869	0.007946012	LOC102167951
liver	gene26026	2.46572964	up	0.000442418	0.00782575	LOC100520157
liver	rna2266	2.469315464	up	0.000159134	0.004217062	ST8SIA3
liver	rna53721	2.470357019	up	1.18143E-05	0.000859369	LOC110256118
liver	rna61873	2.474209195	up	0.00050039	0.008442678	RAB4A
liver	rna6638	2.478118293	up	1.79868E-05	0.00113468	RXRA
liver	gene10385	2.478234532	up	3.04105E-06	0.000363395	LOC110261349
liver	rna27717	2.486865748	up	0.000521157	0.008669937	LOC110260940
liver	rna24339	2.488000757	up	0.001202664	0.013739092	PAN2
liver	rna8855	2.494959687	up	0.006251045	0.035351509	SOX6
liver	rna65179	2.495617621	up	0.000744255	0.0103926	ACSL1
liver	gene15207	2.49874959	up	0.002199464	0.019356644	LOC110262113
liver	rna74466	2.50344447	up	0.006420055	0.035782145	NLGN3
liver	rna15860	2.504716877	up	5.87759E-05	0.002193634	NINJ1
liver	rna13430	2.505374515	up	0.001141085	0.013215637	LOC106509613
liver	rna21861	2.506974677	up	2.20292E-05	0.001303196	LOC110260431
liver	rna50046	2.516212273	up	0.000224214	0.00516416	RAB40B
liver	rna75663	2.517389403	up	0.002423859	0.020472133	FHL1
liver	rna73025	2.520407333	up	0.000564554	0.009046821	LOC110257557
liver	rna43709	2.522682179	up	0.000825951	0.011077687	LOC110255440
liver	rna38223	2.532737194	up	0.000123592	0.003468596	LOC110261690
liver	rna65075	2.535269687	up	0.001156841	0.013354719	VEGFC

tissue	transcript_id	log2FoldChange	regulation	pvalue	padj	gene_sympo
liver	rna39876	2.538106464	up	6.43621E-06	0.000615846	LOC102166909
liver	rna46246	2.540048887	up	5.45852E-05	0.002091991	LOC106507881
liver	rna54046	2.540451234	up	0.000809816	0.010943651	THRB
liver	rna18608	2.541414005	up	0.000364154	0.006999683	ZNF623
liver	rna23570	2.544165227	up	0.000578801	0.009086934	LOC110260650
liver	rna78519	2.549838376	up	0.000732328	0.010303686	LOC102159510
liver	rna119	2.552507638	up	4.35775E-05	0.001871338	UNC93A
liver	rna17739	2.553256456	up	6.08837E-08	2.17005E-05	C3H2orf16
liver	rna26276	2.559191806	up	1.57862E-05	0.00103716	LOC110260785
liver	rna49874	2.562965391	up	0.001951885	0.018283853	TEX29
liver	rna40593	2.566158686	up	4.49654E-05	0.001897428	LOC110262129
liver	rna14766	2.569798266	up	0.001345192	0.014729955	UMOD
liver	rna47	2.578266206	up	0.001579567	0.016194354	WDR27
liver	gene27947	2.591144647	up	1.64464E-05	0.001070669	LOC110257801
liver	rna23485	2.592566198	up	0.000942666	0.011903981	GCAT
liver	rna26817	2.596042824	up	5.94209E-05	0.002203735	PRDM7
liver	rna1357	2.599729457	up	0.006005207	0.034550537	RRAGD
liver	rna71715	2.615386115	up	0.005387465	0.032287542	TRPV6
liver	rna57431	2.61951229	up	0.000178607	0.004571639	LOC100624963
liver	rna73027	2.62619877	up	0.001849818	0.017628914	BLVRA
liver	rna47812	2.629343827	up	0.002113751	0.018965224	DCLRE1C
liver	rna48725	2.630382419	up	0.00142006	0.015258878	LOC106505256
liver	rna14043	2.632368306	up	0.000588152	0.009098366	DTX2
liver	rna60733	2.636210216	up	6.664E-05	0.002369295	PEBP1
liver	rna14493	2.645272129	up	5.78824E-05	0.002165957	LOC110260083
liver	rna59255	2.652298773	up	0.008944364	0.043851373	LOC110256483
liver	rna38226	2.653732125	up	0.006832319	0.03715041	LOC110261689
liver	rna27230	2.658244783	up	0.002020334	0.018583159	LOC102159045
liver	rna60047	2.660408837	up	0.005417473	0.03234931	LOC110256746
liver	rna8083	2.666040456	up	0.002561717	0.02095376	C1QTNF4
liver	gene27772	2.676245657	up	0.001311573	0.01451793	LOC100517444
liver	rna48742	2.677947092	up	0.000684886	0.009953544	LOC106507937
liver	rna52354	2.686314713	up	0.000562285	0.00902758	MRM1
liver	rna67976	2.693946118	up	1.67805E-06	0.000236871	GHR
liver	rna23245	2.698208698	up	1.60395E-08	8.03293E-06	LOC110260623
liver	rna42042	2.700367014	up	0.006660086	0.036590695	LOC100736607
liver	rna48588	2.703720561	up	0.000194995	0.00479852	LOC102162170
liver	rna6593	2.711649941	up	0.002505764	0.020749987	DBH
liver	rna34120	2.714557475	up	1.46729E-06	0.000215061	SZT2
liver	rna45879	2.714973261	up	0.002043251	0.018661519	PAPPA2
liver	gene19635	2.721995518	up	3.5577E-07	8.7452E-05	LOC106507964
liver	rna65436	2.732306461	up	0.000116551	0.003341611	PPP1R3B
liver	gene8718	2.737784436	up	8.32727E-05	0.00271675	LOC100624537
liver	rna37014	2.739009658	up	0.005468823	0.032554909	LOC106507705
liver	rna28874	2.741104566	up	0.00981942	0.046479238	BSP1
liver	rna21920	2.748170456	up	3.62522E-06	0.000410197	LOC110260487
liver	rna62658	2.750209016	up	0.000193497	0.00479852	SFTPA1

tissue	transcript_id	log2FoldChange	regulation	pvalue	padj	gene_sympo
liver	rna73230	2.752967162	up	0.000983808	0.012175484	LOC110257805
liver	rna68437	2.757002437	up	0.003166855	0.02350331	LOC102159645
liver	rna67512	2.758732054	up	1.04992E-06	0.000174055	RAB17
liver	rna1971	2.762288478	up	0.00146246	0.015548313	SETBP1
liver	rna21952	2.763760144	up	0.004803438	0.030321303	LOC106510188
liver	rna31475	2.771073435	up	0.00155253	0.016016226	HMGCL
liver	rna40340	2.771362677	up	0.000528357	0.008698373	LOC100513671
liver	rna52669	2.771617544	up	0.001584647	0.016230111	LYRM9
liver	rna10010	2.787167742	up	0.002889409	0.022366119	LOC100521431
liver	rna16176	2.803524178	up	0.00829497	0.04176523	FHL2
liver	gene12479	2.807717855	up	0.007478893	0.039160932	LOC100512969
liver	rna7107	2.816966858	up	0.002555702	0.020939717	LRP5
liver	rna48738	2.82101771	up	0.00645586	0.035899139	LOC110255743
liver	rna9641	2.821171946	up	0.000293548	0.00609373	LOC110259618
liver	rna38791	2.823342321	up	0.000171843	0.004424679	C7H14orf159
liver	gene8446	2.824623844	up	0.000519708	0.008669937	LOC100515376
liver	rna65434	2.825324732	up	9.9268E-05	0.003047762	PPP1R3B
liver	rna1972	2.832700169	up	0.001078529	0.012856674	SETBP1
liver	rna44499	2.850794045	up	0.001007764	0.012396633	CNTN2
liver	rna67594	2.857838111	up	0.000456531	0.007949288	PER2
liver	rna34376	2.858008989	up	0.000461819	0.008009927	DUSP22
liver	rna6624	2.858897465	up	0.008630251	0.042737578	LOC102160511
liver	rna36666	2.858917239	up	0.001215069	0.01384589	LOC106507695
liver	gene6607	2.862186117	up	6.50312E-05	0.002327423	LOC110260126
liver	rna73545	2.866083169	up	0.000105312	0.003157955	PHKA2
liver	rna67720	2.873138653	up	0.000694	0.010045036	FARP2
liver	rna69015	2.887710747	up	5.98933E-06	0.000588895	LOC110257282
liver	gene9443	2.888169577	up	0.000797448	0.010838146	LOC110260732
liver	rna55299	2.894191446	up	1.06981E-12	3.81307E-09	DNAH1
liver	rna23185	2.89907241	up	1.69031E-08	8.03293E-06	LOC110260616
liver	rna39875	2.907944885	up	0.000342418	0.006742893	CORIN
liver	rna25855	2.917705348	up	3.00268E-05	0.001507365	IQSEC3
liver	rna15568	2.929371612	up	3.94695E-05	0.001784947	LOC106507362
liver	rna33494	2.951987198	up	3.05866E-06	0.000363395	FGGY
liver	rna38874	2.95526088	up	0.001492777	0.015672891	SERPINA6
liver	gene20080	2.95564374	up	0.005670734	0.033380537	LOC110255883
liver	gene27190	2.959303947	up	0.001233801	0.013971645	LOC100156370
liver	rna31022	2.968458903	up	4.73926E-06	0.0005005	MTHFR
liver	rna11764	2.972422446	up	0.000394029	0.007338545	LOC110259417
liver	rna36347	2.978079875	up	1.1941E-08	6.80972E-06	SLC22A7
liver	rna71702	2.98235828	up	0.000923627	0.011743719	LOC106508196
liver	rna28873	2.987602687	up	0.00052665	0.008698373	TEX101
liver	rna15857	2.989848598	up	0.001709582	0.016893024	CARD19
liver	rna10616	2.993210077	up	2.21606E-05	0.001304235	SLC25A41
liver	rna48269	2.995343763	up	7.79399E-10	1.11119E-06	ANKRD16
liver	rna69086	3.013970775	up	0.001294636	0.014425158	LOC110257296
liver	rna70088	3.016003068	up	7.96425E-05	0.002634486	LOC110257390

tissue	transcript_id	log2FoldChange	regulation	pvalue	padj	gene_sympo
liver	rna23098	3.021217638	up	0.002465273	0.020639232	LOC102159820
liver	rna33824	3.031509641	up	0.00092628	0.01175509	LOC106508570
liver	rna49592	3.039385014	up	3.68991E-05	0.00171119	SOX21
liver	rna46701	3.044619583	up	0.001655773	0.016650795	MARC2
liver	rna77730	3.04663497	up	0.006065054	0.03475461	LOC102166957
liver	rna60640	3.070692887	up	0.007624975	0.039675676	CUX2
liver	rna11080	3.099671438	up	0.000123109	0.00346186	CIRBP
liver	rna29331	3.099713887	up	0.000675955	0.009927772	BAX
liver	rna24208	3.101796664	up	2.07204E-06	0.000276085	LOC110260851
liver	rna67590	3.102762011	up	2.17311E-05	0.001296321	PER2
liver	rna21193	3.114597778	up	0.000100279	0.003063128	KCNN3
liver	rna24209	3.126025005	up	1.11058E-05	0.000829871	LOC110260576
liver	rna58089	3.128343824	up	0.004515467	0.029129872	MYLK
liver	rna61793	3.168116809	up	0.001886012	0.017854498	PCNX2
liver	rna59262	3.172817631	up	0.005522773	0.032807572	LOC106505859
liver	rna58055	3.197336906	up	0.002558532	0.020939717	MUC13
liver	rna13228	3.20496536	up	0.000379814	0.007143806	IL17B
liver	rna22260	3.210796923	up	1.28982E-08	7.07267E-06	LOC110260348
liver	rna55857	3.217080714	up	0.001732165	0.016967384	MAGI1
liver	rna1973	3.230718085	up	6.71775E-05	0.002376551	LOC110260756
liver	rna13185	3.234694308	up	0.000653599	0.009703535	SH3TC2
liver	rna9640	3.283993198	up	1.97736E-05	0.00123106	LOC100511184
liver	gene7953	3.303555615	up	7.15632E-05	0.002458497	LOC100156055
liver	rna69670	3.370134899	up	1.30607E-06	0.000198091	LOC106506630
liver	rna53893	3.410307188	up	0.000301024	0.006161479	KCNJ12
liver	rna71703	3.471808075	up	0.000104664	0.003148084	TAS2R40
liver	rna5532	3.518531809	up	0.005811529	0.033929145	LOC100153672
liver	rna22496	3.548468144	up	0.000763996	0.010554549	SNX7
liver	rna23097	3.554157541	up	0.00624918	0.035351509	LOC110260610
liver	rna42044	3.563613127	up	0.001074811	0.012833815	LOC100736745
liver	rna30121	3.574973668	up	3.03667E-05	0.001508497	LOC110261066
liver	rna27470	3.580861243	up	0.000253713	0.005556356	CHTF8
liver	rna49534	3.642920459	up	0.000105435	0.003157955	SLITRK1
liver	rna44897	3.67749302	up	0.003445422	0.024727864	ASB4
liver	rna52614	3.727808973	up	0.010271147	0.047590425	MIR193A
liver	rna36667	3.753098844	up	0.000352688	0.00687863	LOC100158035
liver	rna48251	3.763745005	up	8.41632E-07	0.000155833	PFKFB3
liver	rna75827	3.865171754	up	3.31879E-05	0.001603933	LOC110257797
liver	rna71709	3.884993649	up	0.000732166	0.010303686	KEL
liver	rna52754	3.907412643	up	1.40864E-05	0.000960911	LOC100515508
liver	rna69075	3.918582541	up	0.000356247	0.006910224	AHRR
liver	rna23186	3.958154501	up	1.52336E-09	1.72903E-06	PNPLA3
liver	gene27386	3.962111709	up	0.004373716	0.028561322	LOC110257678
liver	rna22088	3.986146597	up	0.000596523	0.009182785	TRNAE-UUC
liver	rna48250	3.991435033	up	4.59397E-05	0.001909511	PFKFB3
liver	rna71088	4.045918327	up	0.00038519	0.007216363	NELFCD
liver	rna38875	4.04708125	up	0.000338433	0.00668288	SERPINA6

tissue	transcript_id	log2FoldChange	regulation	pvalue	padj	gene_sympo
liver	rna55631	4.075603853	up	0.0004649	0.008043787	LOC110256254
liver	rna22898	4.102407861	up	2.25891E-05	0.001309155	LOC106510256
liver	rna44894	4.165500373	up	0.003000215	0.0228258	ASB4
liver	gene8205	4.175860057	up	0.001382339	0.015009906	LOC100621817
liver	rna38155	4.410397653	up	0.004045413	0.02721777	LOC110261688
liver	gene22263	4.441044951	up	0.000819487	0.011042937	LOC100157248
liver	gene21925	4.504455169	up	0.000111925	0.003256955	LOC110256482
liver	gene4041	4.519905211	up	0.001289945	0.014390254	LOC100737176
liver	gene1612	4.57300669	up	6.75776E-05	0.002384786	LOC100525628
liver	rna12137	4.596513584	up	0.003252777	0.023851178	LOC100626591
liver	rna34826	4.635958667	up	3.35172E-05	0.001609078	LOC106504333
liver	rna71708	4.644921735	up	4.42551E-05	0.001883416	LOC100621164
liver	rna5713	4.655321763	up	8.54984E-05	0.002739023	SAL1
liver	rna71706	4.735898617	up	0.000299872	0.006154193	LOC100621272
liver	gene10786	4.782412231	up	0.002500661	0.020735182	LOC106510548
liver	gene10348	4.78761596	up	0.001146273	0.013254189	LOC100511083
liver	rna59254	4.79074341	up	5.43799E-06	0.000561434	LOC110256481
liver	rna71707	4.857551188	up	0.000254283	0.005560292	LOC100621797
liver	rna71704	4.955064349	up	0.005649151	0.033357666	TAS2R39
liver	gene21928	4.966088202	up	7.25932E-06	0.000667717	LOC110256479
liver	rna71705	4.994030886	up	0.001450083	0.015462856	PIP
liver	rna59253	5.024471591	up	6.28569E-06	0.000609523	LOC110256480
liver	rna7614	5.033104324	up	5.64566E-05	0.002118163	LOC102164585
liver	gene6515	5.477196297	up	0.001403218	0.0151648	LOC102158023
liver	rna5531	5.616858441	up	7.28889E-05	0.002490275	LOC100520168
liver	rna62014	5.897344538	up	0.006035376	0.034666381	LOC106506012
liver	rna48249	5.921575212	up	2.95045E-06	0.000356479	PFKFB3
liver	rna1123	6.229757059	up	0.006341738	0.035515566	ZUFSP
liver	rna19712	6.321403111	up	0.000444267	0.00784872	LOC102157450
liver	rna63198	6.462757907	up	0.000241344	0.005418645	LOC110255181
liver	rna53897	6.514153981	up	1.1961E-06	0.000191605	LOC110256151
liver	rna63195	6.576492936	up	0.000171773	0.004424679	CYP2C36
liver	gene6513	6.601813476	up	2.22297E-05	0.001304235	LOC110260069
liver	rna45324	6.79661028	up	0.003178734	0.023545145	LOC110255495
liver	rna50126	6.994339342	up	2.71407E-06	0.000335059	CBR2
liver	gene23294	7.326285717	up	0.000233971	0.005294811	LOC100620144
liver	rna33893	9.470051618	up	8.28305E-06	0.000706117	DMBX1
msln	rna10507	-4.534640081	down	1.08775E-06	0.001649382	RETN
msln	id768389	-4.279208407	down	1.1015E-06	0.001649382	LOC110256716
msln	id963301	-3.708480142	down	2.31295E-06	0.002664158	LOC110258396
msln	rna20826	-3.616874516	down	2.39742E-05	0.010914182	CD5L
msln	rna37662	-3.534083124	down	9.53123E-05	0.026429737	CEBPE
msln	id968206	-2.806754729	down	1.57802E-06	0.002148117	LOC110258832
msln	rna77978	-2.565176191	down	2.15785E-05	0.010770569	LOC110258825
msln	rna14092	-2.52094698	down	1.22784E-05	0.008389296	CCL26
msln	id963295	-2.427779662	down	6.32956E-05	0.020903565	LOC110258394
msln	rna74728	-2.424051838	down	9.94054E-05	0.027047833	LOC110257759

tissue	transcript_id	log2FoldChange	regulation	pvalue	padj	gene_sympo
msln	id966701	-2.420280957	down	2.54459E-07	0.000635046	LOC110258689
msln	id966746	-2.379601446	down	1.35082E-11	2.02272E-07	LOC110258705
msln	id963089	-2.354305432	down	1.28803E-05	0.008389296	LOC110255225
msln	id968227	-2.331910757	down	0.000180794	0.038566621	LOC110258831
msln	id966707	-2.303702925	down	2.08856E-05	0.010770569	LOC110258693
msln	rna52456	-2.295884671	down	0.000228884	0.045096112	LOC100516039
msln	rna60946	-2.210636287	down	0.00014497	0.033396521	OASL
msln	rna67327	-2.134632952	down	9.49901E-07	0.001649382	LOC110257029
msln	id966723	-2.117171833	down	2.40529E-05	0.010914182	LOC110258690
msln	id966578	-2.087999266	down	1.32891E-05	0.008389296	LOC110258666
msln	rna59367	-2.080073886	down	5.34693E-05	0.019528027	MX1
msln	rna77796	-2.054558214	down	4.70771E-05	0.018075175	LOC110258673
msln	id966565	-2.037242729	down	7.03403E-05	0.021495412	LOC110258664
msln	rna1018	2.075526332	up	3.00844E-05	0.012870983	LOC106508857
msln	rna32762	2.260385059	up	0.000160982	0.036523502	GAREM1
msln	rna22632	2.364381023	up	9.23029E-05	0.026297031	MTF2
msln	rna22885	2.706604504	up	9.56441E-06	0.007537763	CLCA1
msln	rna6837	3.038971912	up	5.08457E-06	0.004677582	MUC5B
msln	rna37047	3.499678618	up	0.000248402	0.048102296	CSPG4
msln	rna35770	3.84113739	up	0.000139493	0.032658985	KLHL31
msln	rna7718	4.697816521	up	1.55727E-05	0.008968672	PHEROC
msln	rna5713	5.096412221	up	3.15816E-11	2.36451E-07	SAL1
msln	rna7204	5.87269702	up	6.40178E-05	0.020903565	CLCF1
msln	rna7719	6.547679338	up	1.20607E-05	0.008389296	SCGB1D1
pbmc	rna14244	-7.064108841	down	6.94025E-05	0.001975351	CALN1
pbmc	rna42286	-6.691894337	down	0.000165319	0.004066494	LOC100515788
pbmc	rna42724	-6.170656889	down	0.000581856	0.010633689	LOC106504821
pbmc	rna34374	-5.865056505	down	0.001636474	0.022823422	DUSP22
pbmc	rna70883	-5.837861997	down	4.33681E-05	0.001343984	ZFP64
pbmc	rna33148	-5.647007409	down	0.004175509	0.044249124	MIGA1
pbmc	gene15477	-5.595079925	down	0.000191149	0.004576415	LOC100518205
pbmc	rna6333	-5.554248033	down	0.004698906	0.048433691	CRAT
pbmc	rna78308	-5.373475089	down	0.000716068	0.012435165	LOC110259044
pbmc	rna34200	-5.163559825	down	0.001069779	0.016549075	C6H1orf50
pbmc	rna34869	-5.073418395	down	7.95312E-06	0.000330072	HDGFL1
pbmc	rna48547	-5.058267991	down	0.00080074	0.01343072	AMER2
pbmc	rna49838	-4.936405621	down	0.000612393	0.011073608	LOC110255861
pbmc	rna67687	-4.898680813	down	0.001931995	0.025563656	HDLBP
pbmc	rna72320	-4.87116983	down	0.001171321	0.01784157	RNF133
pbmc	rna50942	-4.639906023	down	4.90545E-06	0.000217992	PITPNC1
pbmc	rna9705	-4.41674316	down	4.83044E-10	5.79714E-08	LOC110259330
pbmc	rna60785	-4.368399267	down	0.001988918	0.026151567	MED13L
pbmc	rna15562	-4.27734192	down	0.000688863	0.012096511	TELO2
pbmc	rna50086	-4.205443114	down	0.000785032	0.01327842	OGFOD3
pbmc	rna16299	-4.1858888	down	0.002643313	0.031967465	LOC102163417
pbmc	rna32088	-4.149236149	down	8.68918E-07	4.99151E-05	KIAA0319L
pbmc	rna23654	-4.148489553	down	2.12632E-05	0.000746072	PRDM4

tissue	transcript_id	log2FoldChange	regulation	pvalue	padj	gene_sympo
pbmc	rna67112	-4.137190918	down	1.31796E-05	0.000496659	LOC100626715
pbmc	rna52143	-4.130867354	down	0.000473884	0.009129774	LOC100621260
pbmc	rna67464	-4.110215633	down	3.54353E-06	0.000165675	LOC102164001
pbmc	rna42285	-4.098220662	down	2.43126E-09	2.48966E-07	HBB
pbmc	rna21208	-3.968613353	down	0.004235021	0.044669723	ATP8B2
pbmc	rna19325	-3.776965435	down	5.76555E-09	5.54102E-07	NCALD
pbmc	rna76131	-3.718360124	down	0.000137401	0.003488857	LOC100517800
pbmc	rna69292	-3.709065144	down	0.00052734	0.009887378	F11
pbmc	rna70000	-3.69519548	down	0.00020324	0.004810863	XKR7
pbmc	rna71147	-3.636469248	down	0.002268173	0.028603609	MTG2
pbmc	rna44360	-3.520525633	down	0.000773173	0.013148123	FMO3
pbmc	rna73106	-3.501851782	down	8.21163E-05	0.002275346	ZNF777
pbmc	rna32994	-3.494962007	down	0.000258259	0.005765164	SSX2IP
pbmc	rna3159	-3.49386877	down	0.00031256	0.006697258	BAHD1
pbmc	rna43787	-3.453344725	down	0.004357641	0.045640171	PDZD3
pbmc	rna74934	-3.315495292	down	0.001686956	0.02326913	TCEAL1
pbmc	rna24414	-3.304650184	down	0.002968021	0.034735062	SDR9C7
pbmc	rna53341	-3.297967269	down	4.81112E-06	0.000214305	TNK1
pbmc	rna78541	-3.292631439	down	0.001039394	0.016198724	LOC102158335
pbmc	rna50479	-3.279716193	down	1.31693E-05	0.000496659	RNF157
pbmc	rna30701	-3.239229334	down	5.42689E-09	5.2708E-07	PRDM16
pbmc	rna38830	-3.22976947	down	4.88898E-05	0.001486544	BTBD7
pbmc	rna30416	-3.198215985	down	0.002638153	0.031925548	ZNF329
pbmc	rna27356	-3.192745057	down	0.000739708	0.012775056	TAT
pbmc	rna31296	-3.174608149	down	9.11097E-09	8.29318E-07	HP1BP3
pbmc	rna26990	-3.115186848	down	6.21276E-05	0.001799961	FBXO31
pbmc	rna41357	-3.100480543	down	0.000470147	0.009079347	LEF1
pbmc	rna13077	-3.086180228	down	0.003107346	0.035854419	NR3C1
pbmc	rna27361	-3.076787485	down	0.001235149	0.018573561	PHLPP2
pbmc	rna11791	-3.072858355	down	0.001698497	0.023342875	RASA1
pbmc	rna33511	-3.054515367	down	0.003671606	0.04050375	LOC102165506
pbmc	rna5792	-3.028288263	down	0.003632943	0.04017131	TMEM268
pbmc	rna51294	-3.017931843	down	0.002848848	0.033653913	SLC4A1
pbmc	rna7333	-3.010742602	down	0.000337794	0.007064049	CNIH2
pbmc	rna20677	-2.998160211	down	0.002062707	0.026877958	CD84
pbmc	rna23060	-2.992841689	down	0.003687948	0.040588973	PANX2
pbmc	rna17123	-2.958150124	down	0.004013962	0.042899073	FANCL
pbmc	rna46080	-2.939836502	down	1.21964E-05	0.000469146	NMNAT2
pbmc	rna69343	-2.919557739	down	5.90313E-05	0.001724445	SMIM19
pbmc	id900368	-2.886832423	down	0.000684838	0.012037042	LOC102166836
pbmc	rna73079	-2.87588639	down	0.00068443	0.012037042	RALA
pbmc	rna72538	-2.852841007	down	0.002202877	0.028026067	DPY19L1
pbmc	rna64743	-2.82638461	down	0.000751095	0.012889014	LOC102163381
pbmc	rna64243	-2.825041318	down	0.000464489	0.009013285	DHX32
pbmc	rna38823	-2.804334611	down	0.001778741	0.024181129	ITPK1
pbmc	rna67858	-2.782112252	down	0.004728623	0.048671878	LOC110257218
pbmc	rna38890	-2.776806851	down	0.000214693	0.005006481	LOC106504547

tissue	transcript_id	log2FoldChange	regulation	pvalue	padj	gene_sympo
pbmc	rna59334	-2.768693527	down	0.001575841	0.022254736	LOC102166913
pbmc	rna74018	-2.76405075	down	0.000785852	0.013279848	FTSJ1
pbmc	rna70355	-2.751879992	down	8.0231E-05	0.002242898	SAMHD1
pbmc	rna28501	-2.749759466	down	0.000167377	0.004106413	LOC100739425
pbmc	rna17929	-2.713866098	down	0.001375476	0.020137315	NCOA1
pbmc	rna22475	-2.703375083	down	0.002939899	0.034484993	AGL
pbmc	rna45665	-2.699011976	down	0.002333873	0.02913383	CUL1
pbmc	rna19641	-2.697602741	down	0.001140949	0.017467596	LOC100154873
pbmc	rna10500	-2.691396176	down	0.003357044	0.037990048	TRAPPC5
pbmc	rna67709	-2.688057413	down	0.003013237	0.035133297	FARP2
pbmc	rna71257	-2.684230664	down	8.70044E-05	0.002379299	ARFRP1
pbmc	rna19880	-2.673737814	down	0.000997357	0.015723729	NCOA2
pbmc	rna7642	-2.672286013	down	0.003267864	0.037271848	SLC22A6
pbmc	rna39677	-2.66897613	down	0.000813992	0.013560773	DTHD1
pbmc	rna46652	-2.666968498	down	0.001521355	0.021654768	LYPLAL1
pbmc	rna15008	-2.65472787	down	9.95507E-05	0.002668185	USP7
pbmc	rna22731	-2.649054889	down	0.000409822	0.008197304	ZNF644
pbmc	rna10742	-2.646363953	down	0.000437202	0.008598912	UBXN6
pbmc	rna30775	-2.638979896	down	0.001845541	0.024802912	GPR153
pbmc	rna993	-2.634127741	down	4.93848E-05	0.001498405	LOC110256655
pbmc	rna2039	-2.6100632	down	4.30778E-08	3.38197E-06	ZBTB7C
pbmc	rna26820	-2.606623067	down	0.000280945	0.006141037	GAS8
pbmc	rna55767	-2.601362962	down	0.00325983	0.037247856	THOC7
pbmc	rna59415	-2.600522922	down	0.004818396	0.049341423	PDE9A
pbmc	rna31517	-2.592715642	down	2.03932E-05	0.000718223	NCMAP
pbmc	rna65915	-2.577100646	down	0.002653054	0.032044125	LOC106506279
pbmc	rna73930	-2.573554713	down	0.000812922	0.013554939	LOC110257673
pbmc	rna34907	-2.573087856	down	3.06513E-05	0.001025811	RIPOR2
pbmc	gene4613	-2.555423157	down	0.002334788	0.02913383	LOC110259426
pbmc	rna71187	-2.550677548	down	0.000373898	0.007674275	GID8
pbmc	rna5858	-2.542084665	down	0.00024024	0.0054146	PHF19
pbmc	rna15457	-2.538646218	down	0.001014083	0.015922792	SLC9A3R2
pbmc	rna7222	-2.52870855	down	0.000454495	0.008846682	GRK2
pbmc	rna45943	-2.516619362	down	0.002080311	0.027051219	RALGPS2
pbmc	rna6853	-2.516257573	down	0.000455258	0.008852401	MOB2
pbmc	rna42105	-2.509334621	down	7.39149E-05	0.002078664	ZNF215
pbmc	rna20056	-2.507305541	down	6.21895E-05	0.001799961	CHD7
pbmc	rna14673	-2.493171683	down	0.003479588	0.03896548	GGA2
pbmc	rna67579	-2.479465523	down	0.000166732	0.004095901	SCLY
pbmc	rna60477	-2.459547532	down	2.65036E-06	0.000129709	BCL7A
pbmc	rna39501	-2.451100813	down	0.000172888	0.004223089	TAPT1
pbmc	rna2658	-2.436687451	down	0.00095316	0.015181274	MYO5A
pbmc	rna5338	-2.428720486	down	0.001340108	0.019757686	GNE
pbmc	rna27557	-2.424884441	down	0.000569842	0.010461164	ADGRG1
pbmc	rna63134	-2.407760392	down	0.000385828	0.007862309	IDE
pbmc	rna16899	-2.396813565	down	0.002401314	0.029786415	C1D
pbmc	rna48490	-2.394547837	down	0.000119947	0.003112994	LATS2

tissue	transcript_id	log2FoldChange	regulation	pvalue	padj	gene_sympo
pbmc	rna8749	-2.386006147	down	0.004454173	0.046342088	SAA2
pbmc	rna34864	-2.371877915	down	8.62031E-06	0.000352329	LOC110261655
pbmc	rna53662	-2.354053193	down	0.000392741	0.007965578	ZNF624
pbmc	rna64423	-2.342196186	down	0.000101497	0.002708794	LOC100156553
pbmc	rna14239	-2.340474639	down	0.000232722	0.005308659	AUTS2
pbmc	id462455	-2.33246655	down	0.003026037	0.035221423	LOC102162433
pbmc	rna42160	-2.33167095	down	1.69552E-05	0.000617931	APBB1
pbmc	rna72673	-2.331010873	down	0.00208776	0.027129366	PRR15
pbmc	rna78560	-2.328483164	down	0.001730771	0.023714825	DIP2A
pbmc	rna23255	-2.327094098	down	0.00046517	0.009017223	SEPT3
pbmc	rna25449	-2.326103603	down	0.002811249	0.033398205	TP23
pbmc	rna29547	-2.322692114	down	0.001775096	0.024166443	SYT3
pbmc	rna9191	-2.31631852	down	0.001551541	0.021997088	TRIM58
pbmc	rna15811	-2.309620784	down	0.000767856	0.013081325	ZNF484
pbmc	rna65408	-2.304748462	down	9.73985E-05	0.002621689	SMIM18
pbmc	rna34137	-2.295195434	down	0.000183604	0.004460015	ELOVL1
pbmc	rna45550	-2.293118032	down	0.0021229	0.027375097	HBP1
pbmc	rna73643	-2.260803573	down	0.00025333	0.005662221	ZFX
pbmc	rna67789	-2.257258485	down	3.05591E-07	1.97868E-05	RETREG1
pbmc	rna7184	-2.235229244	down	3.36422E-05	0.001102412	GPR152
pbmc	rna51026	-2.226765652	down	0.001250973	0.018756291	LIMD2
pbmc	rna25004	-2.221297629	down	0.004077028	0.043449867	CCDC91
pbmc	rna21793	-2.212114918	down	4.27646E-07	2.66811E-05	LOC102166500
pbmc	rna48138	-2.210732627	down	5.18198E-06	0.000228662	CAMK1D
pbmc	rna25286	-2.207193444	down	2.65124E-05	0.000904976	AEBP2
pbmc	rna51307	-2.206385141	down	0.000709932	0.012364004	UBTF
pbmc	rna77977	-2.205219641	down	0.003507229	0.039172029	LOC110258824
pbmc	rna55547	-2.199440877	down	0.000718789	0.01247093	DENND6A
pbmc	rna16493	-2.189711407	down	0.00081917	0.013614113	KDM3A
pbmc	rna65632	-2.187893158	down	0.00147728	0.021183344	LOC110256997
pbmc	rna19337	-2.187522449	down	5.41265E-05	0.001598512	LOC110260427
pbmc	rna35256	-2.184786216	down	0.002220406	0.028164122	TRIM10
pbmc	rna39292	-2.182739791	down	4.80891E-05	0.001473324	LOC110262043
pbmc	rna40278	-2.177111465	down	0.004302759	0.045165781	HOPX
pbmc	rna5675	-2.176348002	down	0.001778387	0.024181129	KIAA0368
pbmc	rna16295	-2.174794995	down	0.001423887	0.02065349	CHST10
pbmc	rna28028	-2.172098277	down	0.00158098	0.022297029	ADCY7
pbmc	rna41359	-2.164896023	down	0.000112878	0.002958063	LEF1
pbmc	rna22467	-2.150135483	down	0.001160342	0.017702962	SLC35A3
pbmc	rna1724	-2.130820069	down	0.00190431	0.025357602	RPF2
pbmc	rna37494	-2.126897164	down	0.001277264	0.01905479	LOC106504460
pbmc	rna16636	-2.117824869	down	9.4882E-05	0.002564945	LBX2
pbmc	rna24706	-2.116416151	down	0.000623406	0.011229656	LOC102166619
pbmc	rna19326	-2.113816957	down	0.000391589	0.00795077	NCALD
pbmc	rna23071	-2.10682807	down	0.001930345	0.025559781	ALG12
pbmc	rna55519	-2.100744449	down	0.000320498	0.006831259	LOC110256250
pbmc	rna3928	-2.090957836	down	0.001115687	0.01713266	LARP6

tissue	transcript_id	log2FoldChange	regulation	pvalue	padj	gene_sympo
pbmc	rna24104	-2.07844924	down	4.13462E-07	2.58819E-05	SMUG1
pbmc	rna74061	-2.074617202	down	0.00067328	0.011900512	PQBP1
pbmc	rna34670	-2.074417491	down	0.002751659	0.032855996	HIVEP1
pbmc	rna35897	-2.072769232	down	0.001861336	0.024961778	LOC110261506
pbmc	rna22474	-2.063788906	down	0.000128645	0.003315901	AGL
pbmc	rna15970	-2.062365061	down	0.001992139	0.02617565	ANAPC1
pbmc	rna32030	-2.061753991	down	0.000963179	0.015263423	CSMD2
pbmc	rna7990	-2.052807612	down	0.000299877	0.006479688	ZDHHC5
pbmc	rna69087	-2.050487478	down	2.25263E-05	0.00078455	PLEKHG4B
pbmc	rna58039	-2.039414128	down	0.000222187	0.005155718	SNX4
pbmc	rna34292	-2.037973552	down	0.000347112	0.007202962	SLFNL1
pbmc	rna11566	-2.03368213	down	0.002456379	0.030309818	GCNT4
pbmc	rna78575	-2.031580966	down	4.5503E-05	0.00140552	COL6A2
pbmc	rna44254	-2.031236339	down	0.004331359	0.045397379	ST14
pbmc	rna58387	-2.030554951	down	0.000557202	0.010313163	LOC110256422
pbmc	rna11507	-2.028927101	down	0.003895981	0.04211593	SFXN1
pbmc	rna35258	-2.021670789	down	0.002440543	0.030153911	TRIM15
pbmc	rna77351	-2.018084588	down	0.001150558	0.017582161	TEX22
pbmc	rna6864	-2.017831474	down	0.000109658	0.002889766	SYT8
pbmc	rna10507	2.004381235	up	0.001695641	0.02332064	RETN
pbmc	rna59971	2.00623032	up	0.00014137	0.003570632	LOC106508068
pbmc	rna61770	2.009391452	up	3.8902E-10	4.79079E-08	LOC102158191
pbmc	rna34205	2.014139812	up	1.54269E-07	1.08867E-05	CLDN19
pbmc	rna10944	2.024897529	up	0.001970246	0.025996758	C2H19orf35
pbmc	rna56300	2.02637916	up	1.07852E-08	9.45185E-07	SLC6A6
pbmc	rna34843	2.02667405	up	0.00054435	0.010165157	MBOAT1
pbmc	rna35439	2.027216704	up	7.85911E-06	0.00032689	TNF
pbmc	rna30517	2.028384853	up	5.64229E-13	1.13098E-10	LOC110261384
pbmc	rna16104	2.028409494	up	0.000284101	0.006188476	CCDC138
pbmc	rna7663	2.028850776	up	4.14711E-10	5.07402E-08	NXF1
pbmc	rna25500	2.038775479	up	0.000757507	0.012975405	OLR1
pbmc	rna24213	2.045904982	up	0.003985631	0.042739189	ITGA7
pbmc	rna35888	2.048912624	up	1.23314E-06	6.73471E-05	HMGA1
pbmc	rna8742	2.053635906	up	0.000259099	0.005770616	LDHA
pbmc	rna64165	2.054731783	up	0.000529743	0.00992189	LOC102167106
pbmc	rna23750	2.056002179	up	0.001980847	0.026081846	LOC100127131
pbmc	rna46041	2.064337775	up	2.44398E-07	1.64463E-05	LOC110255523
pbmc	rna50469	2.064404761	up	3.44053E-06	0.000162066	SPHK1
pbmc	rna51645	2.064553308	up	2.37808E-06	0.000118539	NR1D1
pbmc	rna23975	2.077520739	up	3.94693E-12	6.69982E-10	RARG
pbmc	rna22097	2.085180752	up	1.5536E-07	1.09227E-05	SLC16A1
pbmc	rna21673	2.092958287	up	9.73429E-11	1.33878E-08	LOC110260327
pbmc	rna49850	2.094297017	up	3.03308E-13	6.42128E-11	ING1
pbmc	rna37662	2.094890371	up	0.000281849	0.006153656	CEBPE
pbmc	rna5468	2.095305358	up	1.22901E-06	6.73167E-05	NR4A3
pbmc	rna12894	2.100615025	up	1.11484E-06	6.1782E-05	SLC4A9
pbmc	rna52758	2.1101832	up	5.54194E-11	7.91071E-09	CRYBA1

tissue	transcript_id	log2FoldChange	regulation	pvalue	padj	gene_sympo
pbmc	rna28626	2.115572189	up	1.78219E-14	4.66388E-12	TRNAI-UAU
pbmc	rna70500	2.129556431	up	1.00784E-18	5.27493E-16	L3MBTL1
pbmc	rna17330	2.129612451	up	9.71273E-15	2.69128E-12	STPG4
pbmc	rna52911	2.131538813	up	0.001840272	0.024767437	RTN4RL1
pbmc	rna50579	2.141533152	up	1.60702E-06	8.55351E-05	LOC102165522
pbmc	rna28374	2.141966272	up	3.43882E-14	8.75598E-12	NFKBID
pbmc	rna12195	2.141999658	up	6.8175E-09	6.48764E-07	LOC110259442
pbmc	rna31109	2.146978462	up	1.02873E-08	9.12616E-07	CTRC
pbmc	rna21362	2.148957025	up	0.000869346	0.014194297	S100A12
pbmc	rna53053	2.153058434	up	0.000806724	0.013487392	CAMKK1
pbmc	rna72447	2.153183048	up	2.58853E-10	3.26191E-08	LOC106506809
pbmc	rna11603	2.154245619	up	0.002282278	0.028668453	CRHBP
pbmc	gene91	2.159633829	up	1.89947E-07	1.31098E-05	LOC102158869
pbmc	rna36822	2.163596157	up	2.62315E-06	0.000129386	FES
pbmc	rna39336	2.165297752	up	4.0244E-06	0.000184219	HTRA3
pbmc	rna7134	2.165926285	up	2.01671E-08	1.63788E-06	TCIRG1
pbmc	rna57585	2.166713874	up	0.000125062	0.003232396	EPHB3
pbmc	rna61054	2.169183648	up	0.000694499	0.012172786	CMKLR1
pbmc	rna13288	2.173336345	up	6.5233E-05	0.00187366	CAMK2A
pbmc	gene22224	2.177629519	up	0.000191927	0.004589205	LOC100623855
pbmc	rna36048	2.18281763	up	5.47644E-14	1.35773E-11	LOC102161912
pbmc	rna29977	2.185827903	up	1.00409E-05	0.000397948	NLRP2
pbmc	rna14343	2.188935069	up	2.13672E-10	2.79583E-08	TRIM72
pbmc	rna56484	2.196644003	up	1.11627E-05	0.000435462	RAB6B
pbmc	rna5690	2.197991583	up	3.11572E-08	2.49814E-06	C1H9orf84
pbmc	rna28372	2.198228626	up	1.76025E-05	0.00063427	NFKBID
pbmc	rna38115	2.202104134	up	2.83164E-06	0.000137156	HSPA2
pbmc	rna13341	2.202780593	up	1.50753E-05	0.000563937	LOC110259820
pbmc	rna51252	2.209980178	up	0.001567436	0.022172401	HIGD1B
pbmc	rna37365	2.211334899	up	0.002099778	0.027238041	LOC110261576
pbmc	rna7723	2.218016525	up	6.45547E-14	1.53967E-11	BEST1
pbmc	rna4394	2.222061061	up	3.30926E-06	0.000156666	DAAM1
pbmc	rna64161	2.222881979	up	9.76699E-06	0.000390665	CHST15
pbmc	rna38202	2.225975314	up	0.001766374	0.024117403	ARG2
pbmc	rna51145	2.228681594	up	1.28288E-13	2.98419E-11	ARF2
pbmc	rna9431	2.229908406	up	5.33874E-17	2.23539E-14	KIAA1683
pbmc	rna75091	2.234428486	up	0.001769152	0.024120379	TMEM164
pbmc	rna16613	2.238104634	up	4.45993E-12	7.43664E-10	HK2
pbmc	rna52	2.240684026	up	0.000435104	0.008566587	THBS2
pbmc	rna63527	2.243264116	up	7.8016E-08	5.76462E-06	PDZD7
pbmc	rna41481	2.250207639	up	1.50442E-07	1.06967E-05	DNAJB14
pbmc	rna64162	2.252250129	up	5.14783E-07	3.13901E-05	CHST15
pbmc	rna59161	2.254718521	up	0.000580098	0.010611853	OLIG2
pbmc	rna15937	2.255113723	up	0.001055614	0.016370271	IL1B
pbmc	rna8522	2.262883013	up	0.00136852	0.020082291	LOC110259281
pbmc	rna36111	2.263498322	up	0.003100559	0.035818964	DNAH8
pbmc	rna24879	2.265418986	up	1.63304E-14	4.33376E-12	PHLDA1

tissue	transcript_id	log2FoldChange	regulation	pvalue	padj	gene_sympo
pbmc	rna72350	2.271079815	up	0.00257235	0.031290004	TSPAN12
pbmc	rna17410	2.271854862	up	2.52071E-14	6.50621E-12	LOC106509841
pbmc	rna24006	2.273708351	up	0.000942671	0.015052371	PCBP2
pbmc	rna27067	2.293414583	up	8.15554E-09	7.49594E-07	TLDC1
pbmc	rna50449	2.297605315	up	0.000986247	0.015563547	RHBDF2
pbmc	rna22426	2.298589703	up	3.32483E-09	3.32805E-07	EXTL2
pbmc	rna77204	2.300259741	up	1.37046E-06	7.44153E-05	TNFAIP2
pbmc	rna16778	2.301427699	up	1.92896E-05	0.000685762	LOC110259992
pbmc	rna24502	2.304006971	up	7.20874E-14	1.69784E-11	MARS
pbmc	rna24121	2.304379685	up	1.52963E-11	2.3432E-09	NFE2
pbmc	rna51553	2.311535293	up	1.4363E-09	1.53765E-07	GAST
pbmc	rna18966	2.312442738	up	0.000301292	0.006501901	LOC102166687
pbmc	rna33945	2.31632682	up	1.89788E-26	2.38399E-23	MMACHC
pbmc	rna4892	2.318074526	up	0.004440623	0.046277771	JAK2
pbmc	rna6341	2.321361771	up	2.9614E-16	1.0528E-13	IER5L
pbmc	rna65962	2.321656507	up	1.0972E-07	7.95132E-06	LOC110257023
pbmc	rna20	2.323304006	up	2.03138E-05	0.000716764	LOC110259814
pbmc	rna7551	2.331130571	up	0.003331205	0.037765679	FKBP2
pbmc	rna23887	2.3364279	up	0.000241902	0.005426098	ANKRD33
pbmc	rna69405	2.337473396	up	5.20039E-05	0.001547957	TMEM230
pbmc	rna46044	2.33863056	up	7.61428E-12	1.22622E-09	LOC110255525
pbmc	rna34728	2.3427043	up	1.57723E-10	2.09284E-08	LOC106507125
pbmc	rna13966	2.343029552	up	0.004419023	0.046231667	ACHE
pbmc	rna15422	2.346889739	up	3.82047E-10	4.73588E-08	RAB26
pbmc	gene14418	2.347550786	up	1.46886E-06	7.95297E-05	LOC110261694
pbmc	rna76749	2.350096005	up	0.00087565	0.014272497	SOHLH1
pbmc	rna27600	2.351664166	up	7.8795E-12	1.25818E-09	CFAP20
pbmc	rna63049	2.352461113	up	2.59998E-06	0.000128579	PANK1
pbmc	rna24504	2.354725897	up	6.08119E-17	2.49091E-14	DDIT3
pbmc	rna69408	2.367629839	up	2.75167E-13	5.8917E-11	TMEM230
pbmc	rna851	2.370229588	up	0.000306915	0.006593956	EPB41L2
pbmc	rna19450	2.373319714	up	1.25618E-05	0.000478161	LOC110260253
pbmc	rna23894	2.375248815	up	0.00035592	0.007345287	LOC110260567
pbmc	rna33917	2.378877074	up	0.000134123	0.003419677	LOC100511937
pbmc	rna34140	2.382310015	up	0.000324321	0.006873858	CDC20
pbmc	rna10943	2.386837326	up	2.25236E-19	1.28603E-16	LINGO3
pbmc	rna38201	2.391114404	up	1.0078E-08	8.99953E-07	ARG2
pbmc	rna55180	2.392746426	up	0.002125982	0.027380553	MAPKAPK3
pbmc	rna449	2.394860468	up	0.000527225	0.009887378	SASH1
pbmc	rna31584	2.402251165	up	8.35767E-09	7.64442E-07	STMN1
pbmc	rna33268	2.409986467	up	2.70974E-29	4.25474E-26	GADD45A
pbmc	rna15477	2.411127057	up	2.29843E-10	2.98008E-08	RPL3L
pbmc	rna22253	2.412665532	up	1.97836E-15	6.2127E-13	GSTM3
pbmc	rna56299	2.415420887	up	1.38556E-12	2.53463E-10	SLC6A6
pbmc	rna70843	2.418280881	up	0.000223448	0.005166755	LOC102165584
pbmc	rna60297	2.419641383	up	0.000476353	0.009167976	RFLNA
pbmc	rna29173	2.43011595	up	0.000260359	0.005791846	BBC3

tissue	transcript_id	log2FoldChange	regulation	pvalue	padj	gene_sympo
pbmc	rna24003	2.436107687	up	6.07091E-07	3.65457E-05	PRR13
pbmc	rna16107	2.447673633	up	4.87513E-05	0.001486544	CCDC138
pbmc	rna36412	2.450924124	up	9.89562E-27	1.33181E-23	LOC106504420
pbmc	rna64159	2.451587512	up	1.1917E-08	1.03E-06	CHST15
pbmc	rna9959	2.460639337	up	1.77547E-07	1.23444E-05	RTBDN
pbmc	rna207	2.462148563	up	0.000233536	0.005320786	LOC102166961
pbmc	rna31993	2.463976584	up	1.48816E-08	1.23524E-06	RNF19B
pbmc	rna50866	2.476396405	up	0.000354613	0.007326333	FAM20A
pbmc	rna31279	2.480618334	up	0.00190978	0.025379719	FAM43B
pbmc	rna7060	2.493122726	up	1.92094E-08	1.57367E-06	LOC106509289
pbmc	rna50245	2.495608475	up	2.63574E-07	1.74868E-05	AATK
pbmc	rna68014	2.500153362	up	5.51256E-20	3.58164E-17	HMGCS1
pbmc	rna29171	2.502033768	up	0.003744373	0.041008169	BBC3
pbmc	rna41972	2.505051351	up	0.001622673	0.022698145	LOC102167132
pbmc	rna55913	2.510733209	up	0.003169145	0.036410382	MITF
pbmc	rna62013	2.518228116	up	0.004020449	0.042944045	LOC106506012
pbmc	rna52531	2.520296755	up	9.97571E-05	0.002669921	CCL2
pbmc	rna10708	2.521945158	up	8.17331E-05	0.002269562	PLIN3
pbmc	rna56043	2.523174353	up	3.00573E-07	1.9529E-05	ITPR1
pbmc	rna25622	2.530307875	up	7.09792E-08	5.30711E-06	PTPN6
pbmc	rna61401	2.538705462	up	0.002336737	0.029138847	SPECC1L
pbmc	rna660	2.543955174	up	1.19117E-06	6.54344E-05	IL22RA2
pbmc	rna27511	2.551220624	up	0.000836137	0.013795534	NLRC5
pbmc	rna21428	2.555984508	up	3.15966E-09	3.18366E-07	S100A10
pbmc	rna59434	2.557152399	up	8.57251E-18	3.93959E-15	LOC110256500
pbmc	rna75206	2.561122496	up	0.000187919	0.00453643	ZCCHC12
pbmc	rna62882	2.570584302	up	0.001038347	0.016195801	RASSF4
pbmc	rna59727	2.573172517	up	0.000236325	0.005367024	EGR3
pbmc	rna5592	2.577552975	up	0.001589742	0.022370369	KLF4
pbmc	rna53897	2.579256401	up	0.000526487	0.009887378	LOC110256151
pbmc	rna33892	2.579714386	up	0.002709146	0.032513207	LOC110261236
pbmc	rna75912	2.583849241	up	0.002490339	0.030550326	MAMLD1
pbmc	rna52634	2.589207999	up	0.002658084	0.03205939	KSR1
pbmc	rna58123	2.590998791	up	0.000611832	0.011073608	HSPBAP1
pbmc	gene23871	2.593350431	up	0.003485348	0.03898156	LOC102164509
pbmc	rna77148	2.594431508	up	0.000998067	0.015723729	LOC100736929
pbmc	rna13527	2.594869825	up	0.002221202	0.028164122	FBXL18
pbmc	rna72448	2.595917621	up	4.02813E-06	0.000184219	LOC106506809
pbmc	rna72700	2.598191193	up	1.07939E-06	6.03362E-05	CREB5
pbmc	rna35343	2.601498101	up	0.002544141	0.031087356	FLOT1
pbmc	rna33074	2.605453305	up	0.00056521	0.010400081	ADGRL2
pbmc	rna66683	2.60738901	up	0.000190556	0.004568003	NRP2
pbmc	rna42838	2.608420563	up	0.000500108	0.009508617	SYTL2
pbmc	rna19408	2.620908655	up	0.003762769	0.041148055	OSR2
pbmc	rna23453	2.624430358	up	5.74915E-14	1.40682E-11	MAFF
pbmc	rna66069	2.625456575	up	0.00011732	0.003061683	DFNB59
pbmc	rna38536	2.627288523	up	1.56833E-11	2.3831E-09	LOC110261632

tissue	transcript_id	log2FoldChange	regulation	pvalue	padj	gene_sympo
pbmc	rna230	2.630334354	up	0.000707036	0.012335164	LOC106508799
pbmc	rna32198	2.630882362	up	1.93331E-13	4.18706E-11	POU3F1
pbmc	rna15938	2.637932076	up	6.57107E-06	0.000279486	IL1B
pbmc	rna41607	2.646413263	up	9.88411E-12	1.53914E-09	CCSER1
pbmc	rna43696	2.652694624	up	0.002435683	0.030113609	PHLDB1
pbmc	rna51756	2.6567866	up	0.002474233	0.030450363	FBXO47
pbmc	rna61238	2.66456081	up	0.001447767	0.020901478	LOC110256683
pbmc	rna67048	2.677825235	up	1.27905E-33	4.01665E-30	STK16
pbmc	rna77695	2.687230783	up	1.79319E-07	1.24218E-05	LOC102157946
pbmc	rna11658	2.69968716	up	4.50125E-08	3.51919E-06	HOMER1
pbmc	rna31664	2.703327858	up	6.30914E-27	9.14437E-24	LOC110261121
pbmc	rna28629	2.704775796	up	4.8915E-05	0.001486544	IL-15L
pbmc	gene13142	2.711997343	up	0.000262416	0.005830705	LOC100513549
pbmc	rna54536	2.718421064	up	1.31484E-05	0.000496659	LOC106505585
pbmc	rna41375	2.725506108	up	6.47106E-06	0.00027648	SGMS2
pbmc	rna6963	2.733268538	up	6.41328E-12	1.04172E-09	CDKN1C
pbmc	rna42872	2.739063819	up	2.36443E-05	0.000818945	CCDC83
pbmc	rna2416	2.744290202	up	5.83033E-08	4.42964E-06	C2CD4B
pbmc	rna76076	2.756673511	up	6.7466E-05	0.001927726	FLNA
pbmc	rna26626	2.760379412	up	8.62912E-20	5.41967E-17	LOC106510436
pbmc	rna42662	2.76244925	up	0.002469636	0.030413644	WNT11
pbmc	rna15089	2.769294347	up	0.000132308	0.003396386	ANKS3
pbmc	rna29327	2.776982525	up	1.29816E-16	5.09582E-14	TULP2
pbmc	rna11591	2.779393278	up	8.66365E-06	0.000352756	SV2C
pbmc	rna23720	2.77953793	up	3.55839E-46	6.70471E-42	RND1
pbmc	rna64160	2.788637455	up	6.69818E-15	1.94165E-12	CHST15
pbmc	rna29158	2.799011227	up	8.63235E-13	1.67681E-10	SLC1A5
pbmc	rna50727	2.808430177	up	9.40885E-06	0.000381251	CPSF4L
pbmc	rna76530	2.815195266	up	2.92998E-06	0.000141556	LOC106507771
pbmc	rna25624	2.816560114	up	1.85722E-06	9.65008E-05	LOC110260750
pbmc	rna11657	2.833959065	up	1.90002E-09	1.96704E-07	HOMER1
pbmc	rna53374	2.835527262	up	6.48597E-16	2.10705E-13	ATP1B2
pbmc	rna41768	2.838413976	up	0.000231612	0.005296166	NKX6-1
pbmc	rna24127	2.850504025	up	0.001219252	0.018393237	GPR84
pbmc	rna59688	2.855589754	up	1.08358E-06	6.03362E-05	HR
pbmc	rna70501	2.857223081	up	1.85595E-13	4.06625E-11	LOC106506015
pbmc	rna35347	2.871661579	up	2.32555E-17	1.04329E-14	IER3
pbmc	rna49788	2.874893052	up	9.97605E-09	8.95089E-07	EFNB2
pbmc	rna18039	2.878233471	up	3.67992E-05	0.001179201	SDC1
pbmc	rna8994	2.888775009	up	2.10033E-25	2.32791E-22	ADM
pbmc	rna71538	2.894586526	up	4.0429E-07	2.5477E-05	CRYGN
pbmc	rna68224	2.898674721	up	1.11588E-33	4.01665E-30	PLK2
pbmc	rna208	2.924421388	up	0.002246425	0.02844566	LOC102166961
pbmc	rna11228	2.925831236	up	2.24872E-06	0.000114206	GFPT2
pbmc	rna12203	2.929390221	up	0.000278943	0.006104353	LOC110259443
pbmc	rna76534	2.944425579	up	7.00568E-44	6.60005E-40	FAM166A
pbmc	rna77949	2.947436223	up	0.001602571	0.022483732	LOC110258801

tissue	transcript_id	log2FoldChange	regulation	pvalue	padj	gene_sympo
pbmc	rna2443	2.953433607	up	5.15631E-05	0.001547056	LOC110260996
pbmc	rna36809	2.962493712	up	9.9164E-06	0.000395021	FURIN
pbmc	rna10606	2.962955686	up	1.94055E-25	2.28524E-22	TNFSF9
pbmc	rna55947	2.970304246	up	4.11257E-05	0.001287194	LOC110256273
pbmc	rna49310	2.970440218	up	1.61567E-05	0.000592265	PCDH17
pbmc	rna75266	2.975984908	up	1.46576E-09	1.56033E-07	LOC100154946
pbmc	rna53748	2.980754769	up	0.001032134	0.016138977	LOC110256121
pbmc	rna29335	2.983297042	up	1.78522E-22	1.46248E-19	LOC110261018
pbmc	rna21430	2.98824782	up	0.000499347	0.009503725	LOC110260323
pbmc	rna30779	2.991637308	up	1.723E-06	9.06839E-05	HES2
pbmc	rna18146	2.994225808	up	1.92742E-05	0.000685762	C3H2orf50
pbmc	rna59777	3.011002639	up	0.000982911	0.015523892	EBF2
pbmc	rna51379	3.020804297	up	1.36024E-12	2.53463E-10	ARL4D
pbmc	rna3123	3.02306475	up	5.12793E-12	8.40178E-10	DLL4
pbmc	rna21037	3.023408183	up	0.000566086	0.010406049	ARHGEF2
pbmc	rna13315	3.024980689	up	0.002313197	0.029018147	LOC110259545
pbmc	rna46310	3.04056637	up	2.7371E-12	4.91166E-10	ATF3
pbmc	rna51034	3.041580506	up	0.000638175	0.011397631	KCNH6
pbmc	rna74034	3.043197026	up	3.84678E-07	2.44044E-05	WDR13
pbmc	rna48651	3.043630704	up	0.000303919	0.006537043	FLT1
pbmc	rna33916	3.045982888	up	0.000885025	0.014338469	LOC100511937
pbmc	rna28178	3.046483464	up	0.000605908	0.010987991	CHST8
pbmc	rna7724	3.053177703	up	0.000763247	0.013050001	RAB3IL1
pbmc	gene12981	3.05750043	up	6.28283E-15	1.87107E-12	LOC110261484
pbmc	gene3249	3.058225231	up	0.000519926	0.009816077	LOC110259293
pbmc	rna60425	3.070048308	up	2.0062E-20	1.45388E-17	HCAR2
pbmc	rna48309	3.072939243	up	0.000148538	0.003731672	LOC102165195
pbmc	rna70731	3.082162007	up	2.47896E-05	0.000857038	SULF2
pbmc	rna25633	3.097919932	up	2.21753E-16	8.19267E-14	LRRC23
pbmc	rna46111	3.101529821	up	3.32968E-05	0.001092993	COLGALT2
pbmc	rna16092	3.104077888	up	4.52314E-12	7.47588E-10	ASTL
pbmc	rna69952	3.111395784	up	4.37625E-33	1.03072E-29	ID1
pbmc	rna64548	3.111691653	up	1.79208E-05	0.000643167	LOC106504203
pbmc	rna77147	3.119796299	up	8.12864E-10	9.04173E-08	LOC100736929
pbmc	rna48652	3.121232943	up	2.37708E-06	0.000118539	FLT1
pbmc	rna22254	3.127354081	up	1.96944E-07	1.34939E-05	LOC102165301
pbmc	rna71174	3.170850446	up	7.58155E-19	4.08147E-16	SLCO4A1
pbmc	rna23278	3.173595582	up	1.08221E-10	1.4565E-08	CCDC134
pbmc	rna18040	3.181933596	up	0.000421703	0.008363926	LOC110260147
pbmc	rna68575	3.194987409	up	7.0053E-08	5.25872E-06	TRNAE-UUC
pbmc	rna63144	3.19894391	up	0.000858767	0.014045902	CYP26A1
pbmc	rna67368	3.200078369	up	0.000602053	0.010928592	NPPC
pbmc	rna59970	3.208389282	up	7.67895E-07	4.49338E-05	LOC102158040
pbmc	rna15298	3.248101776	up	8.58038E-05	0.002351393	CLDN6
pbmc	rna13618	3.249734046	up	9.85182E-12	1.53914E-09	NPTX2
pbmc	rna25576	3.253302231	up	0.000854693	0.01400359	NANOG
pbmc	rna72446	3.273658625	up	7.1129E-21	5.36085E-18	LSMEM1

tissue	transcript_id	log2FoldChange	regulation	pvalue	padj	gene_sympo
pbmc	rna70959	3.274557932	up	0.000424577	0.008403238	TFAP2C
pbmc	rna36415	3.277118727	up	1.22896E-29	2.1051E-26	TRNAV-AAC
pbmc	rna61055	3.278899627	up	8.15084E-05	0.00226851	CMKLR1
pbmc	rna61234	3.281504954	up	1.57404E-09	1.66618E-07	LIF
pbmc	rna36414	3.282637384	up	2.98611E-20	2.02807E-17	VEGFA
pbmc	rna14815	3.284689458	up	1.13746E-06	6.27223E-05	RPS15A
pbmc	rna31734	3.306967374	up	0.000102435	0.002729704	RCC1
pbmc	rna34827	3.308979891	up	7.95541E-05	0.002227279	ID4
pbmc	rna39315	3.345484588	up	0.001368091	0.020082291	LOC102163357
pbmc	rna52910	3.354826389	up	7.43118E-09	6.9316E-07	RTN4RL1
pbmc	rna36702	3.372990445	up	1.11355E-08	9.66886E-07	CEMIP
pbmc	rna17664	3.394311465	up	0.000337454	0.007064049	MEMO1
pbmc	rna59726	3.428124538	up	0.001679417	0.023216117	EGR3
pbmc	rna57586	3.429480584	up	1.31928E-12	2.48579E-10	EPHB3
pbmc	gene10910	3.440945227	up	1.33706E-16	5.14142E-14	LOC110261356
pbmc	rna31835	3.462662398	up	1.49682E-13	3.39796E-11	MATN1
pbmc	rna46306	3.466369176	up	4.71461E-23	4.03785E-20	FAM71A
pbmc	rna18689	3.474541938	up	6.35538E-15	1.87107E-12	ARC
pbmc	rna18091	3.475763273	up	2.74755E-16	9.95565E-14	FAM84A
pbmc	rna61233	3.482258842	up	9.65536E-11	1.33769E-08	LIF
pbmc	rna31660	3.508778864	up	2.96783E-07	1.94166E-05	FAM46B
pbmc	rna19275	3.533916545	up	6.13481E-06	0.000263909	DCSTAMP
pbmc	rna70827	3.546076891	up	3.01379E-20	2.02807E-17	LOC110257431
pbmc	rna38116	3.554459829	up	3.86376E-12	6.61827E-10	PPP1R36
pbmc	rna46033	3.573893487	up	1.76557E-05	0.000634864	LOC110255522
pbmc	rna70844	3.575131872	up	0.000239381	0.005410901	LOC110257432
pbmc	rna25464	3.662276294	up	2.8274E-07	1.86272E-05	STYK1
pbmc	rna61235	3.690197961	up	3.18447E-08	2.54245E-06	LIF
pbmc	rna30886	3.759490663	up	8.30098E-08	6.10965E-06	SPSB1
pbmc	rna21008	3.765452135	up	5.66953E-05	0.001663945	SEMA4A
pbmc	rna17324	3.793254852	up	9.66417E-07	5.46824E-05	LOC110260018
pbmc	rna29328	3.842329271	up	1.76055E-05	0.00063427	NUCB1
pbmc	rna52912	3.853993792	up	7.3037E-07	4.31399E-05	DPH1
pbmc	rna56235	3.886209053	up	0.000489745	0.009368305	PPARG
pbmc	rna33946	3.899074983	up	4.61626E-36	2.89932E-32	LOC110261410
pbmc	rna27509	3.97878929	up	2.51494E-10	3.20179E-08	LOC110261425
pbmc	rna38117	3.999818139	up	9.99244E-11	1.36433E-08	PPP1R36
pbmc	rna75076	4.03365167	up	9.62447E-06	0.000386662	KCNE5
pbmc	rna77948	4.06661673	up	6.98113E-09	6.60997E-07	LOC110258800
pbmc	rna6340	4.078499881	up	2.23408E-19	1.28603E-16	LOC106509274
pbmc	gene6791	4.11741683	up	1.26777E-18	6.45603E-16	LOC100523548
pbmc	rna56279	4.15488522	up	5.90177E-06	0.000254465	TRH
pbmc	rna6858	4.170878709	up	9.96392E-07	5.62096E-05	LOC110259170
pbmc	rna15976	4.171182822	up	1.24668E-05	0.000477437	LOC106509733
pbmc	rna49926	4.197917216	up	4.47564E-13	9.26704E-11	LOC110255782
pbmc	rna41030	4.256076416	up	7.07753E-07	4.19355E-05	PCDH10
pbmc	rna72821	4.268459341	up	3.11592E-05	0.001037283	LOC110257621

tissue	transcript_id	log2FoldChange	regulation	pvalue	padj	gene_sympo
pbmc	rna27707	4.279589501	up	1.25468E-05	0.000478161	CDH16
pbmc	rna76533	4.299530403	up	6.03667E-11	8.5521E-09	STPG3
pbmc	rna6857	4.309063628	up	1.77096E-06	9.29482E-05	IFITM10
pbmc	rna28367	4.336487384	up	5.91547E-05	0.001725377	KIRREL2
pbmc	rna72445	4.385179111	up	4.06998E-12	6.84702E-10	LOC100519118
pbmc	rna47961	4.669991849	up	4.27056E-06	0.000192964	LOC100738624
pbmc	rna1870	4.692074042	up	0.000317504	0.006775088	FAM46A
pbmc	rna77149	4.712891975	up	8.78543E-10	9.68041E-08	LOC100736929
pbmc	rna69409	5.12517807	up	7.48081E-09	6.94352E-07	TMEM230
pbmc	rna261	5.333451621	up	7.42805E-10	8.3808E-08	LOC106506437
pbmc	rna5085	5.562295253	up	1.67823E-11	2.5297E-09	LOC110257038
pbmc	rna48353	5.643673499	up	1.41952E-18	7.03858E-16	LOC102157904
smln	rna76978	-3.88495192	down	9.30499E-06	0.005510976	LOC110258215
smln	rna22939	-3.679102557	down	0.000894934	0.026604358	MAPK8IP2
smln	rna33960	-3.59139037	down	3.66909E-05	0.009098641	HPDL
smln	rna75884	-3.56981702	down	0.000692595	0.024389555	LOC100515119
smln	id968245	-3.549652883	down	0.000495899	0.020765556	LOC110258833
smln	id963044	-3.501155575	down	9.3379E-05	0.011854848	LOC110258347
smln	id963050	-3.475415439	down	3.91331E-05	0.009098641	LOC110258349
smln	rna26937	-3.268915927	down	0.000106469	0.011960369	CDT1
smln	rna77972	-3.249528976	down	0.000122232	0.012526585	LOC100038328
smln	rna72200	-3.247104066	down	0.002019195	0.038295863	IMPDH1
smln	rna30516	-3.152692668	down	8.63995E-05	0.011854848	ISG15
smln	id968234	-3.146405658	down	6.80519E-06	0.004416948	LOC110258829
smln	rna53611	-3.097505347	down	0.000504138	0.02081217	HS3ST3A1
smln	id968227	-3.045152554	down	0.002004149	0.038130929	LOC110258831
smln	rna75180	-3.012825255	down	0.002952138	0.046357303	LOC110257707
smln	rna28998	-2.999595871	down	6.497E-05	0.01054228	ZNF296
smln	rna70849	-2.957156169	down	0.000301406	0.018340252	BCAS4
smln	rna77978	-2.953021894	down	0.000602877	0.022942717	LOC110258825
smln	rna56428	-2.927605078	down	0.003301158	0.049005635	MCM2
smln	rna9475	-2.875058172	down	0.000228159	0.016115088	ARRDC2
smln	rna77971	-2.861639577	down	0.001990601	0.038062505	LOC100125542
smln	id968209	-2.858569076	down	3.41935E-05	0.009098641	LOC110258818
smln	rna34965	-2.855225406	down	5.57197E-05	0.009940323	LOC574051
smln	rna69033	-2.832250082	down	0.002059099	0.038657499	TERT
smln	rna9564	-2.824000899	down	0.002385551	0.041613181	ANKLE1
smln	rna23266	-2.731900576	down	0.000226657	0.016115088	SHISA8
smln	rna34980	-2.708470742	down	0.000803182	0.024956326	LOC595122
smln	rna50330	-2.705323559	down	0.003133516	0.048201741	SOCS3
smln	rna40284	-2.691199493	down	0.000127152	0.012806208	LOC100621006
smln	rna77975	-2.689105156	down	0.002103459	0.039256733	LOC110258822
smln	rna77796	-2.670770294	down	0.000316765	0.018794589	LOC110258673
smln	rna46013	-2.656765136	down	0.000370021	0.019213141	IER5
smln	rna10745	-2.6432056	down	0.000437921	0.019969645	CHAF1A
smln	rna49656	-2.622108891	down	0.001012481	0.02739915	STK24
smln	id968206	-2.620165837	down	0.00256239	0.042931365	LOC110258832

tissue	transcript_id	log2FoldChange	regulation	pvalue	padj	gene_sympo
smln	rna71847	-2.619643622	down	0.002490529	0.04223952	PARP12
smln	rna50348	-2.600703054	down	0.000297227	0.0182895	TK1
smln	rna7720	-2.589540597	down	0.00128556	0.030159041	INCENP
smln	rna71177	-2.573324128	down	0.000719163	0.024578759	OGFR
smln	rna23102	-2.552628574	down	0.001700404	0.034597104	GTSE1
smln	rna53344	-2.535236284	down	0.001600761	0.033879876	PLSCR3
smln	rna51958	-2.534027176	down	0.001062061	0.027686213	PPP1R9B
smln	rna51779	-2.52466362	down	0.000418468	0.019772346	EPOP
smln	rna7776	-2.4872659	down	3.26422E-06	0.002933533	TKFC
smln	rna59476	-2.482525736	down	0.000184738	0.015345828	ICOSLG
smln	rna67789	-2.47225153	down	0.001917795	0.036972936	RETREG1
smln	rna53792	-2.466335741	down	0.00255828	0.042931365	TOP3A
smln	rna70972	-2.451950185	down	0.00060729	0.023020786	RBM38
smln	rna56723	-2.41866349	down	0.003270756	0.048942821	CHST2
smln	rna34242	-2.380261189	down	0.000421408	0.019772346	HIVEP3
smln	rna46305	-2.377365444	down	0.002359458	0.041514379	BATF3
smln	rna29223	-2.341498391	down	0.000659568	0.023856755	LIG1
smln	rna27297	-2.323301683	down	0.00019454	0.015345828	EXOSC6
smln	rna7207	-2.323173189	down	0.000357484	0.018952106	POLD4
smln	rna68254	-2.279845912	down	0.000675983	0.024091272	PDE4D
smln	rna36009	-2.278673276	down	0.002846944	0.045813839	SRPK1
smln	rna61291	-2.262093374	down	0.002099893	0.039256733	OSBP2
smln	rna29439	-2.25683338	down	3.94271E-05	0.009098641	ADM5
smln	rna33828	-2.255532166	down	0.000853915	0.025979914	FOXD2
smln	rna63347	-2.242976048	down	0.000415172	0.019772346	FRAT1
smln	rna35498	-2.230617904	down	5.8787E-05	0.010020664	VARS
smln	rna62307	-2.202173327	down	0.000376233	0.019363555	DDIT4
smln	rna49839	-2.199495291	down	0.001645182	0.034128717	RAB20
smln	rna31871	-2.155605589	down	0.001244155	0.029534526	NKAIN1
smln	rna23073	-2.145559301	down	0.000279383	0.0182895	ZBED4
smln	rna76531	-2.144808785	down	0.001467053	0.032096591	TOR4A
smln	rna34470	-2.12973833	down	0.00132952	0.030627951	LOC110261697
smln	rna35971	-2.118197461	down	0.00222876	0.039946947	DEF6
smln	rna50295	-2.107912336	down	0.000121953	0.012526585	CBX4
smln	rna39741	-2.100577097	down	0.003372597	0.049562327	PDS5A
smln	rna18619	-2.09086893	down	0.000738675	0.024625132	PYCR3
smln	rna30065	-2.077647199	down	0.000231319	0.016115088	UBE2S
smln	rna29206	-2.066032268	down	0.000781811	0.024753124	NOP53
smln	rna71824	-2.055176521	down	0.002007235	0.038130929	ADCK2
smln	rna6188	-2.049214427	down	0.000297315	0.0182895	NAIF1
smln	rna15333	-2.034887714	down	0.000697198	0.024460547	PAQR4
smln	rna59367	-2.028457058	down	0.002921002	0.046054067	MX1
smln	rna50185	-2.012080982	down	0.002605482	0.043238416	ARHGDIA
smln	rna63349	-2.003682982	down	0.000448918	0.020068633	RRP12
smln	rna69117	-2.001524274	down	0.002891156	0.045908964	PRAG1
smln	rna60025	-2.001177379	down	0.000179625	0.015097576	LOC106507243
smln	rna41141	2.000180749	up	0.00268066	0.044091442	FGF2

tissue	transcript_id	log2FoldChange	regulation	pvalue	padj	gene_sympo
smln	rna40876	2.014786425	up	0.001420906	0.031499905	SMAD1
smln	rna22124	2.01551797	up	0.002994284	0.046830275	ST7L
smln	rna47539	2.022317928	up	0.000192574	0.015345828	DDX58
smln	rna28485	2.022518222	up	5.17542E-05	0.009940323	LOC110261285
smln	rna42922	2.024316339	up	0.00067075	0.024085815	LOC106504840
smln	gene18497	2.044629415	up	0.003428617	0.049821553	LOC110255821
smln	rna61229	2.090585362	up	0.001886342	0.036547479	MTMR3
smln	rna77557	2.09255647	up	0.000290096	0.0182895	FUT10
smln	rna25173	2.101475291	up	0.003385383	0.049594835	SOX5
smln	rna66668	2.113976265	up	0.002604722	0.043238416	RAPH1
smln	gene22891	2.119371937	up	0.002378454	0.041598016	LOC110256650
smln	rna16864	2.129981777	up	0.002447625	0.042176409	ANTXR1
smln	rna68573	2.133433389	up	0.001005583	0.02738515	DUSP1
smln	rna69282	2.134541773	up	0.001017458	0.02739915	FAT1
smln	rna43855	2.135058586	up	0.000291971	0.0182895	ARHGEF12
smln	rna8940	2.13536163	up	0.0020446	0.038589768	PARVA
smln	rna42633	2.161792131	up	0.000733964	0.024625132	KLHL35
smln	rna63866	2.170927777	up	0.001014318	0.02739915	LOC102165730
smln	rna41671	2.172696052	up	0.002910228	0.045946201	PKD2
smln	rna23220	2.190349578	up	0.003072876	0.047487312	ARFGAP3
smln	rna47166	2.190690439	up	0.003235664	0.048810899	LMOD1
smln	rna47949	2.194889336	up	0.001633921	0.033966365	ARHGAP21
smln	rna62562	2.196372137	up	0.001724374	0.034833097	LRMDA
smln	rna47542	2.198244595	up	0.000475659	0.02054026	DDX58
smln	rna47953	2.203555642	up	0.002817622	0.04546723	LOC110255586
smln	rna46358	2.207984316	up	0.000778885	0.024753124	HHAT
smln	rna43371	2.224650002	up	0.00118548	0.028794106	LOC110255432
smln	rna47306	2.227512352	up	0.000755811	0.024660685	LOC102158497
smln	rna22134	2.240827938	up	0.001075004	0.027724656	CTTNBP2NL
smln	rna62559	2.249730121	up	0.000188544	0.015345828	LRMDA
smln	rna3243	2.252206493	up	0.002795752	0.045239288	LOC106506784
smln	rna9019	2.258953968	up	0.001615154	0.033882403	WNT9A
smln	rna76971	2.262576699	up	0.003407739	0.049765762	LOC110258202
smln	rna1191	2.264656689	up	0.00342575	0.049821553	LOC106508872
smln	rna33576	2.267722511	up	0.001810002	0.035600461	PLPP3
smln	rna64700	2.278328994	up	0.001040339	0.027686213	NCKAP5
smln	rna71529	2.283100299	up	0.003112108	0.047966702	PRKAG2
smln	rna38717	2.286947413	up	0.001232491	0.029500651	PTPN21
smln	rna69156	2.293991342	up	0.00098183	0.02738515	DLC1
smln	rna25168	2.297421634	up	0.003177258	0.048333212	SOX5
smln	rna24574	2.297587121	up	0.001227977	0.02945884	LRIG3
smln	rna1192	2.309238652	up	0.001789511	0.035375387	LOC102157778
smln	rna34451	2.311752308	up	0.001002521	0.02738515	NQO2
smln	rna451	2.314971964	up	0.001078743	0.027759804	SASH1
smln	rna39568	2.324368678	up	0.000478212	0.02054026	SLIT2
smln	rna13319	2.346076289	up	0.002789356	0.045239288	SMIM3
smln	rna65639	2.351765001	up	0.00138134	0.031271919	TANC1

tissue	transcript_id	log2FoldChange	regulation	pvalue	padj	gene_sympo
smln	rna40306	2.352393867	up	0.003378822	0.049591428	ADGRL3
smln	rna67834	2.354387388	up	0.003152112	0.048201741	PDZD2
smln	rna7443	2.35451071	up	0.000718555	0.024578759	LOC102163816
smln	rna72659	2.355818231	up	0.002202296	0.039946947	MTURN
smln	rna67792	2.357159797	up	0.001143883	0.028245861	MYO10
smln	rna15632	2.359681553	up	0.000292314	0.0182895	LOC106509720
smln	rna56710	2.382748946	up	0.000248234	0.016959748	TRPC1
smln	rna73058	2.384831809	up	0.000481885	0.02062168	GLI3
smln	rna47044	2.390184787	up	0.000350752	0.018884544	LOC106505107
smln	rna47292	2.390594135	up	0.003048561	0.047173964	C10H9orf3
smln	rna43370	2.392595681	up	0.002229678	0.039946947	SIK2
smln	rna19576	2.396551918	up	0.001279603	0.030079677	LOC110260259
smln	rna57227	2.397555057	up	0.003183956	0.048372114	FNDC3B
smln	rna890	2.404483579	up	0.000739804	0.024625132	LOC106508849
smln	rna67767	2.416146996	up	9.39014E-05	0.011854848	TRIO
smln	rna65561	2.449398658	up	0.002690262	0.044143731	NR4A2
smln	rna15957	2.455128612	up	0.001334386	0.030627951	FBLN7
smln	rna64264	2.475213492	up	0.002146831	0.039498312	DOCK1
smln	rna64199	2.475460805	up	0.000354223	0.01889675	CTBP2
smln	rna51064	2.477505446	up	0.003364417	0.049562327	TANC2
smln	rna25184	2.500225527	up	0.001254989	0.02962028	SOX5
smln	rna62563	2.50387671	up	0.000391587	0.019648426	LRMDA
smln	rna5622	2.510128656	up	0.001148402	0.028245861	PALM2
smln	rna5146	2.520647149	up	0.003283164	0.048949295	PSAT1
smln	rna53528	2.520805866	up	0.003205488	0.048509995	GLP2R
smln	rna60721	2.522773774	up	0.003388876	0.049594835	HSPB8
smln	rna72364	2.525989392	up	0.000902067	0.026748359	CTTNBP2
smln	rna77341	2.528344066	up	0.000358916	0.018952106	LOC106508289
smln	rna65122	2.529279323	up	0.0019743	0.037812702	WWC2
smln	gene8492	2.529603158	up	0.002476159	0.042232071	LOC110260399
smln	rna69382	2.534886315	up	0.003326481	0.049194026	PSD3
smln	rna69163	2.534920496	up	0.003284794	0.048949295	DLC1
smln	rna67916	2.540568058	up	0.001018701	0.02739915	EGFLAM
smln	rna47450	2.554777417	up	0.002242118	0.039991858	IL11RA
smln	rna55895	2.558333005	up	0.001391365	0.031314356	FRMD4B
smln	rna17545	2.560182463	up	0.000841339	0.025731314	LOC102158799
smln	rna64265	2.56789392	up	0.001161948	0.028358989	FAM196A
smln	rna34864	2.575096283	up	0.000132992	0.013167335	LOC110261655
smln	rna322	2.576443879	up	0.003263388	0.048942821	TIAM2
smln	rna22913	2.582764334	up	0.000925471	0.027030692	SYDE2
smln	rna66680	2.592712977	up	0.000307537	0.018425391	PARD3B
smln	rna28984	2.597309742	up	0.000856968	0.025988414	NECTIN2
smln	rna69794	2.599542266	up	0.000659419	0.023856755	LZTS3
smln	rna43544	2.606310841	up	5.90019E-06	0.004335457	LOC102157630
smln	rna4777	2.609477446	up	0.000955561	0.027168281	LOC100514506
smln	rna23111	2.619511018	up	0.001108687	0.028055263	LOC110260611
smln	rna13544	2.637866862	up	0.002594636	0.043238416	LOC100520903

tissue	transcript_id	log2FoldChange	regulation	pvalue	padj	gene_sympo
smln	rna49580	2.638935226	up	0.002646781	0.043675622	GPC6
smln	rna26685	2.647554498	up	0.002768629	0.045112819	LOC110260826
smln	rna66679	2.656178876	up	0.001066404	0.027686213	PARD3B
smln	rna49335	2.657385564	up	0.000969207	0.027219344	DACH1
smln	gene16555	2.659350197	up	0.002871306	0.045859217	LOC110255558
smln	rna60291	2.65998225	up	0.001163797	0.028358989	UBC
smln	rna40809	2.673091683	up	0.000138077	0.013442968	ARHGAP10
smln	rna39774	2.680428145	up	0.001147413	0.028245861	RBM47
smln	rna50042	2.68150145	up	0.003196677	0.048439406	RAB40B
smln	rna22912	2.686559459	up	0.001463493	0.032078774	LOC106505390
smln	rna43167	2.690010728	up	0.000739829	0.024625132	YAP1
smln	rna34428	2.6900279	up	0.001206594	0.029246136	LOC106504294
smln	rna35832	2.700276751	up	9.05999E-05	0.011854848	COL21A1
smln	rna35979	2.706817217	up	0.000338552	0.018859419	TEAD3
smln	rna38698	2.708406855	up	0.00299422	0.046830275	FLRT2
smln	rna56801	2.71353936	up	0.000177812	0.015064659	WWTR1
smln	rna41253	2.713924744	up	0.001745676	0.035049808	ANK2
smln	rna43343	2.717276477	up	0.002200613	0.039946947	ARHGAP20
smln	rna57021	2.717537685	up	0.002169978	0.039798821	LOC100514494
smln	rna20283	2.724187702	up	0.003148756	0.048201741	LOC110260298
smln	gene23287	2.732442462	up	0.000990162	0.02738515	LOC102158170
smln	rna43543	2.737204069	up	1.60961E-06	0.002089449	LOC110255435
smln	rna3856	2.742093887	up	0.002827969	0.045571261	CALML4
smln	rna42963	2.748937943	up	0.001404755	0.031380033	FOLH1B
smln	rna24638	2.763400677	up	0.002457633	0.042192422	LOC110260695
smln	rna27967	2.777610632	up	0.003169815	0.04828286	IRX3
smln	gene21777	2.778200026	up	0.000770249	0.024722037	LOC102159217
smln	rna63924	2.784005511	up	0.001438492	0.031709255	ABLIM1
smln	gene28324	2.800697099	up	0.000949556	0.027123862	LOC110258051
smln	gene10276	2.802448121	up	0.000259056	0.017596227	LOC106507560
smln	rna41250	2.803093454	up	0.000456877	0.020218519	ARSJ
smln	rna2424	2.813337224	up	0.001327164	0.030627951	RORA
smln	rna69612	2.818452016	up	0.001615567	0.033882403	SLC24A3
smln	rna7444	2.823119397	up	9.65384E-05	0.011854848	LOC100513133
smln	rna48797	2.829146017	up	0.0016257	0.033882403	MAB21L1
smln	gene4639	2.834233202	up	1.72224E-05	0.007186061	LOC110259436
smln	rna20359	2.858987104	up	0.001715783	0.034781319	MPZL1
smln	rna39775	2.874401775	up	0.002369488	0.041593828	RBM47
smln	rna8941	2.879050836	up	0.000935473	0.02705231	LOC110259299
smln	rna18594	2.881507461	up	0.00286529	0.045856412	IQANK1
smln	rna19780	2.891635241	up	0.002594584	0.043238416	PI15
smln	rna19711	2.895982421	up	0.002620542	0.043426658	ZNF704
smln	rna34786	2.921628864	up	0.003237905	0.048810899	CAP2
smln	rna19285	2.924925041	up	0.002710691	0.044292308	CTHRC1
smln	rna24590	2.931157336	up	0.000318208	0.018794589	SLC16A7
smln	gene17637	2.931394119	up	0.000299007	0.0182895	LOC102165183
smln	rna24822	2.931702375	up	0.002891481	0.045908964	TRHDE

tissue	transcript_id	log2FoldChange	regulation	pvalue	padj	gene_sympo
smln	rna3358	2.941306512	up	0.000121801	0.012526585	ALDH1A3
smln	rna3865	2.94458465	up	4.33851E-05	0.009098641	CORO2B
smln	rna71999	2.948000859	up	6.45207E-05	0.01054228	LOC102160748
smln	rna25284	2.951592699	up	0.000547525	0.02161058	PDE3A
smln	rna58798	2.955324381	up	0.000601002	0.022942717	LOC110256448
smln	rna73275	2.955927002	up	0.000583326	0.022518808	ARHGAP6
smln	rna75111	2.960378658	up	0.000360127	0.018952106	PAK3
smln	rna50458	2.963628948	up	0.001818465	0.035706098	LOC100518330
smln	rna63201	2.964928699	up	0.000798719	0.024950368	CYP2C42
smln	rna48767	2.967911592	up	0.001611403	0.033882403	LOC110255744
smln	rna39781	2.969644527	up	0.000859816	0.025988414	RBM47
smln	rna61431	2.979525263	up	0.000835851	0.02563058	SUSD2
smln	rna55955	2.988608297	up	0.000219129	0.016101163	PDZRN3
smln	rna34825	2.991161107	up	0.003278119	0.048949295	RNF144B
smln	rna23120	2.992499937	up	0.001626982	0.033882403	LOC110260611
smln	rna5732	2.997763712	up	0.002809146	0.045393163	WDR31
smln	rna49838	3.052365177	up	0.001551333	0.033065418	LOC110255861
smln	rna33586	3.054393326	up	0.0004751	0.02054026	LOC102159014
smln	gene23150	3.058875586	up	0.001759041	0.035080564	LOC110256633
smln	rna66335	3.065969476	up	0.002854996	0.045836123	TMEFF2
smln	rna60724	3.068150922	up	0.002577338	0.043077308	LOC110256682
smln	rna36066	3.077437262	up	0.000780478	0.024753124	PI16
smln	rna76023	3.078807185	up	0.002033549	0.038505598	SLC6A8
smln	rna55702	3.08183783	up	0.000417705	0.019772346	PTPRG
smln	rna11228	3.100446971	up	0.003188447	0.048377431	GFPT2
smln	rna77598	3.10643957	up	0.001065713	0.027686213	LOC102166104
smln	gene1845	3.116798707	up	0.000390577	0.019648426	LOC100626679
smln	rna65642	3.121528602	up	0.000210316	0.015955309	TANC1
smln	rna59564	3.122816023	up	0.00149563	0.03253901	LOC110256887
smln	rna3936	3.126852279	up	1.52791E-06	0.002089449	THSD4
smln	rna73297	3.127297153	up	0.001678966	0.034473385	FRMPD4
smln	rna58215	3.132815764	up	0.000104515	0.011854894	LOC110256418
smln	rna9586	3.147481614	up	0.000246584	0.016946121	TMEM38A
smln	rna25312	3.148314181	up	0.000420826	0.019772346	PLEKHA5
smln	rna62771	3.154051148	up	0.000335786	0.018859419	LOC102164887
smln	rna19450	3.154285633	up	4.05027E-05	0.009098641	LOC110260253
smln	rna37302	3.155837307	up	0.001221229	0.029421449	SLC25A21
smln	rna67900	3.15591138	up	0.00130152	0.030371061	SLC1A3
smln	rna34827	3.157384799	up	0.000912941	0.026873125	ID4
smln	rna58968	3.158035649	up	0.002433893	0.042086329	LOC106508546
smln	rna56618	3.167360534	up	0.000402237	0.019724303	CLSTN2
smln	rna23108	3.168759772	up	0.000761935	0.024660685	CDPF1
smln	rna65585	3.175499772	up	0.001870552	0.036422769	LOC102166560
smln	rna66223	3.177803924	up	0.000527367	0.021298003	LOC110257158
smln	rna68639	3.180416206	up	1.88271E-05	0.00729584	KCNMB1
smln	rna67977	3.193853736	up	0.000239424	0.016551416	GHR
smln	rna4740	3.20625051	up	0.001730873	0.034865153	LOC110255487

tissue	transcript_id	log2FoldChange	regulation	pvalue	padj	gene_sympo
smln	rna68250	3.20752238	up	0.001013011	0.02739915	PDE4D
smln	rna68252	3.208786262	up	0.000676362	0.024091272	PDE4D
smln	rna24416	3.209687397	up	0.001759704	0.035080564	RDH16
smln	rna6030	3.227028766	up	0.000395249	0.019648426	LOC110256222
smln	rna63098	3.232148239	up	0.001024495	0.027422471	PPP1R3C
smln	rna12793	3.237382609	up	2.18569E-05	0.00729584	LRRTM2
smln	rna7110	3.237701326	up	7.14632E-05	0.011282501	LOC100738812
smln	rna2425	3.249172683	up	1.12189E-05	0.005698727	RORA
smln	rna43081	3.249193993	up	0.002331219	0.041242557	LOC110255417
smln	rna63952	3.256425903	up	0.000755916	0.024660685	LOC110256765
smln	rna40427	3.257934457	up	0.002656662	0.04377685	LOC110261977
smln	rna5109	3.259883802	up	0.002179173	0.039904823	PCSK5
smln	rna55952	3.26524087	up	0.00047727	0.02054026	GXYLT2
smln	rna16920	3.26630181	up	0.000392039	0.019648426	MEIS1
smln	rna48740	3.268338503	up	0.000877688	0.026225149	FRY
smln	rna12119	3.277207258	up	0.001758333	0.035080564	EFNA5
smln	rna70715	3.287973976	up	0.00070334	0.02456671	SLC2A10
smln	rna72551	3.292396078	up	0.000150465	0.014007824	DPY19L1
smln	rna39569	3.30202243	up	3.50495E-05	0.009098641	LOC110262064
smln	rna38363	3.304668143	up	9.53345E-05	0.011854848	LOC106507739
smln	rna26139	3.305200216	up	0.002501812	0.042360384	RAPGEF3
smln	rna69217	3.313475819	up	0.000375758	0.019363555	MTUS1
smln	rna39785	3.316094211	up	0.00174604	0.035049808	RBM47
smln	rna46539	3.324686377	up	0.000146993	0.013849393	EGFR
smln	rna48742	3.327655971	up	0.001890761	0.036572447	LOC106507937
smln	rna9634	3.331387716	up	0.001126683	0.028137847	TPM4
smln	rna49925	3.339553019	up	0.0021121	0.039260821	GRTP1
smln	rna6960	3.345501112	up	0.00167285	0.034473385	LOC110259223
smln	rna45204	3.360601285	up	9.49221E-05	0.011854848	FAM126A
smln	rna5590	3.360697636	up	9.43418E-06	0.005510976	ZNF462
smln	rna58059	3.360732982	up	0.000990892	0.02738515	LOC110256525
smln	rna23110	3.372057437	up	0.00030479	0.018354979	PPARA
smln	rna50126	3.37534994	up	0.000438078	0.019969645	CBR2
smln	rna14642	3.397524438	up	0.001330378	0.030627951	LOC110260108
smln	gene21239	3.410604556	up	0.002216336	0.039946947	LOC110256519
smln	rna70316	3.418616993	up	0.000466647	0.020495636	MYL9
smln	rna48739	3.422990228	up	0.000294787	0.0182895	LOC110255743
smln	rna36412	3.432033457	up	0.000764116	0.024660685	LOC106504420
smln	rna41466	3.43555687	up	0.002061424	0.038657499	LOC110262181
smln	rna26276	3.448357774	up	0.000502378	0.02081217	LOC110260785
smln	rna25313	3.4521848	up	0.001412414	0.031473772	PLEKHA5
smln	rna65643	3.456864707	up	9.36536E-05	0.011854848	TANC1
smln	rna2403	3.457018082	up	0.00019506	0.015345828	TLN2
smln	rna65147	3.458488098	up	0.000718671	0.024578759	STOX2
smln	rna25315	3.464419876	up	0.000992704	0.02738515	PLEKHA5
smln	rna55793	3.473829728	up	0.001380772	0.031271919	PRICKLE2
smln	rna197	3.475139116	up	0.000423143	0.0197743	SLC22A3

tissue	transcript_id	log2FoldChange	regulation	pvalue	padj	gene_sympo
smln	rna18052	3.484751551	up	0.002333419	0.041242557	OSR1
smln	gene26448	3.492293754	up	0.001962663	0.03771347	LOC102166780
smln	rna6959	3.499869331	up	0.00108718	0.027861934	LOC110259223
smln	rna49651	3.502287834	up	0.000752038	0.024660685	FARP1
smln	rna33824	3.528920831	up	0.00094385	0.027095289	LOC106508570
smln	rna64195	3.529989726	up	0.001894668	0.03658745	CTBP2
smln	gene16558	3.531850661	up	9.92595E-05	0.011854848	LOC100524035
smln	rna68259	3.539440886	up	0.000762808	0.024660685	LOC110257277
smln	rna69461	3.54016582	up	0.000231733	0.016115088	PLCB4
smln	rna68257	3.544533955	up	0.000792365	0.024897083	LOC110257279
smln	rna25294	3.549411219	up	0.000614184	0.023132674	PLEKHA5
smln	rna26817	3.549612777	up	0.002860099	0.045836123	PRDM7
smln	gene18635	3.559434184	up	0.002882067	0.045908964	LOC106505335
smln	rna27689	3.569737826	up	0.002190925	0.039935696	CMTM4
smln	rna5612	3.569752386	up	0.001152858	0.028293569	EPB41L4B
smln	rna51685	3.571043478	up	0.000506726	0.020845343	ERBB2
smln	rna68827	3.574128602	up	0.00220384	0.039946947	LOC110257243
smln	rna49654	3.579484424	up	0.000338995	0.018859419	LOC110255764
smln	rna22503	3.591177078	up	0.001003438	0.02738515	SNX7
smln	rna14242	3.59118833	up	2.17091E-05	0.00729584	LOC110260079
smln	rna56022	3.595593699	up	0.000744095	0.024660685	LOC106505659
smln	rna2877	3.595874617	up	0.000704429	0.02456671	DUOX1
smln	rna9682	3.597118631	up	0.00034704	0.018861778	CYP4F55
smln	rna50245	3.604968502	up	0.001300715	0.030371061	AATK
smln	rna12162	3.609889835	up	6.09307E-05	0.010169336	MCC
smln	rna74401	3.610988527	up	4.53972E-05	0.009304833	OPHN1
smln	rna13228	3.611222204	up	0.000488615	0.020682951	IL17B
smln	rna53893	3.611489315	up	0.000947445	0.027123862	KCNJ12
smln	gene15283	3.623596101	up	0.000342478	0.018861778	LOC102157763
smln	rna52218	3.646715215	up	0.001547247	0.033065418	RNF43
smln	rna12869	3.649125408	up	0.002435193	0.042086329	NRG2
smln	rna69459	3.657975076	up	0.000592862	0.022709522	PLCB4
smln	rna51372	3.660821841	up	0.003345738	0.04941625	ETV4
smln	rna68424	3.664282312	up	0.000419585	0.019772346	MAST4
smln	rna8284	3.667451381	up	0.001453013	0.031908937	CREB3L1
smln	rna39712	3.667967571	up	5.42019E-05	0.009940323	FAM114A1
smln	rna65626	3.670442295	up	0.000986412	0.02738515	PKP4
smln	rna23189	3.675834414	up	4.31972E-05	0.009098641	EFCAB6
smln	rna65148	3.683014563	up	0.000175718	0.015064659	STOX2
smln	rna22492	3.690703178	up	0.000693087	0.024389555	PLPPR4
smln	rna54282	3.700212804	up	0.001060058	0.027686213	CTDSPL
smln	rna26600	3.704219749	up	0.000295592	0.0182895	DCN
smln	rna20408	3.705882647	up	0.001425491	0.03154169	FAM78B
smln	rna20432	3.711205624	up	0.000774949	0.024753124	LOC110260303
smln	rna21397	3.713052146	up	0.002791991	0.045239288	LOC100513083
smln	rna35830	3.717064781	up	8.83408E-05	0.011854848	DST
smln	rna40661	3.726051323	up	1.56207E-06	0.002089449	SFRP2

tissue	transcript_id	log2FoldChange	regulation	pvalue	padj	gene_sympo
smln	rna38080	3.73315345	up	0.001400384	0.031342319	FAM174B
smln	rna3736	3.742104395	up	0.001511383	0.032699043	ATP8B1
smln	gene27146	3.74395532	up	0.000114491	0.01240824	LOC110257646
smln	rna20306	3.775999114	up	0.001243096	0.029534526	DPT
smln	rna35828	3.784414531	up	5.91822E-05	0.010020664	DST
smln	rna68942	3.790837829	up	0.000287617	0.0182895	LOC110257274
smln	rna53238	3.79344377	up	0.003160688	0.048269702	VMO1
smln	rna3645	3.803921072	up	0.000334669	0.018859419	RNF152
smln	rna62778	3.820244534	up	0.000471859	0.02054026	GDF10
smln	rna4523	3.820735041	up	0.000193244	0.015345828	LOC106509133
smln	gene26056	3.823984449	up	0.002229284	0.039946947	LOC106506037
smln	rna23816	3.824143524	up	0.001702759	0.034597104	FAIM2
smln	rna18057	3.824556919	up	3.71865E-05	0.009098641	LOC106509893
smln	rna59777	3.827432436	up	0.001135476	0.028165108	EBF2
smln	rna74395	3.834502286	up	0.00141434	0.031473772	AR
smln	rna56376	3.837982371	up	0.000755602	0.024660685	LOC110256292
smln	rna40512	3.848090175	up	0.00039628	0.019648426	MTHFD2L
smln	rna7070	3.852076611	up	0.001973389	0.037812702	LOC110259230
smln	rna13524	3.855107476	up	0.000584028	0.022518808	LOC106509619
smln	rna40657	3.855535366	up	0.003043484	0.047173964	DCHS2
smln	rna64844	3.855626981	up	0.002102984	0.039256733	GLI2
smln	rna20934	3.857949706	up	0.00195719	0.037670259	CRABP2
smln	rna58287	3.858971207	up	0.002441245	0.042128611	LOC110256529
smln	rna48771	3.863949915	up	0.00112158	0.028137847	LOC106507939
smln	rna20296	3.870424113	up	0.001784543	0.035364102	ATP1B1
smln	rna18541	3.872861799	up	0.001226043	0.02945884	OPLAH
smln	rna3317	3.879195226	up	4.18878E-05	0.009098641	SYNM
smln	rna75003	3.88234416	up	0.000825539	0.02538338	TBC1D8B
smln	rna69303	3.883781391	up	4.16599E-05	0.009098641	C17H8orf4
smln	rna68243	3.886804959	up	0.000297755	0.0182895	PDE4D
smln	rna43583	3.888241009	up	0.000751683	0.024660685	TAGLN
smln	rna67466	3.893739615	up	0.000627734	0.023169366	SH3BP4
smln	rna45357	3.894075573	up	0.000640057	0.023441322	SEMA3C
smln	rna46853	3.897153337	up	0.000501742	0.02081217	EPHX1
smln	rna65149	3.897448988	up	5.7006E-05	0.009940323	STOX2
smln	rna5460	3.897474339	up	0.000732056	0.024625132	COL15A1
smln	rna64089	3.902992423	up	0.000272538	0.0182895	FGFR2
smln	gene12566	3.906218067	up	0.000621729	0.023132674	LOC110261232
smln	rna13234	3.910077588	up	3.98868E-05	0.009098641	LOC100514340
smln	rna62650	3.926953546	up	0.003007739	0.046977831	TMEM254
smln	rna13249	3.928561204	up	0.001785913	0.035364102	ARHGEF37
smln	rna66127	3.929428791	up	0.002183055	0.03991335	PDE1A
smln	rna34860	3.932505945	up	0.000333852	0.018859419	LOC110261654
smln	rna8919	3.936005332	up	0.000216397	0.016004005	LOC110259298
smln	rna78317	3.947472701	up	0.002518094	0.042574372	LOC100737336
smln	rna68238	3.951315088	up	0.003252344	0.048902363	PDE4D
smln	rna3646	3.954884249	up	0.000353508	0.01889675	RNF152

tissue	transcript_id	log2FoldChange	regulation	pvalue	padj	gene_sympo
smln	rna69375	3.962881525	up	0.001057342	0.027686213	LOC100518097
smln	rna49740	3.975452615	up	0.000617041	0.023132674	ITGBL1
smln	gene24729	3.98063661	up	0.001211338	0.029300332	LOC110256904
smln	rna67709	3.983405147	up	0.003144607	0.048201741	FARP2
smln	gene1612	3.989968563	up	0.000206288	0.015752057	LOC100525628
smln	rna61079	3.98998665	up	0.000825617	0.02538338	LOC110256758
smln	rna12221	3.998409283	up	0.001249471	0.029549747	CDO1
smln	rna23630	4.003183325	up	0.000448908	0.020068633	RBFOX2
smln	gene17093	4.009806976	up	0.003139504	0.048201741	LOC110255483
smln	rna25308	4.011451919	up	8.26926E-05	0.011854848	PLEKHA5
smln	rna68260	4.014035313	up	0.000262226	0.017708562	LOC102161260
smln	rna15297	4.015538784	up	0.000223672	0.016115088	TNFRSF12A
smln	rna22504	4.031484618	up	5.93746E-06	0.004335457	SNX7
smln	rna13227	4.048794999	up	0.000331708	0.018859419	IL17B
smln	rna22487	4.053284525	up	0.000212336	0.016004005	LOC110260438
smln	rna8928	4.055687591	up	0.000114704	0.01240824	TEAD1
smln	rna52548	4.056995551	up	0.000943917	0.027095289	MYO1D
smln	rna24576	4.061802631	up	0.001302397	0.030371061	LOC102165680
smln	rna55071	4.065076163	up	0.001553789	0.033065418	CAMKV
smln	rna39819	4.070443397	up	4.74199E-05	0.009551842	LIMCH1
smln	rna34185	4.114934602	up	0.000972855	0.027256277	LOC100524382
smln	rna25927	4.118274325	up	0.000811152	0.025070608	WNT5B
smln	rna39544	4.122025789	up	0.00182942	0.035800853	FAM184B
smln	rna16918	4.145690802	up	0.000399142	0.019675853	MEIS1
smln	rna60750	4.149147462	up	0.001623671	0.033882403	NOS1
smln	rna24213	4.16414726	up	0.000722158	0.024578759	ITGA7
smln	rna19280	4.197702339	up	0.000404613	0.019724303	RIMS2
smln	rna37485	4.210205683	up	7.44134E-05	0.011529881	STXBP6
smln	rna39496	4.219271602	up	0.002120481	0.039260821	PROM1
smln	rna44520	4.247000835	up	0.000967828	0.027219344	MFSD4A
smln	rna54283	4.2568748	up	0.002903914	0.045908964	VILL
smln	rna4468	4.27445892	up	0.000111959	0.012339772	TMEM30B
smln	rna61877	4.276461683	up	0.000119232	0.012526585	RHOU
smln	rna62770	4.282964861	up	0.000567291	0.022018787	LOC110256824
smln	rna23500	4.290132739	up	0.001165139	0.028358989	LGALS2
smln	rna37481	4.290986533	up	0.000228298	0.016115088	STXBP6
smln	rna22412	4.291152236	up	0.001697721	0.034597104	OLFM3
smln	rna68266	4.299574126	up	0.002484173	0.04223952	LOC100523871
smln	rna61834	4.308084728	up	0.000664994	0.023978779	AGT
smln	rna48045	4.322026543	up	1.59298E-05	0.007168143	C10H10orf113
smln	rna38241	4.323641243	up	0.00214405	0.039498312	GALNT16
smln	rna62701	4.32367956	up	0.000450054	0.020068633	CDHR1
smln	rna37047	4.325286426	up	7.59908E-05	0.011529881	CSPG4
smln	rna11597	4.364529124	up	0.00034711	0.018861778	F2RL2
smln	rna77082	4.377955846	up	0.000151073	0.014007824	LOC110258316
smln	rna16693	4.390255425	up	0.000164601	0.014466519	ACTG2
smln	rna75516	4.402521254	up	0.000223329	0.016115088	GPC4

tissue	transcript_id	log2FoldChange	regulation	pvalue	padj	gene_sympo
smln	rna68492	4.403105465	up	0.000186434	0.015345828	MAP1B
smln	rna3735	4.40699648	up	0.000560165	0.021814679	ATP8B1
smln	rna34425	4.414374916	up	0.000491026	0.020709968	FOXC1
smln	rna938	4.416254712	up	0.000289787	0.0182895	RSPO3
smln	rna11973	4.434870215	up	0.003269006	0.048942821	PCSK1
smln	rna13095	4.453201933	up	0.002859745	0.045836123	LOC110259529
smln	rna23617	4.455229758	up	1.96213E-05	0.00729584	RBFOX2
smln	rna41763	4.464136336	up	0.00245427	0.042192422	LOC106507809
smln	rna77604	4.465140441	up	0.003031067	0.047153063	LOC100153854
smln	rna22486	4.480197227	up	0.000161659	0.014417255	PALMD
smln	rna20979	4.490901613	up	0.00171778	0.034781319	TMEM79
smln	rna72672	4.502067422	up	0.001382419	0.031271919	LOC106506828
smln	rna20474	4.514553585	up	0.001055224	0.027686213	C4H1orf226
smln	rna11365	4.521425155	up	0.002469758	0.042232071	DBN1
smln	rna42301	4.521974424	up	0.002903364	0.045908964	LOC106508264
smln	rna7011	4.531962381	up	0.002386445	0.041613181	SHANK2
smln	rna42323	4.542260714	up	0.001049283	0.027686213	LOC100513166
smln	rna52433	4.543501625	up	0.003271792	0.048942821	LOC110256043
smln	rna42331	4.596375562	up	0.000934033	0.02705231	LOC100511705
smln	rna7051	4.598539814	up	0.0034185	0.049821553	ANO1
smln	gene25061	4.605407755	up	0.002210133	0.039946947	LOC110257236
smln	rna37635	4.608862642	up	0.000806017	0.024977984	IL25
smln	rna42307	4.623211342	up	0.001108662	0.028055263	LOC100524384
smln	rna69088	4.635353899	up	5.367E-05	0.009940323	LOC110257476
smln	rna42303	4.635417329	up	0.002531658	0.042680177	LOC100521851
smln	rna27205	4.653406079	up	0.000101327	0.011854894	ADAMTS18
smln	rna19112	4.673003475	up	0.003029273	0.047153063	MAL2
smln	gene832	4.675948636	up	6.50266E-06	0.004416948	LOC100151775
smln	rna67612	4.679299223	up	0.000164688	0.014466519	TWIST2
smln	rna64107	4.686306627	up	1.94928E-05	0.00729584	TACC2
smln	rna42300	4.691961888	up	0.00184551	0.03599514	LOC110255376
smln	rna52549	4.695334258	up	0.002927523	0.046094674	LOC106505472
smln	rna42642	4.715948725	up	0.001094557	0.027981852	LOC106504810
smln	rna26760	4.723991101	up	0.001314563	0.030532888	LOC102165008
smln	rna14348	4.730228201	up	0.001383855	0.031271919	PRSS8
smln	rna68416	4.745053806	up	0.000915476	0.026873125	MAST4
smln	rna47443	4.758629935	up	0.00060887	0.023020786	RASEF
smln	rna39833	4.764879691	up	0.00041497	0.019772346	KCTD8
smln	rna72670	4.785234133	up	4.30649E-05	0.009098641	WIPF3
smln	rna1115	4.824522158	up	0.002780066	0.045173173	GPRC6A
smln	rna67015	4.827046508	up	1.26789E-05	0.006171989	CYP27A1
smln	rna19287	4.834117626	up	0.001128207	0.028137847	BAALC
smln	rna39637	4.850606411	up	0.000437091	0.019969645	SEL1L3
smln	rna54057	4.852652441	up	0.000865274	0.026054105	THRB
smln	rna70846	4.882309559	up	0.000657978	0.023856755	LOC106506715
smln	rna23904	4.887099282	up	0.002526224	0.042650115	KRT7
smln	rna42325	4.890441561	up	0.0009969	0.02738515	LOC100513557

tissue	transcript_id	log2FoldChange	regulation	pvalue	padj	gene_sympo
smln	rna4924	4.891991711	up	0.000104229	0.011854894	SLC1A1
smln	rna64840	4.924889483	up	0.000132883	0.013167335	TFCP2L1
smln	rna8666	4.930858304	up	0.000143371	0.01361787	SVIP
smln	rna62617	4.933991194	up	2.0448E-05	0.00729584	LOC110256684
smln	rna42009	4.943830208	up	0.00015456	0.014107235	TMEM9B
smln	rna69000	4.966430582	up	0.000430274	0.019948001	ADAMTS16
smln	rna42320	4.972129859	up	0.001020169	0.02739915	LOC100737900
smln	rna22569	4.974923396	up	0.000136799	0.013430455	F3
smln	rna22255	4.978208014	up	1.59765E-05	0.007168143	LOC106510200
smln	rna45916	4.996680695	up	0.000967109	0.027219344	LOC106505010
smln	rna74154	4.996774389	up	0.001131968	0.028137847	LOC110257837
smln	rna40594	5.015070248	up	0.001828907	0.035800853	LOC110262128
smln	rna20473	5.01889074	up	0.000196921	0.015345828	C4H1orf226
smln	rna30948	5.020802266	up	0.001700099	0.034597104	LOC110261094
smln	rna68986	5.030038903	up	0.00036723	0.019153356	SRD5A1
smln	rna31660	5.08983714	up	2.12385E-05	0.00729584	FAM46B
smln	rna63928	5.111003309	up	8.93597E-05	0.011854848	ABLIM1
smln	rna51559	5.112600533	up	0.00204129	0.038589635	LOC110255312
smln	rna22886	5.120323837	up	0.000713606	0.024578759	CLCA2
smln	rna61030	5.126313651	up	3.9303E-05	0.009098641	ACACB
smln	rna71358	5.128628883	up	0.0007906	0.024897083	OPRL1
smln	rna54037	5.152866836	up	0.000221012	0.016115088	THRB
smln	rna51598	5.208463576	up	0.000396904	0.019648426	KRT23
smln	rna33524	5.214400221	up	0.003391789	0.049594835	TACSTD2
smln	rna20146	5.231381706	up	0.00113049	0.028137847	XKR4
smln	rna46390	5.236676962	up	0.00205098	0.03864775	IRF6
smln	rna13232	5.236934785	up	5.2144E-05	0.009940323	IL17B
smln	rna63015	5.258397063	up	9.07537E-05	0.011854848	ACTA2
smln	rna22566	5.258932194	up	0.000140081	0.013525344	SLC44A3
smln	rna2203	5.259230486	up	3.26537E-05	0.009098641	RAB27B
smln	rna2357	5.267420344	up	0.002481141	0.04223952	APH1B
smln	rna68673	5.282677865	up	0.000494806	0.020765556	WWC1
smln	rna67452	5.283336412	up	0.000802204	0.024956326	UGT1A6
smln	rna65138	5.289957603	up	0.000365303	0.019138292	LOC110256910
smln	rna40592	5.328552696	up	6.69692E-05	0.010717824	SHROOM3
smln	gene9494	5.37087547	up	0.000543419	0.021521218	LOC110260740
smln	rna991	5.424451853	up	0.002950603	0.046357303	TPD52L1
smln	rna23604	5.451964213	up	1.01396E-05	0.005641008	RBFOX2
smln	rna42304	5.452488853	up	0.000721088	0.024578759	LOC100624701
smln	rna42311	5.462377659	up	0.002396475	0.041725806	LOC100521140
smln	rna39169	5.487732127	up	0.000214689	0.016004005	LOC110262031
smln	rna19690	5.495239438	up	0.000955762	0.027168281	CHMP4C
smln	gene21240	5.542097112	up	0.000177944	0.015064659	LOC110256520
smln	rna36470	5.566314163	up	0.002564301	0.042931365	ENPP5
smln	rna25721	5.58543205	up	0.002357143	0.041514379	SCNN1A
smln	rna70801	5.633932784	up	5.63876E-05	0.009940323	KCNB1
smln	rna41947	5.655127491	up	0.000620865	0.023132674	LOC110262199

tissue	transcript_id	log2FoldChange	regulation	pvalue	padj	gene_sympo
smln	rna19422	5.709349819	up	2.36933E-05	0.007606743	NIPAL2
smln	rna36223	5.718401134	up	0.000291643	0.0182895	C7H6orf132
smln	rna23634	5.722654728	up	0.000847441	0.025850275	LOC106510287
smln	rna24644	5.728290938	up	0.000620538	0.023132674	C5H12orf56
smln	rna47947	5.788423344	up	0.000444476	0.020049481	LOC110255698
smln	rna1840	5.809849539	up	0.000323954	0.018794589	ME1
smln	rna24575	5.837001565	up	0.000193612	0.015345828	LOC106510313
smln	rna8641	5.887119012	up	0.000390666	0.019648426	LGR4
smln	rna37303	5.895622794	up	0.000193874	0.015345828	PAX9
smln	rna43556	5.927165109	up	0.001562188	0.033183707	NXPE2
smln	rna1782	5.932450803	up	0.001589923	0.033711559	FRK
smln	rna1110	5.933089935	up	0.001221382	0.029421449	RFX6
smln	rna42334	5.944983557	up	0.00040519	0.019724303	LOC100514670
smln	rna4454	5.948912064	up	0.000537443	0.021516991	SIX6
smln	rna8926	5.952798023	up	2.40905E-05	0.007606743	TEAD1
smln	rna19421	5.981727568	up	0.0016516	0.034151584	NIPAL2
smln	rna10902	5.984339717	up	0.000776645	0.024753124	LOC110259662
smln	gene7664	5.997892052	up	0.00094347	0.027095289	LOC110260304
smln	gene3870	6.013689006	up	0.000322807	0.018794589	LOC100517213
smln	rna23362	6.01668337	up	0.000216437	0.016004005	FAM83F
smln	rna44593	6.054549239	up	0.000231017	0.016115088	PIGR
smln	rna11533	6.073982445	up	0.000651437	0.023783552	ARHGEF28
smln	rna19641	6.137731828	up	8.04421E-05	0.011747564	LOC100154873
smln	rna42310	6.143958865	up	0.001333183	0.030627951	LOC110255381
smln	rna39601	6.204081514	up	0.002157721	0.039636258	PPARGC1A
smln	rna49785	6.247203337	up	9.05006E-05	0.011854848	LOC102167506
smln	rna43650	6.248894122	up	0.000159299	0.014316064	MPZL2
smln	rna4458	6.251769845	up	7.84894E-05	0.011747564	SIX4
smln	rna4457	6.265572513	up	0.001507624	0.032678239	LOC106509125
smln	rna64123	6.304754422	up	0.002325208	0.041222164	LOC100519221
smln	rna26337	6.313552854	up	0.003111957	0.047966702	SLC5A8
smln	rna30520	6.32397509	up	0.002374654	0.041593828	LOC100620540
smln	rna1113	6.331986752	up	0.000275466	0.0182895	LOC110260274
smln	rna36936	6.349727101	up	0.000463694	0.020442801	LOC110261554
smln	rna53324	6.352291463	up	1.30532E-06	0.002089449	SLC2A4
smln	rna39820	6.407257614	up	3.89686E-06	0.003251926	LOC110262081
smln	rna77443	6.44890477	up	7.97673E-05	0.011747564	LOC110255234
smln	rna69002	6.44939265	up	0.000197028	0.015345828	IRX1
smln	rna8500	6.460136822	up	0.000200294	0.015394949	EHF
smln	rna33571	6.49026	up	0.001446907	0.031834671	C6H1orf168
smln	rna57260	6.499345536	up	5.68945E-05	0.009940323	NAALADL2
smln	rna25447	6.61138749	up	0.00184157	0.035978358	SL44-1
smln	rna42327	6.640983006	up	0.000110493	0.012294165	LOC100513749
smln	rna36935	6.687341702	up	0.000502379	0.02081217	WDR93
smln	rna57086	6.693677141	up	1.29433E-06	0.002089449	BCHE
smln	rna13618	6.792219684	up	0.001614323	0.033882403	NPTX2
smln	rna28911	6.887762967	up	3.85563E-05	0.009098641	KCNN4

tissue	transcript_id	log2FoldChange	regulation	pvalue	padj	gene_sympo
smln	rna33869	6.908430647	up	3.04697E-05	0.009098641	LOC100523909
smln	rna17322	6.933747534	up	0.000156777	0.014198617	EPCAM
smln	rna23941	6.951219641	up	5.20278E-05	0.009940323	KRT18
smln	rna4456	6.963192298	up	2.95619E-06	0.002878102	SIX1
smln	rna13803	7.0365539	up	0.00052535	0.021298003	LOC110259869
smln	rna23939	7.048337689	up	9.78491E-05	0.011854848	KRT8
smln	rna42732	7.130873375	up	4.36124E-05	0.009098641	THRSP
smln	rna51619	7.137161622	up	9.94415E-05	0.011854848	TNS4
smln	rna8501	7.179067448	up	0.000101517	0.011854894	EHF
smln	rna77444	7.197539605	up	0.00221357	0.039946947	LOC100736962
smln	rna59368	7.232972928	up	7.55769E-05	0.011529881	TMPRSS2
smln	rna65575	7.308244544	up	0.000725813	0.024578759	GALNT5
smln	rna65124	7.329531538	up	0.000141859	0.013584773	CLDN22
smln	rna10472	7.473146941	up	0.000117816	0.012513183	LRRC8E
smln	rna76022	7.552097334	up	0.000153792	0.014107235	PNCK
smln	rna4455	7.70746889	up	1.65659E-05	0.007168143	LOC110255392
smln	rna31225	7.776787258	up	3.0022E-05	0.009098641	KLHDC7A
smln	rna76298	7.79276824	up	0.001541223	0.033065418	LOC110257905
smln	rna75183	7.79294873	up	0.002564931	0.042931365	SLC6A14
smln	rna52207	8.135794778	up	0.000382888	0.01961963	LPO
smln	rna50282	8.173161706	up	1.10797E-05	0.005698727	CARD14
smln	rna15359	8.197872867	up	0.000995127	0.02738515	ZG16B
smln	rna71906	8.350185452	up	2.71143E-06	0.002878102	ATP6V0A4
smln	rna59390	8.570763129	up	0.000393229	0.019648426	TFF3
smln	rna34423	9.020441776	up	0.000453384	0.02014025	LOC106504293
smln	rna52451	9.274654587	up	2.02341E-06	0.002363952	LOC110256048
smln	rna23819	9.475111507	up	0.000344536	0.018861778	AQP5
smln	rna70070	10.33329632	up	0.000275577	0.0182895	BPIFB2
smln	rna36543	10.42208094	up	0.000682538	0.024237351	CRISP3
smln	rna7718	11.10014357	up	0.000124608	0.012659072	PHEROC
smln	rna5713	12.5000215	up	4.91774E-07	0.001436349	SAL1
smln	rna40382	13.45553365	up	9.47352E-05	0.011854848	CSN1S2
smln	rna69012	21.9590953	up	1.89998E-10	7.39917E-07	LOC110257297
smln	rna68017	23.1489518	up	6.90122E-12	4.03135E-08	LOC106508153
smln	rna7719	27.054966	up	4.18979E-15	4.89493E-11	SCGB1D1
spleen	rna44904	-4.975734559	down	7.36296E-14	9.31561E-10	PDK4
spleen	rna57758	-4.514441293	down	5.2822E-07	0.000668304	CLDN1
spleen	gene12586	-4.186187266	down	0.000210055	0.011314166	LOC100627844
spleen	rna56663	-4.157466473	down	0.000122044	0.009304813	LOC100739719
spleen	rna641	-4.126003784	down	2.74818E-07	0.000434624	PERP
spleen	rna36620	-3.920940173	down	8.60266E-08	0.000220281	CHRNA3
spleen	rna31875	-3.762494249	down	1.44598E-06	0.001016362	FABP3
spleen	rna65150	-3.661748254	down	9.58664E-07	0.000808601	ENPP6
spleen	rna8650	-3.614961911	down	1.05755E-06	0.000836257	FIBIN
spleen	rna1913	-3.512861415	down	1.52246E-05	0.004098344	HTR1B
spleen	rna75000	-3.440406942	down	8.70539E-08	0.000220281	CXHXorf57
spleen	rna56796	-3.411636175	down	3.47377E-06	0.001910877	CP

tissue	transcript_id	log2FoldChange	regulation	pvalue	padj	gene_sympo
spleen	rna27356	-3.397828584	down	1.3722E-05	0.00391546	TAT
spleen	rna694	-3.332054343	down	0.000141826	0.009654685	MAP7
spleen	rna68368	-3.31465088	down	8.35086E-05	0.008319302	NLN
spleen	rna21362	-3.236046105	down	0.000307	0.013434353	S100A12
spleen	rna6465	-3.171477352	down	0.004430335	0.042113147	LOC100510887
spleen	rna23131	-3.126597341	down	0.000140816	0.009654685	FBLN1
spleen	rna20014	-3.084992604	down	1.39263E-05	0.00391546	GGH
spleen	rna47074	-3.059755066	down	1.30607E-06	0.000972021	ATP6V1G3
spleen	rna58611	-3.033738568	down	0.000554002	0.016649024	ZPLD1
spleen	rna64684	-2.930190596	down	0.001232066	0.022657119	LOC110257013
spleen	rna10728	-2.905955729	down	0.001171906	0.021998441	LRG1
spleen	rna12157	-2.830231809	down	7.85337E-05	0.00829475	STARD4
spleen	rna37659	-2.785808652	down	7.96896E-05	0.00829475	SLC7A8
spleen	rna5515	-2.746373842	down	0.000343932	0.013987019	LOC102167599
spleen	rna52671	-2.712176208	down	9.01824E-05	0.00849098	NLK
spleen	rna44988	-2.708470516	down	0.000202954	0.011314166	NDUFA4
spleen	rna8918	-2.701630722	down	6.871E-05	0.00829475	RASSF10
spleen	rna27490	-2.699334629	down	8.12954E-05	0.00829475	MT-2B
spleen	rna65452	-2.656107664	down	0.004479687	0.042264724	OCA2
spleen	rna23939	-2.655530151	down	4.1715E-05	0.00659083	KRT8
spleen	rna23904	-2.641212927	down	6.94132E-05	0.00829475	KRT7
spleen	rna23130	-2.64046734	down	0.00012902	0.009364248	FBLN1
spleen	rna63657	-2.559858534	down	5.60523E-05	0.007629569	USMG5
spleen	rna63714	-2.557005844	down	6.21354E-05	0.0081045	GSTO1
spleen	rna13758	-2.537789158	down	1.19405E-07	0.000251785	CYP3A46
spleen	rna39846	-2.527340735	down	0.002834522	0.033322151	GABRG1
spleen	rna43659	-2.525825446	down	3.80743E-05	0.006509684	ATP5L
spleen	rna58467	-2.517636254	down	0.004970098	0.044660288	C13H3orf52
spleen	rna67983	-2.491420628	down	0.000809983	0.01967595	CCDC152
spleen	rna67240	-2.481766798	down	0.001604393	0.025488322	DNER
spleen	rna64043	-2.472742027	down	0.000348844	0.013987019	PRDX3
spleen	rna24916	-2.463985998	down	0.002172407	0.029208568	FGD4
spleen	rna33144	-2.4347428	down	7.93654E-05	0.00829475	LOC110261401
spleen	rna49602	-2.430233593	down	0.002231752	0.029564197	CLDN10
spleen	rna1783	-2.415045929	down	0.000127149	0.009304813	NT5DC1
spleen	rna53106	-2.396988295	down	3.15394E-05	0.006190614	TXNDC17
spleen	rna28136	-2.389281055	down	0.000115499	0.009304813	NUDT19
spleen	rna1556	-2.383337466	down	0.004579002	0.042660925	LOC100519934
spleen	rna44370	-2.381927839	down	0.000915672	0.020243112	FMO2
spleen	rna7355	-2.378380849	down	0.001791058	0.026624653	CST6
spleen	rna27257	-2.359215781	down	0.004535081	0.042502111	BCAR1
spleen	rna40661	-2.357204017	down	0.000120223	0.009304813	SFRP2
spleen	rna73428	-2.345692168	down	0.000205745	0.011314166	CXH4orf3
spleen	rna21994	-2.342129024	down	0.002576667	0.031706645	CASQ2
spleen	rna59996	-2.339950191	down	0.00021696	0.011389961	HPGD
spleen	rna72761	-2.313328904	down	0.003201605	0.035205886	LOC106506838
spleen	rna56798	-2.292270781	down	8.86579E-05	0.008467388	TM4SF18

tissue	transcript_id	log2FoldChange	regulation	pvalue	padj	gene_sympo
spleen	rna35996	-2.285515165	down	0.000168248	0.010537982	LOC110261510
spleen	rna66127	-2.272478727	down	0.003988793	0.039643528	PDE1A
spleen	rna41959	-2.27045073	down	1.2066E-08	7.63294E-05	BMP3
spleen	rna39440	-2.261345956	down	0.00036068	0.014084349	LOC100525472
spleen	rna75221	-2.248889423	down	8.46269E-05	0.00834202	SLC25A5
spleen	rna62910	-2.224363032	down	4.74098E-05	0.007140823	CISD1
spleen	rna57813	-2.218762554	down	0.003873208	0.038984746	ATP13A3
spleen	rna60091	-2.192864335	down	0.000251325	0.012434148	CBR4
spleen	rna38896	-2.184183106	down	0.000662338	0.01779171	LOC100156325
spleen	rna59117	-2.179530096	down	0.001119535	0.021713066	SCAF4
spleen	rna59116	-2.174496846	down	0.000213543	0.011314166	SOD1
spleen	rna45814	-2.16280396	down	0.002781403	0.033117891	PRDX6
spleen	rna55874	-2.146020571	down	0.001420195	0.024053965	SUCLG2
spleen	rna34887	-2.133810729	down	8.76367E-05	0.008467388	TDP2
spleen	rna75268	-2.129811459	down	0.000588347	0.01708647	LAMP2
spleen	rna54617	-2.127405197	down	0.000126036	0.009304813	CDCP1
spleen	rna40981	-2.126553661	down	8.78113E-05	0.008467388	NDUFC1
spleen	rna8079	-2.124258659	down	0.000313736	0.013434353	MTCH2
spleen	rna69209	-2.120611161	down	0.004566474	0.042606954	MTMR7
spleen	rna59642	-2.116945805	down	0.000526113	0.016156269	LPL
spleen	rna24392	-2.113267906	down	0.000104056	0.00916764	ATP5B
spleen	rna16969	-2.109282259	down	0.000607297	0.017318581	MDH1
spleen	rna56776	-2.104293822	down	0.0002143	0.011314166	AGTR1
spleen	rna60280	-2.069846174	down	1.23379E-05	0.003761624	BRI3BP
spleen	rna17160	-2.067555757	down	0.000202994	0.011314166	EFEMP1
spleen	rna42936	-2.066889373	down	8.32498E-06	0.003191746	CTSC
spleen	rna67916	-2.060248291	down	5.8489E-06	0.002551734	EGFLAM
spleen	rna34970	-2.057684838	down	0.000198259	0.011298995	LOC110261477
spleen	rna40430	-2.057206927	down	0.003388997	0.036198036	GC
spleen	rna65993	-2.052377034	down	9.40167E-05	0.008619557	ATP5G3
spleen	rna66407	-2.051437145	down	0.000109189	0.009304813	HSPE1
spleen	rna40940	-2.039446723	down	0.001405802	0.023970621	SCOC
spleen	rna1942	-2.035555239	down	0.000148021	0.009908774	COX7A2
spleen	rna46853	-2.024613804	down	0.002967282	0.03400073	EPHX1
spleen	rna31550	-2.022189915	down	0.000881409	0.020079589	CLIC4
spleen	rna45830	-2.009886425	down	0.004348774	0.041682339	SERPINC1
spleen	rna28050	2.001506652	up	0.002882076	0.033619673	LOC110260968
spleen	rna30121	2.005542052	up	0.00141942	0.024053965	LOC110261066
spleen	rna48342	2.015725263	up	0.000959112	0.02063723	LOC110255727
spleen	rna61228	2.021842468	up	0.000117854	0.009304813	MTMR3
spleen	rna56027	2.022985574	up	0.005458252	0.046914266	LRRN1
spleen	rna34292	2.023008128	up	0.000533737	0.016289805	SLFNL1
spleen	rna63087	2.023652648	up	0.000167567	0.010537982	PCGF5
spleen	rna69026	2.043225799	up	0.004666686	0.043097017	LPCAT1
spleen	gene16170	2.059866901	up	3.50402E-05	0.006190614	LOC100523853
spleen	rna19009	2.067903883	up	5.60574E-05	0.007629569	LOC106509974
spleen	rna70319	2.075500753	up	1.13083E-05	0.003576807	TGIF2

tissue	transcript_id	log2FoldChange	regulation	pvalue	padj	gene_sympo
spleen	rna31422	2.091895176	up	0.000883554	0.020079589	LOC110261112
spleen	rna27060	2.092556646	up	0.00269706	0.032529273	LOC106510461
spleen	rna46080	2.093635046	up	0.000374452	0.014269793	NMNAT2
spleen	rna7290	2.099021004	up	0.005400269	0.046701441	BBS1
spleen	rna67709	2.115287585	up	0.002233906	0.029564197	FARP2
spleen	rna29951	2.124570897	up	0.005561004	0.047474916	LOC100624191
spleen	rna8203	2.125612255	up	0.002306957	0.030090333	NR1H3
spleen	rna68492	2.151093777	up	0.001150141	0.021869506	MAP1B
spleen	rna69024	2.158469826	up	0.003108791	0.034715286	LPCAT1
spleen	rna13238	2.241115212	up	0.000384599	0.01448197	MIR145
spleen	rna13212	2.261056742	up	0.004773513	0.04370079	GRPEL2
spleen	rna13866	2.29084148	up	0.002148226	0.029057761	ZCWPW1
spleen	rna18550	2.310983785	up	0.000665267	0.017794845	PLEC
spleen	rna49967	2.314041318	up	0.000397522	0.014638889	LOC106504762
spleen	rna43658	2.34371773	up	0.000999137	0.020760473	LOC102167309
spleen	rna47839	2.388837297	up	0.001092512	0.021392395	FRMD4A
spleen	rna23890	2.421824106	up	0.000855249	0.019821666	ACVRL1
spleen	rna15042	2.424763299	up	0.00298342	0.034005615	LOC110260090
spleen	rna53923	2.432216638	up	0.000193485	0.011224954	LOC100739656
spleen	rna6661	2.433760424	up	0.000294628	0.013218577	FCN1
spleen	rna64178	2.4377101	up	2.62311E-05	0.006034112	FAM53B
spleen	rna38234	2.44428005	up	0.004705271	0.043421655	LOC106508572
spleen	rna26966	2.510854605	up	0.000480388	0.015322324	LOC110260888
spleen	rna68000	2.539122512	up	0.002459804	0.031156245	LOC102162264
spleen	rna19882	2.549488663	up	0.000289689	0.013191687	NCOA2
spleen	rna7204	2.661469817	up	0.000837977	0.019756702	CLCF1
spleen	rna53538	2.759660653	up	6.48647E-05	0.008289573	GAS7
spleen	rna63254	2.767905624	up	0.005405665	0.046711657	LOC102166928
spleen	rna29239	2.952306588	up	3.99459E-05	0.00659083	CCDC114
spleen	rna78516	3.11065037	up	0.002528475	0.031455527	LOC102159510
spleen	rna44297	3.211623605	up	0.000284069	0.013116941	LOC110262224
spleen	rna22898	6.045144694	up	6.71141E-05	0.00829475	LOC106510256
