# Supplementary material for: Transcriptome analysis reveals gene expression changes of pigs infected with non-lethal African swine fever virus
Source: Genet Mol Biol. 2023 Oct 13;46(3):e20230037. doi: 10.1590/1678-4685-GMB-2023-0037 (PMC10578457; doi:10.1590/1678-4685-GMB-2023-0037)
Supplement: Table S3 - [file 1415-4757-GMB-46-3-e20230037-s5.pdf]

Genetics and Molecular Biology

https://doi.org/10.1590/1678-4685-GMB-2023-0037

# Supplementary Material to "Transcriptome analysis reveals gene expression changes of pigs infected with non-lethal African swine fever virus"

**Table S3** - The enriched pathways of DEGs in kidney using KOBAS 3.0

| #Term | Database | ID | Input number | Corrected P-Value |
|---|---|---|---|---|
| protein binding | Gene Ontology | GO:0005515 | 61 | 7.45E-06 |
| extracellular space | Gene Ontology | GO:0005615 | 20 | 8.02E-06 |
| collagen-containing extracellular matrix | Gene Ontology | GO:0062023 | 10 | 5.46E-05 |
| extracellular exosome | Gene Ontology | GO:0070062 | 19 | 0.001208 |
| cytosol | Gene Ontology | GO:0005829 | 31 | 0.002663 |
| cytoplasm | Gene Ontology | GO:0005737 | 29 | 0.00278 |
| response to vitamin D | Gene Ontology | GO:0033280 | 3 | 0.004166 |
| extracellular region | Gene Ontology | GO:0005576 | 16 | 0.00788 |
| blood microparticle | Gene Ontology | GO:0072562 | 5 | 0.009194 |
| signaling receptor binding | Gene Ontology | GO:0005102 | 7 | 0.009639 |
| plasma membrane | Gene Ontology | GO:0005886 | 27 | 0.010604 |
| triglyceride homeostasis | Gene Ontology | GO:0070328 | 3 | 0.010604 |
| defense response to Gram-negative bacterium | Gene Ontology | GO:0050829 | 4 | 0.010604 |
| endopeptidase activity | Gene Ontology | GO:0004175 | 4 | 0.010604 |
| regulation of cytosolic calcium ion concentration | Gene Ontology | GO:0051480 | 3 | 0.024096 |
| negative regulation of cell migration | Gene Ontology | GO:0030336 | 4 | 0.02837 |
| response to bacterium | Gene Ontology | GO:0009617 | 4 | 0.02837 |
| tissue remodeling | Gene Ontology | GO:0048771 | 2 | 0.02837 |
| chylomicron remodeling | Gene Ontology | GO:0034371 | 2 | 0.031604 |
| very-low-density lipoprotein particle remodeling | Gene Ontology | GO:0034372 | 2 | 0.031604 |
| membrane | Gene Ontology | GO:0016020 | 15 | 0.036173 |
| manganese ion transport | Gene Ontology | GO:0006828 | 2 | 0.036812 |
| nucleus | Gene Ontology | GO:0005634 | 27 | 0.036812 |
| extracellular matrix structural constituent | Gene Ontology | GO:0005201 | 4 | 0.036812 |
| collagen-activated tyrosine kinase receptor signaling pathway | Gene Ontology | GO:0038063 | 2 | 0.038592 |
| chylomicron | Gene Ontology | GO:0042627 | 2 | 0.040851 |
| external side of plasma membrane | Gene Ontology | GO:0009897 | 6 | 0.040851 |
| metallopeptidase activity | Gene Ontology | GO:0008237 | 3 | 0.040851 |
| negative regulation of JUN kinase activity | Gene Ontology | GO:0043508 | 2 | 0.040851 |

| #Term | Database | ID | Input number | Corrected P-Value |
|---|---|---|---|---|
| actin binding | Gene Ontology | GO:0003779 | 5 | 0.040851 |
| extracellular matrix disassembly | Gene Ontology | GO:0022617 | 3 | 0.040851 |
| regulation of long-term synaptic potentiation | Gene Ontology | GO:1900271 | 2 | 0.041361 |
| stress fiber | Gene Ontology | GO:0001725 | 3 | 0.042027 |
| apolipoprotein binding | Gene Ontology | GO:0034185 | 2 | 0.04342 |
| heparin binding | Gene Ontology | GO:0008201 | 4 | 0.044128 |
| positive regulation of calcium ion import | Gene Ontology | GO:0090280 | 2 | 0.045477 |
| metal ion binding | Gene Ontology | GO:0046872 | 15 | 0.049597 |
| Apelin signaling pathway | KEGG PATHWAY | hsa04371 | 4 | 0.036812 |
| Nitrogen metabolism | KEGG PATHWAY | hsa00910 | 2 | 0.045477 |
| PPAR signaling pathway | KEGG PATHWAY | hsa03320 | 3 | 0.048373 |
| Signal Transduction | Reactome | R-HSA-162582 | 22 | 0.001208 |
| Developmental Biology | Reactome | R-HSA-1266738 | 11 | 0.021175 |
| Metabolism | Reactome | R-HSA-1430728 | 15 | 0.036173 |
| Signaling by PDGF | Reactome | R-HSA-186797 | 3 | 0.036812 |
| Transport of small molecules | Reactome | R-HSA-382551 | 8 | 0.040851 |
| Regulation of innate immune responses to cytosolic DNA | Reactome | R-HSA-3134975 | 2 | 0.041361 |
| MAPK family signaling cascades | Reactome | R-HSA-5683057 | 5 | 0.043233 |
