# Supplementary material for: Transcriptome analysis reveals gene expression changes of pigs infected with non-lethal African swine fever virus
Source: Genet Mol Biol. 2023 Oct 13;46(3):e20230037. doi: 10.1590/1678-4685-GMB-2023-0037 (PMC10578457; doi:10.1590/1678-4685-GMB-2023-0037)
Supplement: Table S4 - [file 1415-4757-GMB-46-3-e20230037-s6.pdf]

Genetics and Molecular Biology

https://doi.org/10.1590/1678-4685-GMB-2023-0037

Supplementary Material to "Transcriptome analysis reveals gene expression changes of pigs infected with non-lethal African swine fever virus"

Table S4 - The enriched pathways of DEGs in liver using KOBAS 3.0.

#Term	Database	ID	Input number	Corrected P-Value
protein binding	Gene Ontology	GO:0005515	169	4.73E-23
cytosol	Gene Ontology	GO:0005829	88	7.89E-13
extracellular space	Gene Ontology	GO:0005615	41	8.1E-10
plasma membrane	Gene Ontology	GO:0005886	74	5.36E-09
extracellular exosome	Gene Ontology	GO:0070062	46	5.36E-09
cytoplasm	Gene Ontology	GO:0005737	70	2.02E-07
extracellular region	Gene Ontology	GO:0005576	37	4.6E-06
integral component of membrane	Gene Ontology	GO:0016021	55	1.61E-05
ATP binding	Gene Ontology	GO:0005524	30	6.08E-05
negative regulation of transcription by RNA polymerase II	Gene Ontology	GO:0000122	21	0.000152
lipid homeostasis	Gene Ontology	GO:0055088	6	0.000226
positive regulation of triglyceride catabolic process	Gene Ontology	GO:0010898	4	0.000269
positive regulation of lipoprotein lipase activity	Gene Ontology	GO:0051006	4	0.000357
nucleus	Gene Ontology	GO:0005634	65	0.000432
nucleoplasm	Gene Ontology	GO:0005654	50	0.000562
positive regulation of fatty acid biosynthetic process	Gene Ontology	GO:0045723	4	0.000562
intrinsic apoptotic signaling pathway in response to endoplasmic reticulum stress	Gene Ontology	GO:0070059	5	0.001033
cellular protein metabolic process	Gene Ontology	GO:0044267	9	0.002038
identical protein binding	Gene Ontology	GO:0042802	26	0.002123
oxidation-reduction process	Gene Ontology	GO:0055114	14	0.003268
membrane	Gene Ontology	GO:0016020	32	0.003351
carbohydrate binding	Gene Ontology	GO:0030246	8	0.00352
metal ion binding	Gene Ontology	GO:0046872	34	0.00352
cell-cell signaling	Gene Ontology	GO:0007267	9	0.00352
pigmentation	Gene Ontology	GO:0043473	4	0.004474
regulation of lipid metabolic process	Gene Ontology	GO:0019216	6	0.005219
O-glycan processing	Gene Ontology	GO:0016266	5	0.006309
response to salt stress	Gene Ontology	GO:0009651	3	0.006479

#Term	Database	ID	Input number	Corrected P-Value
serine-type endopeptidase inhibitor activity	Gene Ontology	GO:0004867	6	0.006524
cytoplasmic vesicle membrane	Gene Ontology	GO:0030659	7	0.006691
FAD binding	Gene Ontology	GO:0071949	4	0.006849
axonemal dynein complex	Gene Ontology	GO:0005858	3	0.007339
negative regulation of T cell activation	Gene Ontology	GO:0050868	3	0.008443
endoplasmic reticulum membrane	Gene Ontology	GO:0005789	18	0.009232
response to arsenic-containing substance	Gene Ontology	GO:0046685	3	0.009671
innate immune response	Gene Ontology	GO:0045087	13	0.011133
mitochondrial outer membrane	Gene Ontology	GO:0005741	7	0.011572
detection of chemical stimulus involved in sensory perception of bitter taste	Gene Ontology	GO:0001580	4	0.013637
calcium-dependent protein binding	Gene Ontology	GO:0048306	5	0.014436
nuclear receptor activity	Gene Ontology	GO:0004879	4	0.014436
endoplasmic reticulum calcium ion homeostasis	Gene Ontology	GO:0032469	3	0.016336
cellular response to amino acid starvation	Gene Ontology	GO:0034198	4	0.016336
intracellular signal transduction	Gene Ontology	GO:0035556	10	0.016498
integral component of plasma membrane	Gene Ontology	GO:0005887	22	0.016972
endocytosis	Gene Ontology	GO:0006897	7	0.016981
ATP-dependent microtubule motor activity, minus-end-directed	Gene Ontology	GO:0008569	3	0.017085
cholesterol homeostasis	Gene Ontology	GO:0042632	5	0.017085
neuronal cell body	Gene Ontology	GO:0043025	10	0.017329
triglyceride biosynthetic process	Gene Ontology	GO:0019432	3	0.018022
response to vitamin A	Gene Ontology	GO:0033189	3	0.018022
long-chain fatty-acyl-CoA biosynthetic process	Gene Ontology	GO:0035338	3	0.018022
animal organ morphogenesis	Gene Ontology	GO:0009887	6	0.018022
response to lipopolysaccharide	Gene Ontology	GO:0032496	6	0.019878
DNA-binding transcription repressor activity, RNA polymerase II-specific	Gene Ontology	GO:0001227	8	0.019878
neuron projection terminus	Gene Ontology	GO:0044306	3	0.019878
host cell	Gene Ontology	GO:0043657	4	0.020341
xenobiotic metabolic process	Gene Ontology	GO:0006805	5	0.020874
low-density lipoprotein particle receptor binding	Gene Ontology	GO:0050750	3	0.021659
calmodulin binding	Gene Ontology	GO:0005516	7	0.023046
cellular defense response	Gene Ontology	GO:0006968	4	0.023046
carbohydrate phosphorylation	Gene Ontology	GO:0046835	3	0.023046
long-chain fatty acid metabolic process	Gene Ontology	GO:0001676	3	0.023046
response to light stimulus	Gene Ontology	GO:0009416	3	0.023046
pyridoxal phosphate binding	Gene Ontology	GO:0030170	4	0.02423
tissue regeneration	Gene Ontology	GO:0042246	3	0.025419
negative regulation of endopeptidase activity	Gene Ontology	GO:0010951	6	0.026072
secretory granule membrane	Gene Ontology	GO:0030667	5	0.026902

#Term	Database	ID	Input number	Corrected P-Value
Golgi apparatus	Gene Ontology	GO:0005794	17	0.027992
Golgi lumen	Gene Ontology	GO:0005796	5	0.027992
dynein complex	Gene Ontology	GO:0030286	3	0.028246
regulation of axon diameter	Gene Ontology	GO:0031133	2	0.028246
regulation of intestinal cholesterol absorption	Gene Ontology	GO:0030300	2	0.028246
apoptotic process involved in blood vessel morphogenesis	Gene Ontology	GO:1902262	2	0.028246
fructose-2,6-bisphosphate 2-phosphatase activity	Gene Ontology	GO:0004331	2	0.028246
fructose 2,6-bisphosphate metabolic process	Gene Ontology	GO:0006003	2	0.028246
positive regulation of glomerular mesangial cell proliferation	Gene Ontology	GO:0072126	2	0.028246
response to drug	Gene Ontology	GO:0042493	8	0.028517
signal transduction	Gene Ontology	GO:0007165	17	0.028559
cellular response to glucose stimulus	Gene Ontology	GO:0071333	4	0.029171
learning	Gene Ontology	GO:0007612	4	0.029171
axoneme	Gene Ontology	GO:0005930	5	0.029351
enzyme binding	Gene Ontology	GO:0019899	9	0.029415
cell surface receptor signaling pathway	Gene Ontology	GO:0007166	8	0.030504
dynein light intermediate chain binding	Gene Ontology	GO:0051959	3	0.030812
aging	Gene Ontology	GO:0007568	6	0.030812
cell differentiation	Gene Ontology	GO:0030154	12	0.030812
defense response to fungus	Gene Ontology	GO:0050832	3	0.030812
triglyceride homeostasis	Gene Ontology	GO:0070328	3	0.030812
catecholamine biosynthetic process	Gene Ontology	GO:0042423	2	0.030812
regulation of nucleocytoplasmic transport	Gene Ontology	GO:0046822	2	0.030812
norepinephrine biosynthetic process	Gene Ontology	GO:0042421	2	0.030812
phosphatidylcholine-sterol O-acyltransferase activator activity	Gene Ontology	GO:0060228	2	0.030812
molybdopterin cofactor binding	Gene Ontology	GO:0043546	2	0.030812
blood coagulation, fibrin clot formation	Gene Ontology	GO:0072378	2	0.030812
fatty acid metabolic process	Gene Ontology	GO:0006631	4	0.030859
regulation of circadian rhythm	Gene Ontology	GO:0042752	4	0.031929
stimulatory C-type lectin receptor signaling pathway	Gene Ontology	GO:0002223	5	0.031975
leukocyte cell-cell adhesion	Gene Ontology	GO:0007159	3	0.032169
platelet alpha granule lumen	Gene Ontology	GO:0031093	4	0.033004
secretory granule lumen	Gene Ontology	GO:0034774	5	0.033575
dynein intermediate chain binding	Gene Ontology	GO:0045505	3	0.034083
L-serine transport	Gene Ontology	GO:0015825	2	0.034892
modulation by virus of host process	Gene Ontology	GO:0019048	2	0.034892
response to herbicide	Gene Ontology	GO:0009635	2	0.034892
L-serine transmembrane transporter activity	Gene Ontology	GO:0015194	2	0.034892
chromaffin granule membrane	Gene Ontology	GO:0042584	2	0.034892

#Term	Database	ID	Input number	Corrected P-Value
negative regulation of dendritic spine morphogenesis	Gene Ontology	GO:0061002	2	0.034892
response to zinc ion	Gene Ontology	GO:0010043	3	0.034912
stress fiber	Gene Ontology	GO:0001725	4	0.034912
mitochondrion	Gene Ontology	GO:0005739	19	0.035095
oxygen binding	Gene Ontology	GO:0019825	3	0.037155
axon	Gene Ontology	GO:0030424	8	0.037346
regulation of heart contraction	Gene Ontology	GO:0008016	3	0.038385
actin cytoskeleton	Gene Ontology	GO:0015629	7	0.038385
glutamine transport	Gene Ontology	GO:0006868	2	0.038385
peripheral nervous system myelin maintenance	Gene Ontology	GO:0032287	2	0.038385
xenobiotic catabolic process	Gene Ontology	GO:0042178	2	0.038385
toxin transmembrane transporter activity	Gene Ontology	GO:0019534	2	0.038385
mast cell activation	Gene Ontology	GO:0045576	2	0.038385
ion homeostasis	Gene Ontology	GO:0050801	2	0.038385
endothelial cell apoptotic process	Gene Ontology	GO:0072577	2	0.038385
negative regulation of T cell apoptotic process	Gene Ontology	GO:0070233	2	0.038385
L-glutamine transmembrane transporter activity	Gene Ontology	GO:0015186	2	0.038385
hyaloid vascular plexus regression	Gene Ontology	GO:1990384	2	0.038385
external side of plasma membrane	Gene Ontology	GO:0009897	9	0.039087
sodium channel regulator activity	Gene Ontology	GO:0017080	3	0.039087
calcium ion transport	Gene Ontology	GO:0006816	4	0.040636
autophagy	Gene Ontology	GO:0006914	5	0.040648
positive regulation of intrinsic apoptotic signaling pathway	Gene Ontology	GO:2001244	3	0.041142
cell adhesion	Gene Ontology	GO:0007155	10	0.041889
negative regulation of protein binding	Gene Ontology	GO:0032091	4	0.041889
cellular response to nicotine	Gene Ontology	GO:0071316	2	0.041889
very-low-density lipoprotein particle remodeling	Gene Ontology	GO:0034372	2	0.041889
spanning component of membrane	Gene Ontology	GO:0089717	2	0.041889
positive regulation of cholesterol esterification	Gene Ontology	GO:0010873	2	0.041889
neutrophil degranulation	Gene Ontology	GO:0043312	10	0.041992
RNA polymerase II cis-regulatory region sequence-specific DNA binding	Gene Ontology	GO:0000978	12	0.04479
biosynthetic process	Gene Ontology	GO:0009058	2	0.047533
decanoate-CoA ligase activity	Gene Ontology	GO:0102391	2	0.047533
fructose metabolic process	Gene Ontology	GO:0006000	2	0.047533
IgG binding	Gene Ontology	GO:0019864	2	0.047533
positive regulation of mitochondrial outer membrane permeabilization involved in apoptotic signaling pathway	Gene Ontology	GO:1901030	2	0.047533
regulation of apoptotic process	Gene Ontology	GO:0042981	6	0.048427

#Term	Database	ID	Input number	Corrected P-Value
skeletal system development	Gene Ontology	GO:0001501	5	0.049864
Metabolic pathways	KEGG PATHWAY	hsa01100	36	3.58E-08
PPAR signaling pathway	KEGG PATHWAY	hsa03320	6	0.002705
Non-alcoholic fatty liver disease (NAFLD)	KEGG PATHWAY	hsa04932	7	0.007453
Fructose and mannose metabolism	KEGG PATHWAY	hsa00051	4	0.007782
Hepatitis C	KEGG PATHWAY	hsa05160	7	0.008779
Adipocytokine signaling pathway	KEGG PATHWAY	hsa04920	5	0.009671
PI3K-Akt signaling pathway	KEGG PATHWAY	hsa04151	10	0.013416
Fatty acid biosynthesis	KEGG PATHWAY	hsa00061	3	0.017085
@@Fatty acid metabolism	KEGG PATHWAY	hsa01212	4	0.027705
Glucagon signaling pathway	KEGG PATHWAY	hsa04922	5	0.030081
Human cytomegalovirus infection	KEGG PATHWAY	hsa05163	7	0.030812
Pathways in cancer	KEGG PATHWAY	hsa05200	11	0.034767
AMPK signaling pathway	KEGG PATHWAY	hsa04152	5	0.037346
Platinum drug resistance	KEGG PATHWAY	hsa01524	4	0.038385
Platelet activation	KEGG PATHWAY	hsa04611	5	0.039087
Biosynthesis of amino acids	KEGG PATHWAY	hsa01230	4	0.040636
Kaposi sarcoma-associated herpesvirus infection	KEGG PATHWAY	hsa05167	6	0.041343
Autophagy - animal	KEGG PATHWAY	hsa04140	5	0.041889
Tyrosine metabolism	KEGG PATHWAY	hsa00350	3	0.041889
Huntington disease	KEGG PATHWAY	hsa05016	6	0.045448
Apoptosis	KEGG PATHWAY	hsa04210	5	0.048813
Insulin signaling pathway	KEGG PATHWAY	hsa04910	5	0.049864
Metabolism	Reactome	R-HSA-1430728	48	6.76E-10
Immune System	Reactome	R-HSA-168256	42	5.53E-07
Metabolism of proteins	Reactome	R-HSA-392499	40	1.6E-06
Post-translational protein modification	Reactome	R-HSA-597592	31	1.01E-05
Innate Immune System	Reactome	R-HSA-168249	24	0.000137
Disease	Reactome	R-HSA-1643685	24	0.000142
Transport of small molecules	Reactome	R-HSA-382551	19	0.000247
Defective GALNT12 causes colorectal cancer 1 (CRCS1)	Reactome	R-HSA-5083636	4	0.001658
Metabolism of lipids	Reactome	R-HSA-556833	17	0.002619
Signaling by Receptor Tyrosine Kinases	Reactome	R-HSA-9006934	13	0.003273
Biological oxidations	Reactome	R-HSA-211859	9	0.00352
Signal Transduction	Reactome	R-HSA-162582	38	0.00352
Termination of O-glycan biosynthesis	Reactome	R-HSA-977068	4	0.00352
Fatty acid metabolism	Reactome	R-HSA-8978868	8	0.00352
Dectin-2 family	Reactome	R-HSA-5621480	4	0.00499
Metabolism of amino acids and derivatives	Reactome	R-HSA-71291	11	0.006097
C-type lectin receptors (CLRs)	Reactome	R-HSA-5621481	7	0.006147
Adaptive Immune System	Reactome	R-HSA-1280218	16	0.00713

#Term	Database	ID	Input number	Corrected P-Value
O-linked glycosylation of mucins	Reactome	R-HSA-913709	5	0.007339
Phase I - Functionalization of compounds	Reactome	R-HSA-211945	6	0.008035
Fatty acyl-CoA biosynthesis	Reactome	R-HSA-75105	4	0.010377
PPARA activates gene expression	Reactome	R-HSA-1989781	6	0.010957
Regulation of lipid metabolism by Peroxisome proliferator-activated receptor alpha (PPARalpha)	Reactome	R-HSA-400206	6	0.011133
Defective GALNT3 causes familial hyperphosphatemic tumoral calcinosis (HFTC)	Reactome	R-HSA-5083625	3	0.016336
Cytokine Signaling in Immune system	Reactome	R-HSA-1280215	16	0.016498
Defective C1GALT1C1 causes Tn polyagglutination syndrome (TNPS)	Reactome	R-HSA-5083632	3	0.017085
Ion channel transport	Reactome	R-HSA-983712	7	0.017329
Regulation of TLR by endogenous ligand	Reactome	R-HSA-5686938	3	0.018022
Interferon Signaling	Reactome	R-HSA-913531	7	0.020341
Diseases of glycosylation	Reactome	R-HSA-3781865	6	0.022964
Muscle contraction	Reactome	R-HSA-397014	7	0.027282
Synthesis of very long-chain fatty acyl-CoAs	Reactome	R-HSA-75876	3	0.027396
Toll-like Receptor Cascades	Reactome	R-HSA-168898	6	0.028246
Diseases of metabolism	Reactome	R-HSA-5668914	5	0.028246
Integration of energy metabolism	Reactome	R-HSA-163685	5	0.030587
Metabolism of carbohydrates	Reactome	R-HSA-71387	8	0.030812
Stimuli-sensing channels	Reactome	R-HSA-2672351	5	0.030812
Synthesis of substrates in N-glycan biosythesis	Reactome	R-HSA-446219	4	0.030812
O-linked glycosylation	Reactome	R-HSA-5173105	5	0.031406
Plasma lipoprotein assembly, remodeling, and clearance	Reactome	R-HSA-174824	4	0.031929
Class I MHC mediated antigen processing & presentation	Reactome	R-HSA-983169	9	0.033004
Infectious disease	Reactome	R-HSA-5663205	9	0.033773
Diseases associated with O-glycosylation of proteins	Reactome	R-HSA-3906995	4	0.034892
ISG15 antiviral mechanism	Reactome	R-HSA-1169408	4	0.034912
Circadian Clock	Reactome	R-HSA-400253	4	0.034912
Linoleic acid (LA) metabolism	Reactome	R-HSA-2046105	2	0.038385
SLC-mediated transmembrane transport	Reactome	R-HSA-425407	7	0.040128
Neutrophil degranulation	Reactome	R-HSA-6798695	10	0.041889
Antiviral mechanism by IFN-stimulated genes	Reactome	R-HSA-1169410	4	0.041889
Neuronal System	Reactome	R-HSA-112316	9	0.041889
Biosynthesis of the N-glycan precursor (dolichol lipid-linked oligosaccharide, LLO) and transfer to a nascent protein	Reactome	R-HSA-446193	4	0.042588
Formation of Fibrin Clot (Clotting Cascade)	Reactome	R-HSA-140877	3	0.048427
