# Supplementary material for: Transcriptome analysis reveals gene expression changes of pigs infected with non-lethal African swine fever virus
Source: Genet Mol Biol. 2023 Oct 13;46(3):e20230037. doi: 10.1590/1678-4685-GMB-2023-0037 (PMC10578457; doi:10.1590/1678-4685-GMB-2023-0037)
Supplement: Table S5 - [file 1415-4757-GMB-46-3-e20230037-s7.pdf]

Genetics and Molecular Biology

https://doi.org/10.1590/1678-4685-GMB-2023-0037

Supplementary Material to "Transcriptome analysis reveals gene expression changes of pigs infected with non-lethal African swine fever virus"

Table S5 - The enriched pathways of DEGs in MSLN using KOBAS 3.0.

#Term	Database	ID	Input number	Corrected P-Value
extracellular space	Gene Ontology	GO:0005615	6	0.002422
negative regulation of viral genome replication	Gene Ontology	GO:0045071	2	0.013483
type I interferon signaling pathway	Gene Ontology	GO:0060337	2	0.015223
defense response	Gene Ontology	GO:0006952	2	0.015223
Golgi lumen	Gene Ontology	GO:0005796	2	0.01823
response to virus	Gene Ontology	GO:0009615	2	0.01823
negative regulation of feeding behavior	Gene Ontology	GO:2000252	1	0.023895
CCR3 chemokine receptor binding	Gene Ontology	GO:0031728	1	0.023895
postsynaptic endocytic zone membrane	Gene Ontology	GO:0098844	1	0.023895
positive regulation of histone H3-K27 methylation	Gene Ontology	GO:0061087	1	0.023895
negative regulation of histone H3-K27 methylation	Gene Ontology	GO:0061086	1	0.023895
defense response to virus	Gene Ontology	GO:0051607	2	0.023895
positive regulation of ERK1 and ERK2 cascade	Gene Ontology	GO:0070374	2	0.023895
synaptic vesicle budding from presynaptic endocytic zone membrane	Gene Ontology	GO:0016185	1	0.023895
substrate-dependent cell migration	Gene Ontology	GO:0006929	1	0.023895
organelle fission	Gene Ontology	GO:0048285	1	0.023895
segment specification	Gene Ontology	GO:0007379	1	0.023895
positive regulation of immunoglobulin production	Gene Ontology	GO:0002639	1	0.023895
dynamin family protein polymerization involved in mitochondrial fission	Gene Ontology	GO:0003374	1	0.023895
response to type I interferon	Gene Ontology	GO:0034340	1	0.023895
tissue remodeling	Gene Ontology	GO:0048771	1	0.023895
postsynaptic neurotransmitter receptor internalization	Gene Ontology	GO:0098884	1	0.023895
extracellular region	Gene Ontology	GO:0005576	4	0.023895
plasma membrane	Gene Ontology	GO:0005886	6	0.023895
glial cell migration	Gene Ontology	GO:0008347	1	0.023895
ciliary neurotrophic factor receptor binding	Gene Ontology	GO:0005127	1	0.023895
granulocyte differentiation	Gene Ontology	GO:0030851	1	0.0247

#Term	Database	ID	Input number	Corrected P-Value
regulation of synapse structure or activity	Gene Ontology	GO:0050803	1	0.0247
zymogen granule membrane	Gene Ontology	GO:0042589	1	0.0247
positive regulation of astrocyte differentiation	Gene Ontology	GO:0048711	1	0.0247
T cell chemotaxis	Gene Ontology	GO:0010818	1	0.0247
dendritic spine head	Gene Ontology	GO:0044327	1	0.025103
dermatan sulfate biosynthetic process	Gene Ontology	GO:0030208	1	0.025103
signal transduction	Gene Ontology	GO:0007165	3	0.026171
ruffle assembly	Gene Ontology	GO:0097178	1	0.026171
chondroitin sulfate catabolic process	Gene Ontology	GO:0030207	1	0.026171
ESC/E(Z) complex	Gene Ontology	GO:0035098	1	0.026327
nucleus	Gene Ontology	GO:0005634	6	0.026327
intracellular calcium activated chloride channel activity	Gene Ontology	GO:0005229	1	0.026327
positive regulation of chemotaxis	Gene Ontology	GO:0050921	1	0.026327
positive regulation of synaptic transmission	Gene Ontology	GO:0050806	1	0.026327
eosinophil chemotaxis	Gene Ontology	GO:0048245	1	0.026327
proline-rich region binding	Gene Ontology	GO:0070064	1	0.026502
mitochondrial fission	Gene Ontology	GO:0000266	1	0.026662
inflammatory response	Gene Ontology	GO:0006954	2	0.027786
macrophage differentiation	Gene Ontology	GO:0030225	1	0.027786
zymogen activation	Gene Ontology	GO:0031638	1	0.027786
lamellipodium membrane	Gene Ontology	GO:0031258	1	0.027786
protein binding	Gene Ontology	GO:0005515	9	0.027956
focal adhesion	Gene Ontology	GO:0005925	2	0.030182
chondroitin sulfate biosynthetic process	Gene Ontology	GO:0030206	1	0.030182
negative regulation of JNK cascade	Gene Ontology	GO:0046329	1	0.030541
CCR chemokine receptor binding	Gene Ontology	GO:0048020	1	0.03125
platelet-derived growth factor receptor signaling pathway	Gene Ontology	GO:0048008	1	0.032168
negative regulation of gene expression, epigenetic	Gene Ontology	GO:0045814	1	0.032168
receptor signaling pathway via JAK-STAT	Gene Ontology	GO:0007259	1	0.032168
thyroid hormone receptor binding	Gene Ontology	GO:0046966	1	0.032168
stem cell differentiation	Gene Ontology	GO:0048863	1	0.032168
lymphocyte chemotaxis	Gene Ontology	GO:0048247	1	0.032803
receptor ligand activity	Gene Ontology	GO:0048018	1	0.035964
immune system process	Gene Ontology	GO:0002376	1	0.035964
positive regulation of cell population proliferation	Gene Ontology	GO:0008284	2	0.036153
membrane fusion	Gene Ontology	GO:0061025	1	0.036153
coreceptor activity	Gene Ontology	GO:0015026	1	0.038709
positive regulation of B cell proliferation	Gene Ontology	GO:0030890	1	0.038709
receptor internalization	Gene Ontology	GO:0031623	1	0.039224
cellular response to epidermal growth factor stimulus	Gene Ontology	GO:0071364	1	0.039335
monocyte chemotaxis	Gene Ontology	GO:0002548	1	0.039335
apoptotic process	Gene Ontology	GO:0006915	2	0.039597

#Term	Database	ID	Input number	Corrected P-Value
scavenger receptor activity	Gene Ontology	GO:0005044	1	0.040311
positive regulation of cell division	Gene Ontology	GO:0051781	1	0.041646
chemokine activity	Gene Ontology	GO:0008009	1	0.042102
epidermal growth factor receptor signaling pathway	Gene Ontology	GO:0007173	1	0.042627
cytoplasm	Gene Ontology	GO:0005737	5	0.042627
positive regulation of actin filament polymerization	Gene Ontology	GO:0030838	1	0.042627
stem cell population maintenance	Gene Ontology	GO:0019827	1	0.042627
cell surface	Gene Ontology	GO:0009986	2	0.043169
cellular defense response	Gene Ontology	GO:0006968	1	0.043462
response to mechanical stimulus	Gene Ontology	GO:0009612	1	0.043868
O-glycan processing	Gene Ontology	GO:0016266	1	0.04658
phagocytosis	Gene Ontology	GO:0006909	1	0.046943
methylated histone binding	Gene Ontology	GO:0035064	1	0.047301
specific granule lumen	Gene Ontology	GO:0035580	1	0.0476
metallopeptidase activity	Gene Ontology	GO:0008237	1	0.0476
chloride channel activity	Gene Ontology	GO:0005254	1	0.047884
response to insulin	Gene Ontology	GO:0032868	1	0.047884
chemokine-mediated signaling pathway	Gene Ontology	GO:0070098	1	0.048178
positive regulation of tyrosine phosphorylation of STAT protein	Gene Ontology	GO:0042531	1	0.048178
microvillus	Gene Ontology	GO:0005902	1	0.048178
fat cell differentiation	Gene Ontology	GO:0045444	1	0.048178
interferon-gamma-mediated signaling pathway	Gene Ontology	GO:0060333	1	0.048178
negative regulation of protein phosphorylation	Gene Ontology	GO:0001933	1	0.048178
double-stranded RNA binding	Gene Ontology	GO:0003725	1	0.048178
B cell differentiation	Gene Ontology	GO:0030183	1	0.048178
positive regulation of endothelial cell proliferation	Gene Ontology	GO:0001938	1	0.048178
calcium ion transport	Gene Ontology	GO:0006816	1	0.049108
Cytokine-cytokine receptor interaction	KEGG PATHWAY	hsa04060	2	0.025799
Human papillomavirus infection	KEGG PATHWAY	hsa05165	2	0.026327
Acute myeloid leukemia	KEGG PATHWAY	hsa05221	1	0.048178
Renin secretion	KEGG PATHWAY	hsa04924	1	0.048178
Interferon alpha/beta signaling	Reactome	R-HSA-909733	2	0.015223
Antiviral mechanism by IFN-stimulated genes	Reactome	R-HSA-1169410	2	0.015223
Immune System	Reactome	R-HSA-168256	5	0.01823
Diseases of glycosylation	Reactome	R-HSA-3781865	2	0.023895
Interferon Signaling	Reactome	R-HSA-913531	2	0.023895
Cytokine Signaling in Immune system	Reactome	R-HSA-1280215	3	0.023895
Defective CHST3 causes SEDCJD	Reactome	R-HSA-3595172	1	0.023895
Defective CHST14 causes EDS, musculocontractural type	Reactome	R-HSA-3595174	1	0.023895
Defective CHSY1 causes TPBS	Reactome	R-HSA-3595177	1	0.023895
OAS antiviral response	Reactome	R-HSA-8983711	1	0.023895

#Term	Database	ID	Input number	Corrected P-Value
Dermatan sulfate biosynthesis	Reactome	R-HSA-2022923	1	0.0247
CS/DS degradation	Reactome	R-HSA-2024101	1	0.026171
Defective GALNT12 causes colorectal cancer 1 (CRCS1)	Reactome	R-HSA-5083636	1	0.026327
IL-6-type cytokine receptor ligand interactions	Reactome	R-HSA-6788467	1	0.026327
Defective GALNT3 causes familial hyperphosphatemic tumoral calcinosis (HFTC)	Reactome	R-HSA-5083625	1	0.026327
Defective C1GALT1C1 causes Tn polyagglutination syndrome (TNPS)	Reactome	R-HSA-5083632	1	0.026502
Chondroitin sulfate biosynthesis	Reactome	R-HSA-2022870	1	0.026662
Defective B3GALT6 causes EDSP2 and SEMDJL1	Reactome	R-HSA-4420332	1	0.026808
Defective B4GALT7 causes EDS, progeroid type	Reactome	R-HSA-3560783	1	0.026808
Defective B3GAT3 causes JDSSDHD	Reactome	R-HSA-3560801	1	0.026808
Termination of O-glycan biosynthesis	Reactome	R-HSA-977068	1	0.029031
Interleukin-6 family signaling	Reactome	R-HSA-6783589	1	0.029031
A tetrasaccharide linker sequence is required for GAG synthesis	Reactome	R-HSA-1971475	1	0.030182
Dectin-2 family	Reactome	R-HSA-5621480	1	0.030541
FOXO-mediated transcription of oxidative stress, metabolic and neuronal genes	Reactome	R-HSA-9615017	1	0.031942
Diseases associated with glycosaminoglycan metabolism	Reactome	R-HSA-3560782	1	0.038709
Chondroitin sulfate/dermatan sulfate metabolism	Reactome	R-HSA-1793185	1	0.042548
Heparan sulfate/heparin (HS-GAG) metabolism	Reactome	R-HSA-1638091	1	0.044266
O-linked glycosylation of mucins	Reactome	R-HSA-913709	1	0.0476
FOXO-mediated transcription	Reactome	R-HSA-9614085	1	0.047884
Diseases associated with O-glycosylation of proteins	Reactome	R-HSA-3906995	1	0.048178
ISG15 antiviral mechanism	Reactome	R-HSA-1169408	1	0.048178
PRC2 methylates histones and DNA	Reactome	R-HSA-212300	1	0.048178
