# Supplementary material for: Transcriptome analysis reveals gene expression changes of pigs infected with non-lethal African swine fever virus
Source: Genet Mol Biol. 2023 Oct 13;46(3):e20230037. doi: 10.1590/1678-4685-GMB-2023-0037 (PMC10578457; doi:10.1590/1678-4685-GMB-2023-0037)
Supplement: Table S6 - [file 1415-4757-GMB-46-3-e20230037-s8.pdf]

Genetics and Molecular Biology

https://doi.org/10.1590/1678-4685-GMB-2023-0037

Supplementary Material to "Transcriptome analysis reveals gene expression changes of pigs infected with non-lethal African swine fever virus"

Table S6 - The enriched pathways of DEGs in PBMC using KOBAS 3.0.

#Term	Database	ID	Input number	Corrected P-Value
protein binding	Gene Ontology	GO:0005515	243	2.22E-49
cytoplasm	Gene Ontology	GO:0005737	107	7.21E-18
nucleus	Gene Ontology	GO:0005634	114	1.06E-17
cytosol	Gene Ontology	GO:0005829	102	8.39E-13
plasma membrane	Gene Ontology	GO:0005886	91	1.08E-10
positive regulation of transcription by RNA polymerase II	Gene Ontology	GO:0045944	39	1.41E-09
nucleoplasm	Gene Ontology	GO:0005654	75	2.18E-09
DNA-binding transcription factor activity, RNA polymerase II-specific	Gene Ontology	GO:0000981	32	1.44E-08
negative regulation of transcription by RNA polymerase II	Gene Ontology	GO:0000122	30	7.73E-08
RNA polymerase II cis-regulatory region sequence-specific DNA binding	Gene Ontology	GO:0000978	26	1.77E-07
membrane	Gene Ontology	GO:0016020	48	5.97E-07
nuclear chromatin	Gene Ontology	GO:0000790	29	1.95E-06
cell adhesion	Gene Ontology	GO:0007155	20	6.11E-06
positive regulation of transcription, DNA-templated	Gene Ontology	GO:0045893	20	5.19E-05
integral component of membrane	Gene Ontology	GO:0016021	63	5.19E-05
hormone-mediated signaling pathway	Gene Ontology	GO:0009755	7	8.49E-05
Golgi apparatus	Gene Ontology	GO:0005794	27	0.000101
regulation of cell population proliferation	Gene Ontology	GO:0042127	11	0.000117
endoplasmic reticulum	Gene Ontology	GO:0005783	27	0.000122
chromatin binding	Gene Ontology	GO:0003682	17	0.000138
DNA-binding transcription factor activity	Gene Ontology	GO:0003700	20	0.000141
sequence-specific double-stranded DNA binding	Gene Ontology	GO:1990837	19	0.000141
protein C-terminus binding	Gene Ontology	GO:0008022	11	0.00026
negative regulation of transcription, DNA-templated	Gene Ontology	GO:0045892	18	0.000342
identical protein binding	Gene Ontology	GO:0042802	32	0.000446
ATP binding	Gene Ontology	GO:0005524	32	0.000473
metal ion binding	Gene Ontology	GO:0046872	43	0.000476
negative regulation of cell population proliferation	Gene Ontology	GO:0008285	15	0.000476

#Term	Database	ID	Input number	Corrected P-Value
positive regulation of cell population proliferation	Gene Ontology	GO:0008284	17	0.000476
DNA-binding transcription repressor activity, RNA polymerase II-specific	Gene Ontology	GO:0001227	12	0.000517
protein-containing complex	Gene Ontology	GO:0032991	19	0.00054
DNA binding	Gene Ontology	GO:0003677	28	0.000568
signal transduction	Gene Ontology	GO:0007165	25	0.000621
positive regulation of p38MAPK cascade	Gene Ontology	GO:1900745	5	0.000699
cellular response to vascular endothelial growth factor stimulus	Gene Ontology	GO:0035924	5	0.000801
negative regulation of NF-kappaB transcription factor activity	Gene Ontology	GO:0032088	7	0.001037
inflammatory response	Gene Ontology	GO:0006954	14	0.001037
vesicle-mediated transport	Gene Ontology	GO:0016192	9	0.001037
protein localization to plasma membrane	Gene Ontology	GO:0072659	9	0.00112
angiogenesis	Gene Ontology	GO:0001525	11	0.001137
positive regulation of apoptotic process	Gene Ontology	GO:0043065	13	0.001137
osteoclast differentiation	Gene Ontology	GO:0030316	5	0.001172
integral component of plasma membrane	Gene Ontology	GO:0005887	29	0.001562
signaling receptor binding	Gene Ontology	GO:0005102	13	0.001733
DNA-binding transcription activator activity, RNA polymerase II-specific	Gene Ontology	GO:0001228	15	0.001829
basolateral plasma membrane	Gene Ontology	GO:0016323	10	0.00224
cell differentiation	Gene Ontology	GO:0030154	17	0.00224
protein homodimerization activity	Gene Ontology	GO:0042803	18	0.00224
negative regulation of vascular endothelial cell proliferation	Gene Ontology	GO:1905563	3	0.00224
trans-Golgi network	Gene Ontology	GO:0005802	9	0.00224
retinoid X receptor binding	Gene Ontology	GO:0046965	4	0.00224
positive regulation of cold-induced thermogenesis	Gene Ontology	GO:0120162	7	0.00224
positive regulation of nitric oxide biosynthetic process	Gene Ontology	GO:0045429	5	0.002365
late endosome	Gene Ontology	GO:0005770	8	0.002839
protein dimerization activity	Gene Ontology	GO:0046983	8	0.002839
blood vessel morphogenesis	Gene Ontology	GO:0048514	4	0.002855
UDP-N-acetylglucosamine metabolic process	Gene Ontology	GO:0006047	3	0.002855
extracellular region	Gene Ontology	GO:0005576	34	0.002855
nuclear receptor activity	Gene Ontology	GO:0004879	5	0.002855
regulation of transcription by RNA polymerase II	Gene Ontology	GO:0006357	19	0.002867
brain development	Gene Ontology	GO:0007420	10	0.002867
molecular adaptor activity	Gene Ontology	GO:0060090	6	0.002886
regulation of insulin secretion	Gene Ontology	GO:0050796	6	0.002886
regulation of gene expression	Gene Ontology	GO:0010468	10	0.003077
Wnt signaling pathway	Gene Ontology	GO:0016055	9	0.00309
membrane raft	Gene Ontology	GO:0045121	10	0.003187
calmodulin-dependent protein kinase activity	Gene Ontology	GO:0004683	4	0.003238
vascular endothelial growth factor production	Gene Ontology	GO:0010573	3	0.003297

#Term	Database	ID	Input number	Corrected P-Value
non-membrane spanning protein tyrosine kinase activity	Gene Ontology	GO:0004715	5	0.003508
regulation of transcription, DNA-templated	Gene Ontology	GO:0006355	19	0.003508
extracellular space	Gene Ontology	GO:0005615	30	0.003533
positive regulation of DNA-binding transcription factor activity	Gene Ontology	GO:0051091	7	0.003641
response to hydrogen peroxide	Gene Ontology	GO:0042542	5	0.004037
glutamatergic synapse	Gene Ontology	GO:0098978	12	0.004115
positive regulation of NF-kappaB transcription factor activity	Gene Ontology	GO:0051092	8	0.004478
transcription regulatory region sequence-specific DNA binding	Gene Ontology	GO:0000976	10	0.005949
innate immune response	Gene Ontology	GO:0045087	15	0.006469
protein autophosphorylation	Gene Ontology	GO:0046777	8	0.006469
actin cytoskeleton reorganization	Gene Ontology	GO:0031532	5	0.007285
negative regulation of angiogenesis	Gene Ontology	GO:0016525	6	0.007285
exocytosis	Gene Ontology	GO:0006887	6	0.007992
positive regulation of protein phosphorylation	Gene Ontology	GO:0001934	8	0.008166
positive regulation of cell migration	Gene Ontology	GO:0030335	9	0.008207
positive regulation of gene expression	Gene Ontology	GO:0010628	12	0.008207
response to glucocorticoid	Gene Ontology	GO:0051384	5	0.008496
cytokine-mediated signaling pathway	Gene Ontology	GO:0019221	10	0.008542
PERK-mediated unfolded protein response	Gene Ontology	GO:0036499	3	0.008542
inorganic anion exchanger activity	Gene Ontology	GO:0005452	3	0.008542
activation of MAPKKK activity	Gene Ontology	GO:0000185	3	0.008542
regulation of neurogenesis	Gene Ontology	GO:0050767	4	0.009138
regulation of cell differentiation	Gene Ontology	GO:0045595	4	0.009893
regulation of postsynaptic neurotransmitter receptor activity	Gene Ontology	GO:0098962	3	0.009904
DNA-binding transcription factor inhibitor activity	Gene Ontology	GO:0140416	3	0.009904
G1/S transition of mitotic cell cycle	Gene Ontology	GO:0000082	6	0.010082
cell surface	Gene Ontology	GO:0009986	15	0.010742
peptidyl-tyrosine autophosphorylation	Gene Ontology	GO:0038083	4	0.01131
secondary palate development	Gene Ontology	GO:0062009	3	0.01131
focal adhesion	Gene Ontology	GO:0005925	12	0.01235
regulation of cell shape	Gene Ontology	GO:0008360	7	0.0128
dendritic spine development	Gene Ontology	GO:0060996	3	0.0128
positive regulation of membrane protein ectodomain proteolysis	Gene Ontology	GO:0051044	3	0.0128
negative regulation of lipid catabolic process	Gene Ontology	GO:0050995	3	0.0128
extracellular exosome	Gene Ontology	GO:0070062	34	0.013117
fat cell differentiation	Gene Ontology	GO:0045444	5	0.013117
canonical Wnt signaling pathway	Gene Ontology	GO:0060070	5	0.013117
negative regulation of apoptotic process	Gene Ontology	GO:0043066	13	0.013117
nuclear body	Gene Ontology	GO:0016604	10	0.013203
intracellular signal transduction	Gene Ontology	GO:0035556	11	0.014275
positive regulation of heterotypic cell-cell adhesion	Gene Ontology	GO:0034116	3	0.014275
calcium ion binding	Gene Ontology	GO:0005509	16	0.014285

#Term	Database	ID	Input number	Corrected P-Value
positive regulation of pri-miRNA transcription by RNA polymerase II	Gene Ontology	GO:1902895	4	0.014296
centrosome cycle	Gene Ontology	GO:0007098	4	0.014296
nucleolus	Gene Ontology	GO:0005730	18	0.014897
visual perception	Gene Ontology	GO:0007601	8	0.014913
GTPase activity	Gene Ontology	GO:0003924	10	0.016488
cellular response to organic cyclic compound	Gene Ontology	GO:0071407	4	0.020177
protein-membrane adaptor activity	Gene Ontology	GO:0043495	3	0.020177
negative regulation of protein kinase B signaling	Gene Ontology	GO:0051898	4	0.021408
peptidyl-tyrosine phosphorylation	Gene Ontology	GO:0018108	6	0.02236
neuron projection	Gene Ontology	GO:0043005	10	0.02236
nuclear receptor binding	Gene Ontology	GO:0016922	3	0.02236
negative regulation of cell growth	Gene Ontology	GO:0030308	6	0.022781
cell migration involved in sprouting angiogenesis	Gene Ontology	GO:0002042	3	0.024083
negative regulation of blood vessel endothelial cell migration	Gene Ontology	GO:0043537	3	0.024083
anion:anion antiporter activity	Gene Ontology	GO:0015301	3	0.024083
sequence-specific DNA binding	Gene Ontology	GO:0043565	11	0.024609
cytoplasmic vesicle	Gene Ontology	GO:0031410	9	0.024873
beta-catenin binding	Gene Ontology	GO:0008013	5	0.025038
ubiquitin protein ligase activity	Gene Ontology	GO:0061630	9	0.025038
zinc ion binding	Gene Ontology	GO:0008270	17	0.025039
positive regulation of neural precursor cell proliferation	Gene Ontology	GO:2000179	3	0.025722
negative regulation of neurogenesis	Gene Ontology	GO:0050768	3	0.025722
positive regulation of angiogenesis	Gene Ontology	GO:0045766	6	0.026061
viral entry into host cell	Gene Ontology	GO:0046718	5	0.027475
nuclear receptor transcription coactivator activity	Gene Ontology	GO:0030374	4	0.027549
regulation of mitotic nuclear division	Gene Ontology	GO:0007088	3	0.027549
negative regulation of T cell receptor signaling pathway	Gene Ontology	GO:0050860	3	0.027549
extrinsic component of plasma membrane	Gene Ontology	GO:0019897	3	0.027549
steroid hormone mediated signaling pathway	Gene Ontology	GO:0043401	3	0.027549
positive regulation of DNA demethylation	Gene Ontology	GO:1901537	2	0.027794
semicircular canal morphogenesis	Gene Ontology	GO:0048752	2	0.027794
hemoglobin binding	Gene Ontology	GO:0030492	2	0.027794
positive regulation of fatty acid oxidation	Gene Ontology	GO:0046321	2	0.027794
paraxial mesoderm formation	Gene Ontology	GO:0048341	2	0.027794
negative regulation of apoptotic process in bone marrow cell	Gene Ontology	GO:0071866	2	0.027794
positive regulation of fever generation	Gene Ontology	GO:0031622	2	0.027794
cellular response to cytokine stimulus	Gene Ontology	GO:0071345	4	0.027933
protein serine/threonine kinase activity	Gene Ontology	GO:0004674	10	0.02798
regulation of lipid metabolic process	Gene Ontology	GO:0019216	5	0.028558
secretory granule	Gene Ontology	GO:0030141	5	0.028558
cis-regulatory region sequence-specific DNA binding	Gene Ontology	GO:0000987	4	0.0304

#Term	Database	ID	Input number	Corrected P-Value
exocytic vesicle	Gene Ontology	GO:0070382	3	0.03121
transcription coactivator activity	Gene Ontology	GO:0003713	8	0.032862
nucleosome binding	Gene Ontology	GO:0031491	3	0.032862
membrane raft assembly	Gene Ontology	GO:0001765	2	0.032862
positive regulation of feeding behavior	Gene Ontology	GO:2000253	2	0.032862
T-helper 1 cell differentiation	Gene Ontology	GO:0045063	2	0.032862
lung vasculature development	Gene Ontology	GO:0060426	2	0.032862
tooth eruption	Gene Ontology	GO:0044691	2	0.032862
regulation of atrial cardiac muscle cell membrane repolarization	Gene Ontology	GO:0060372	2	0.032862
negative regulation of meiotic nuclear division	Gene Ontology	GO:0045835	2	0.032862
phosphatidylserine binding	Gene Ontology	GO:0001786	4	0.033944
promoter-specific chromatin binding	Gene Ontology	GO:1990841	4	0.033944
GTP binding	Gene Ontology	GO:0005525	10	0.034171
cellular response to fibroblast growth factor stimulus	Gene Ontology	GO:0044344	3	0.035056
protein localization to cell surface	Gene Ontology	GO:0034394	3	0.035056
positive regulation of epithelial cell proliferation	Gene Ontology	GO:0050679	4	0.035226
peptide binding	Gene Ontology	GO:0042277	4	0.038436
positive regulation of MAP kinase activity	Gene Ontology	GO:0043406	4	0.038436
L-serine transport	Gene Ontology	GO:0015825	2	0.038436
prostate gland epithelium morphogenesis	Gene Ontology	GO:0060740	2	0.038436
L-serine transmembrane transporter activity	Gene Ontology	GO:0015194	2	0.038436
calcium-dependent protein serine/threonine kinase activity	Gene Ontology	GO:0009931	2	0.038436
regulation of type B pancreatic cell proliferation	Gene Ontology	GO:0061469	2	0.038436
positive regulation of neuroinflammatory response	Gene Ontology	GO:0150078	2	0.038436
negative regulation of interleukin-4 production	Gene Ontology	GO:0032713	2	0.038436
hyaluronan biosynthetic process	Gene Ontology	GO:0030213	2	0.038436
ciliary basal body	Gene Ontology	GO:0036064	6	0.038861
regulation of cell motility	Gene Ontology	GO:2000145	3	0.039079
embryonic hindlimb morphogenesis	Gene Ontology	GO:0035116	3	0.039079
branching involved in blood vessel morphogenesis	Gene Ontology	GO:0001569	3	0.039079
protein N-terminus binding	Gene Ontology	GO:0047485	5	0.039292
transcription factor binding	Gene Ontology	GO:0008134	9	0.041046
negative regulation of DNA binding	Gene Ontology	GO:0043392	3	0.042037
RNA polymerase II transcription regulatory region sequence-specific DNA binding	Gene Ontology	GO:0000977	9	0.042037
hippocampus development	Gene Ontology	GO:0021766	4	0.042037
negative regulation of gene expression	Gene Ontology	GO:0010629	7	0.043219
embryonic skeletal system development	Gene Ontology	GO:0048706	3	0.043219
Golgi to plasma membrane protein transport	Gene Ontology	GO:0043001	3	0.043219
endothelial cell chemotaxis	Gene Ontology	GO:0035767	2	0.043219
regulation of establishment of endothelial barrier	Gene Ontology	GO:1903140	2	0.043219
negative regulation of synaptic transmission	Gene Ontology	GO:0050805	2	0.043219

#Term	Database	ID	Input number	Corrected P-Value
transcription regulator activity	Gene Ontology	GO:0140110	2	0.043219
heterochromatin organization	Gene Ontology	GO:0070828	2	0.043219
anaphase-promoting complex binding	Gene Ontology	GO:0010997	2	0.043219
cilium	Gene Ontology	GO:0005929	7	0.043407
positive regulation of NIK/NF-kappaB signaling	Gene Ontology	GO:1901224	4	0.043534
circadian regulation of gene expression	Gene Ontology	GO:0032922	4	0.043534
integral component of endoplasmic reticulum membrane	Gene Ontology	GO:0030176	5	0.043534
cellular response to retinoic acid	Gene Ontology	GO:0071300	4	0.04502
response to hypoxia	Gene Ontology	GO:0001666	6	0.04502
Rho GTPase binding	Gene Ontology	GO:0017048	3	0.04502
blood vessel remodeling	Gene Ontology	GO:0001974	3	0.04502
positive regulation of neuron projection development	Gene Ontology	GO:0010976	5	0.045903
peptidyl-serine phosphorylation	Gene Ontology	GO:0018105	6	0.045999
positive regulation of tyrosine phosphorylation of STAT protein	Gene Ontology	GO:0042531	4	0.046782
dendritic spine	Gene Ontology	GO:0043197	6	0.046952
protein localization to cilium	Gene Ontology	GO:0061512	3	0.047452
odontogenesis	Gene Ontology	GO:0042476	3	0.047452
Rab guanyl-nucleotide exchange factor activity	Gene Ontology	GO:0017112	3	0.047452
histone binding	Gene Ontology	GO:0042393	6	0.047578
protein autoubiquitination	Gene Ontology	GO:0051865	4	0.047952
carbohydrate biosynthetic process	Gene Ontology	GO:0016051	2	0.047952
tyrosine phosphorylation of STAT protein	Gene Ontology	GO:0007260	2	0.047952
atrial cardiac muscle cell action potential	Gene Ontology	GO:0086014	2	0.047952
regulation of smooth muscle cell proliferation	Gene Ontology	GO:0048660	2	0.047952
aryl hydrocarbon receptor binding	Gene Ontology	GO:0017162	2	0.047952
negative regulation of ERK1 and ERK2 cascade	Gene Ontology	GO:0070373	4	0.048785
semaphorin-plexin signaling pathway	Gene Ontology	GO:0071526	3	0.048785
positive regulation of vascular associated smooth muscle cell proliferation	Gene Ontology	GO:1904707	3	0.048785
blood circulation	Gene Ontology	GO:0008015	3	0.048785
intrinsic apoptotic signaling pathway in response to endoplasmic reticulum stress	Gene Ontology	GO:0070059	3	0.048785
lipopolysaccharide-mediated signaling pathway	Gene Ontology	GO:0031663	3	0.048785
response to bacterium	Gene Ontology	GO:0009617	5	0.049043
Malaria	KEGG PATHWAY	hsa05144	6	0.000818
Fluid shear stress and atherosclerosis	KEGG PATHWAY	hsa05418	8	0.002792
MAPK signaling pathway	KEGG PATHWAY	hsa04010	11	0.003676
Pathways in cancer	KEGG PATHWAY	hsa05200	15	0.004147
Proteoglycans in cancer	KEGG PATHWAY	hsa05205	9	0.004341
Oocyte meiosis	KEGG PATHWAY	hsa04114	7	0.006761
Rheumatoid arthritis	KEGG PATHWAY	hsa05323	6	0.007285
Aldosterone synthesis and secretion	KEGG PATHWAY	hsa04925	6	0.009479

#Term	Database	ID	Input number	Corrected P-Value
Signaling pathways regulating pluripotency of stem cells	KEGG PATHWAY	hsa04550	7	0.009777
Cholinergic synapse	KEGG PATHWAY	hsa04725	6	0.014872
Gastric acid secretion	KEGG PATHWAY	hsa04971	5	0.016203
Metabolic pathways	KEGG PATHWAY	hsa01100	25	0.024083
ECM-receptor interaction	KEGG PATHWAY	hsa04512	5	0.025038
Tuberculosis	KEGG PATHWAY	hsa05152	7	0.025722
Axon guidance	KEGG PATHWAY	hsa04360	7	0.027003
Transcriptional misregulation in cancer	KEGG PATHWAY	hsa05202	7	0.028476
Estrogen signaling pathway	KEGG PATHWAY	hsa04915	6	0.02975
AGE-RAGE signaling pathway in diabetic complications	KEGG PATHWAY	hsa04933	5	0.036118
Melanogenesis	KEGG PATHWAY	hsa04916	5	0.03732
C-type lectin receptor signaling pathway	KEGG PATHWAY	hsa04625	5	0.039292
Cushing syndrome	KEGG PATHWAY	hsa04934	6	0.042041
Human papillomavirus infection	KEGG PATHWAY	hsa05165	9	0.043219
TNF signaling pathway	KEGG PATHWAY	hsa04668	5	0.046952
Signal Transduction	Reactome	R-HSA-162582	52	3.01E-05
Signaling by Receptor Tyrosine Kinases	Reactome	R-HSA-9006934	16	0.000568
Metabolism of proteins	Reactome	R-HSA-392499	38	0.001037
Developmental Biology	Reactome	R-HSA-1266738	25	0.001365
Gene expression (Transcription)	Reactome	R-HSA-74160	30	0.001486
Cytokine Signaling in Immune system	Reactome	R-HSA-1280215	21	0.001829
Generic Transcription Pathway	Reactome	R-HSA-212436	26	0.00216
FOXO-mediated transcription	Reactome	R-HSA-9614085	6	0.00224
Signaling by Interleukins	Reactome	R-HSA-449147	17	0.002855
DAG and IP3 signaling	Reactome	R-HSA-1489509	5	0.002855
RNA Polymerase II Transcription	Reactome	R-HSA-73857	27	0.002891
Signaling by VEGF	Reactome	R-HSA-194138	7	0.003
Interleukin-4 and Interleukin-13 signaling	Reactome	R-HSA-6785807	7	0.003187
Disease	Reactome	R-HSA-1643685	23	0.003508
Transport of small molecules	Reactome	R-HSA-382551	18	0.004115
Transcriptional regulation of white adipocyte differentiation	Reactome	R-HSA-381340	6	0.00501
Hemostasis	Reactome	R-HSA-109582	16	0.006073
PLC beta mediated events	Reactome	R-HSA-112043	5	0.006073
G-protein mediated events	Reactome	R-HSA-112040	5	0.006469
Metabolism	Reactome	R-HSA-1430728	35	0.007768
Immune System	Reactome	R-HSA-168256	35	0.008542
Calmodulin induced events	Reactome	R-HSA-111933	4	0.01131
CaM pathway	Reactome	R-HSA-111997	4	0.01131
SEMA3A-Plexin repulsion signaling by inhibiting Integrin adhesion	Reactome	R-HSA-399955	3	0.01131
Post-translational protein modification	Reactome	R-HSA-597592	26	0.011769
Circadian Clock	Reactome	R-HSA-400253	5	0.0128
Ca-dependent events	Reactome	R-HSA-111996	4	0.01281

#Term	Database	ID	Input number	Corrected P-Value
PPARA activates gene expression	Reactome	R-HSA-1989781	6	0.015826
Regulation of lipid metabolism by Peroxisome proliferator-activated receptor alpha (PPARalpha)	Reactome	R-HSA-400206	6	0.01632
VEGFR2 mediated cell proliferation	Reactome	R-HSA-5218921	3	0.020177
Rab regulation of trafficking	Reactome	R-HSA-9007101	6	0.021812
Interleukin-10 signaling	Reactome	R-HSA-6783783	4	0.022381
Cell-Cell communication	Reactome	R-HSA-1500931	6	0.022781
Neuronal System	Reactome	R-HSA-112316	11	0.02405
Intracellular signaling by second messengers	Reactome	R-HSA-9006925	9	0.025722
RAB GEFs exchange GTP for GDP on RABs	Reactome	R-HSA-8876198	5	0.027475
Cell surface interactions at the vascular wall	Reactome	R-HSA-202733	6	0.027794
Opioid Signalling	Reactome	R-HSA-111885	5	0.027947
Unfolded Protein Response (UPR)	Reactome	R-HSA-381119	5	0.028558
Signaling by PDGF	Reactome	R-HSA-186797	4	0.0304
SLC-mediated transmembrane transport	Reactome	R-HSA-425407	8	0.032862
ATF4 activates genes in response to endoplasmic reticulum stress	Reactome	R-HSA-380994	3	0.032862
Phosphorylation of Emi1	Reactome	R-HSA-176417	2	0.032862
Uptake and actions of bacterial toxins	Reactome	R-HSA-5339562	3	0.035056
Signaling by RAS mutants	Reactome	R-HSA-6802949	4	0.037002
Ca2+ pathway	Reactome	R-HSA-4086398	4	0.038436
Semaphorin interactions	Reactome	R-HSA-373755	4	0.042037
Vesicle-mediated transport	Reactome	R-HSA-5653656	14	0.042041
PERK regulates gene expression	Reactome	R-HSA-381042	3	0.043219
VEGF ligand-receptor interactions	Reactome	R-HSA-194313	2	0.043219
VEGF binds to VEGFR leading to receptor dimerization	Reactome	R-HSA-195399	2	0.043219
RAF activation	Reactome	R-HSA-5673000	3	0.04502
Erythrocytes take up oxygen and release carbon dioxide	Reactome	R-HSA-1247673	2	0.047952
