# Supplementary material for: Transcriptome analysis reveals gene expression changes of pigs infected with non-lethal African swine fever virus
Source: Genet Mol Biol. 2023 Oct 13;46(3):e20230037. doi: 10.1590/1678-4685-GMB-2023-0037 (PMC10578457; doi:10.1590/1678-4685-GMB-2023-0037)
Supplement: Table S7 - [file 1415-4757-GMB-46-3-e20230037-s9.pdf]

Genetics and Molecular Biology

https://doi.org/10.1590/1678-4685-GMB-2023-0037

Supplementary Material to "Transcriptome analysis reveals gene expression changes of pigs infected with non-lethal African swine fever virus"

Table S7 - The enriched pathways of DEGs in SMLN using KOBAS 3.0.

#Term	Database	ID	Input number	Corrected P-Value
protein binding	Gene Ontology	GO:0005515	233	1.87E-32
plasma membrane	Gene Ontology	GO:0005886	118	5.04E-20
cytosol	Gene Ontology	GO:0005829	115	2.24E-15
extracellular exosome	Gene Ontology	GO:0070062	68	3.45E-15
cytoplasm	Gene Ontology	GO:0005737	105	5.02E-14
integral component of membrane	Gene Ontology	GO:0016021	81	1.02E-09
apical plasma membrane	Gene Ontology	GO:0016324	23	1.49E-09
postsynaptic density	Gene Ontology	GO:0014069	19	9.16E-09
positive regulation of transcription by RNA polymerase II	Gene Ontology	GO:0045944	39	1.54E-08
extracellular space	Gene Ontology	GO:0005615	46	2.07E-08
nucleus	Gene Ontology	GO:0005634	97	4.93E-08
integral component of plasma membrane	Gene Ontology	GO:0005887	41	1.37E-07
wound healing	Gene Ontology	GO:0042060	11	9.73E-07
DNA-binding transcription factor activity, RNA polymerase II-specific	Gene Ontology	GO:0000981	30	1.32E-06
actin cytoskeleton	Gene Ontology	GO:0015629	16	1.45E-06
lamellipodium	Gene Ontology	GO:0030027	14	2.15E-06
identical protein binding	Gene Ontology	GO:0042802	39	4.21E-06
ATPase binding	Gene Ontology	GO:0051117	10	7.31E-06
basal plasma membrane	Gene Ontology	GO:0009925	8	1.51E-05
cell migration	Gene Ontology	GO:0016477	14	2.26E-05
nuclear chromatin	Gene Ontology	GO:0000790	28	2.7E-05
membrane	Gene Ontology	GO:0016020	46	3.6E-05
regulation of small GTPase mediated signal transduction	Gene Ontology	GO:0051056	11	3.6E-05
actin filament binding	Gene Ontology	GO:0051015	13	3.7E-05
focal adhesion	Gene Ontology	GO:0005925	18	3.97E-05
negative regulation of transcription by RNA polymerase II	Gene Ontology	GO:0000122	26	4.24E-05
actin binding	Gene Ontology	GO:0003779	14	7.79E-05
metal ion binding	Gene Ontology	GO:0046872	48	7.83E-05
glutamatergic synapse	Gene Ontology	GO:0098978	16	8.16E-05

#Term	Database	ID	Input number	Corrected P-Value
cell surface	Gene Ontology	GO:0009986	21	8.29E-05
RNA polymerase II cis-regulatory region sequence-specific DNA binding	Gene Ontology	GO:0000978	22	9.1E-05
cytoskeleton	Gene Ontology	GO:0005856	17	9.1E-05
Rho guanyl-nucleotide exchange factor activity	Gene Ontology	GO:0005089	7	9.1E-05
dendritic spine	Gene Ontology	GO:0043197	11	9.47E-05
filopodium	Gene Ontology	GO:0030175	8	9.49E-05
signal transduction	Gene Ontology	GO:0007165	28	0.000106
collagen-containing extracellular matrix	Gene Ontology	GO:0062023	16	0.00011
DNA-binding transcription activator activity, RNA polymerase II-specific	Gene Ontology	GO:0001228	18	0.000111
cortical actin cytoskeleton	Gene Ontology	GO:0030864	7	0.000145
apical dendrite	Gene Ontology	GO:0097440	5	0.000146
nucleoplasm	Gene Ontology	GO:0005654	64	0.000202
ureteric bud development	Gene Ontology	GO:0001657	6	0.00025
sequence-specific double-stranded DNA binding	Gene Ontology	GO:1990837	19	0.000254
positive regulation of transcription, DNA-templated	Gene Ontology	GO:0045893	19	0.000262
GTPase activator activity	Gene Ontology	GO:0005096	13	0.000262
adherens junction	Gene Ontology	GO:0005912	10	0.000324
transcription initiation from RNA polymerase II promoter	Gene Ontology	GO:0006367	10	0.000414
transcription coactivator binding	Gene Ontology	GO:0001223	5	0.000414
negative regulation of apoptotic process	Gene Ontology	GO:0043066	17	0.000588
ATP binding	Gene Ontology	GO:0005524	33	0.000588
negative regulation of stress fiber assembly	Gene Ontology	GO:0051497	5	0.000666
DNA-binding transcription factor activity	Gene Ontology	GO:0003700	19	0.000737
hippo signaling	Gene Ontology	GO:0035329	5	0.000747
postsynaptic membrane	Gene Ontology	GO:0045211	9	0.000877
cell-cell signaling	Gene Ontology	GO:0007267	11	0.001038
cell morphogenesis	Gene Ontology	GO:0000902	7	0.001174
synapse	Gene Ontology	GO:0045202	15	0.00122
perinuclear region of cytoplasm	Gene Ontology	GO:0048471	20	0.00123
regulation of cell shape	Gene Ontology	GO:0008360	9	0.00123
transport across blood-brain barrier	Gene Ontology	GO:0150104	7	0.001385
protein homotetramerization	Gene Ontology	GO:0051289	6	0.001787
potassium ion transmembrane transport	Gene Ontology	GO:0071805	8	0.001899
positive regulation of gene expression	Gene Ontology	GO:0010628	14	0.001994
epithelial cell maturation	Gene Ontology	GO:0002070	3	0.0021
frizzled binding	Gene Ontology	GO:0005109	5	0.002446
anatomical structure morphogenesis	Gene Ontology	GO:0009653	8	0.002635
positive regulation of GTPase activity	Gene Ontology	GO:0043547	12	0.002829
calcium ion binding	Gene Ontology	GO:0005509	19	0.002843
animal organ morphogenesis	Gene Ontology	GO:0009887	8	0.002902

#Term	Database	ID	Input number	Corrected P-Value
electron transport chain	Gene Ontology	GO:0022900	6	0.003222
nuclear receptor activity	Gene Ontology	GO:0004879	5	0.003222
branching involved in ureteric bud morphogenesis	Gene Ontology	GO:0001658	5	0.003222
cytoskeleton organization	Gene Ontology	GO:0007010	8	0.003229
tissue homeostasis	Gene Ontology	GO:0001894	4	0.003229
canonical Wnt signaling pathway	Gene Ontology	GO:0060070	6	0.003308
eye development	Gene Ontology	GO:0001654	5	0.003401
cell adhesion	Gene Ontology	GO:0007155	15	0.003461
negative regulation of transcription, DNA-templated	Gene Ontology	GO:0045892	16	0.003522
transcription regulator complex	Gene Ontology	GO:0005667	10	0.003566
integrin binding	Gene Ontology	GO:0005178	8	0.003604
muscle contraction	Gene Ontology	GO:0006936	7	0.003604
neuronal cell body	Gene Ontology	GO:0043025	13	0.003784
apoptotic process	Gene Ontology	GO:0006915	16	0.004136
metanephric mesenchyme development	Gene Ontology	GO:0072075	3	0.004438
cornification	Gene Ontology	GO:0070268	7	0.004703
membrane raft	Gene Ontology	GO:0045121	10	0.004745
extracellular region	Gene Ontology	GO:0005576	35	0.004783
negative regulation of cell population proliferation	Gene Ontology	GO:0008285	13	0.005378
branching involved in labyrinthine layer morphogenesis	Gene Ontology	GO:0060670	3	0.005409
negative regulation of cell migration	Gene Ontology	GO:0030336	7	0.005422
intracellular receptor signaling pathway	Gene Ontology	GO:0030522	4	0.00544
regulation of transcription by RNA polymerase II	Gene Ontology	GO:0006357	19	0.00557
cAMP-mediated signaling	Gene Ontology	GO:0019933	4	0.005972
actin filament organization	Gene Ontology	GO:0007015	7	0.005972
sequence-specific DNA binding	Gene Ontology	GO:0043565	13	0.006351
multicellular organism development	Gene Ontology	GO:0007275	14	0.006483
protein phosphatase binding	Gene Ontology	GO:0019903	6	0.007184
regulation of cell motility	Gene Ontology	GO:2000145	4	0.007237
regulation of Rho protein signal transduction	Gene Ontology	GO:0035023	4	0.007237
tongue development	Gene Ontology	GO:0043586	3	0.007362
carnitine shuttle	Gene Ontology	GO:0006853	3	0.007362
cellular response to osmotic stress	Gene Ontology	GO:0071470	3	0.007362
protein processing	Gene Ontology	GO:0016485	5	0.007513
structural molecule activity	Gene Ontology	GO:0005198	8	0.00788
cell differentiation	Gene Ontology	GO:0030154	16	0.007996
transcription regulatory region sequence-specific DNA binding	Gene Ontology	GO:0000976	10	0.008134
protein homodimerization activity	Gene Ontology	GO:0042803	17	0.008134
positive regulation of epithelial cell proliferation	Gene Ontology	GO:0050679	5	0.00888
SH3 domain binding	Gene Ontology	GO:0017124	7	0.009096
positive regulation of synapse assembly	Gene Ontology	GO:0051965	5	0.009976
positive regulation of chromatin binding	Gene Ontology	GO:0035563	3	0.009976

#Term	Database	ID	Input number	Corrected P-Value
sarcolemma	Gene Ontology	GO:0042383	6	0.010737
integrin-mediated signaling pathway	Gene Ontology	GO:0007229	6	0.010737
sodium ion transmembrane transport	Gene Ontology	GO:0035725	6	0.010737
chromatin binding	Gene Ontology	GO:0003682	13	0.011332
negative regulation of neuron apoptotic process	Gene Ontology	GO:0043524	7	0.011332
negative regulation of glycolytic process	Gene Ontology	GO:0045820	3	0.011356
neuron differentiation	Gene Ontology	GO:0030182	7	0.011472
cell-cell junction	Gene Ontology	GO:0005911	8	0.011472
neuron projection	Gene Ontology	GO:0043005	11	0.012084
Golgi lumen	Gene Ontology	GO:0005796	6	0.012221
circadian regulation of gene expression	Gene Ontology	GO:0032922	5	0.012427
cell cortex	Gene Ontology	GO:0005938	7	0.01247
negative chemotaxis	Gene Ontology	GO:0050919	4	0.012636
glycosaminoglycan biosynthetic process	Gene Ontology	GO:0006024	4	0.012636
cellular response to peptide	Gene Ontology	GO:1901653	3	0.012636
regulation of epithelial cell proliferation	Gene Ontology	GO:0050678	3	0.012636
cytoplasmic vesicle membrane	Gene Ontology	GO:0030659	7	0.01304
Wnt signaling pathway	Gene Ontology	GO:0016055	8	0.01304
protein C-terminus binding	Gene Ontology	GO:0008022	8	0.01304
positive regulation of canonical Wnt signaling pathway	Gene Ontology	GO:0090263	7	0.013324
epithelial cell differentiation	Gene Ontology	GO:0030855	5	0.013324
cellular response to estradiol stimulus	Gene Ontology	GO:0071392	4	0.013324
negative regulation of epithelial cell migration	Gene Ontology	GO:0010633	3	0.014137
embryonic digestive tract development	Gene Ontology	GO:0048566	3	0.014137
osteoblast differentiation	Gene Ontology	GO:0001649	6	0.014166
protein dephosphorylation	Gene Ontology	GO:0006470	7	0.014989
positive regulation of pri-miRNA transcription by RNA polymerase II	Gene Ontology	GO:1902895	4	0.015239
negative regulation of protein phosphorylation	Gene Ontology	GO:0001933	5	0.015239
positive regulation of synaptic transmission	Gene Ontology	GO:0050806	3	0.015961
electron transfer activity	Gene Ontology	GO:0009055	5	0.017566
defense response	Gene Ontology	GO:0006952	5	0.017566
camera-type eye morphogenesis	Gene Ontology	GO:0048593	3	0.017726
positive regulation of branching involved in ureteric bud morphogenesis	Gene Ontology	GO:0090190	3	0.017726
cadherin binding involved in cell-cell adhesion	Gene Ontology	GO:0098641	3	0.017726
cellular response to epidermal growth factor stimulus	Gene Ontology	GO:0071364	4	0.018487
cellular response to hypoxia	Gene Ontology	GO:0071456	6	0.018723
cell development	Gene Ontology	GO:0048468	3	0.019746
costamere	Gene Ontology	GO:0043034	3	0.019746
receptor complex	Gene Ontology	GO:0043235	8	0.019772
intermediate filament	Gene Ontology	GO:0005882	6	0.020611

#Term	Database	ID	Input number	Corrected P-Value
negative regulation of gene expression	Gene Ontology	GO:0010629	8	0.021403
negative regulation of fat cell differentiation	Gene Ontology	GO:0045599	4	0.02203
cerebellum development	Gene Ontology	GO:0021549	4	0.02203
bicellular tight junction	Gene Ontology	GO:0005923	6	0.024089
intermediate filament cytoskeleton organization	Gene Ontology	GO:0045104	3	0.024089
Rac guanyl-nucleotide exchange factor activity	Gene Ontology	GO:0030676	3	0.024089
regulation of AMPA receptor activity	Gene Ontology	GO:2000311	3	0.024089
basolateral plasma membrane	Gene Ontology	GO:0016323	8	0.024138
positive regulation of cell population proliferation	Gene Ontology	GO:0008284	13	0.024303
transmembrane receptor protein tyrosine kinase signaling pathway	Gene Ontology	GO:0007169	6	0.02447
lung development	Gene Ontology	GO:0030324	5	0.024867
establishment or maintenance of cell polarity	Gene Ontology	GO:0007163	4	0.025517
endomembrane system	Gene Ontology	GO:0012505	6	0.025573
negative regulation of cell growth	Gene Ontology	GO:0030308	6	0.025573
apical junction complex	Gene Ontology	GO:0043296	3	0.025573
actin polymerization or depolymerization	Gene Ontology	GO:0008154	3	0.025573
carbohydrate binding	Gene Ontology	GO:0030246	7	0.026426
axon guidance	Gene Ontology	GO:0007411	8	0.026581
cell projection	Gene Ontology	GO:0042995	6	0.026581
positive regulation of fatty acid oxidation	Gene Ontology	GO:0046321	2	0.026581
reproductive system development	Gene Ontology	GO:0061458	2	0.026581
mesenchyme migration	Gene Ontology	GO:0090131	2	0.026581
saliva secretion	Gene Ontology	GO:0046541	2	0.026581
anchored component of presynaptic membrane	Gene Ontology	GO:0099026	2	0.026581
regulation of nuclear cell cycle DNA replication	Gene Ontology	GO:0033262	2	0.026581
trigeminal ganglion development	Gene Ontology	GO:0061551	2	0.026581
protein phosphorylation	Gene Ontology	GO:0006468	12	0.026948
cell periphery	Gene Ontology	GO:0071944	4	0.027104
actin filament	Gene Ontology	GO:0005884	5	0.027458
response to drug	Gene Ontology	GO:0042493	9	0.028168
cytokine-mediated signaling pathway	Gene Ontology	GO:0019221	9	0.028995
positive regulation of angiogenesis	Gene Ontology	GO:0045766	6	0.028995
cochlea morphogenesis	Gene Ontology	GO:0090103	3	0.028995
glucose transmembrane transport	Gene Ontology	GO:1904659	3	0.028995
protein tyrosine kinase activity	Gene Ontology	GO:0004713	5	0.028995
protein kinase binding	Gene Ontology	GO:0019901	12	0.030178
Golgi apparatus	Gene Ontology	GO:0005794	20	0.0303
ion channel binding	Gene Ontology	GO:0044325	6	0.0303
symporter activity	Gene Ontology	GO:0015293	4	0.0303
cytoskeletal protein binding	Gene Ontology	GO:0008092	4	0.0303
positive regulation of cardiac muscle cell proliferation	Gene Ontology	GO:0060045	3	0.0303

#Term	Database	ID	Input number	Corrected P-Value
synaptic membrane adhesion	Gene Ontology	GO:0099560	3	0.0303
retinoic acid metabolic process	Gene Ontology	GO:0042573	3	0.0303
phospholipid translocation	Gene Ontology	GO:0045332	3	0.0303
extracellular matrix	Gene Ontology	GO:0031012	8	0.0303
lactate transmembrane transporter activity	Gene Ontology	GO:0015129	2	0.0303
site of polarized growth	Gene Ontology	GO:0030427	2	0.0303
positive regulation of potassium ion transmembrane transporter activity	Gene Ontology	GO:1901018	2	0.0303
embryonic skeletal joint development	Gene Ontology	GO:0072498	2	0.0303
peptide cross-linking via chondroitin 4-sulfate glycosaminoglycan	Gene Ontology	GO:0019800	2	0.0303
heart process	Gene Ontology	GO:0003015	2	0.0303
D-glucose transmembrane transporter activity	Gene Ontology	GO:0055056	2	0.0303
RNA polymerase II transcription coactivator binding	Gene Ontology	GO:0001225	2	0.0303
regulation of protein localization to membrane	Gene Ontology	GO:1905475	2	0.0303
negative regulation of ERBB signaling pathway	Gene Ontology	GO:1901185	2	0.0303
regulation of metanephric nephron tubule epithelial cell differentiation	Gene Ontology	GO:0072307	2	0.0303
L-glutamate import across plasma membrane	Gene Ontology	GO:0098712	2	0.0303
coreceptor activity involved in Wnt signaling pathway, planar cell polarity pathway	Gene Ontology	GO:1904929	2	0.0303
cell fate commitment	Gene Ontology	GO:0045165	4	0.030553
regulation of glucose metabolic process	Gene Ontology	GO:0010906	3	0.032076
cellular response to transforming growth factor beta stimulus	Gene Ontology	GO:0071560	4	0.032076
angiogenesis	Gene Ontology	GO:0001525	8	0.03296
inner ear morphogenesis	Gene Ontology	GO:0042472	4	0.033439
kidney development	Gene Ontology	GO:0001822	5	0.033439
embryonic pattern specification	Gene Ontology	GO:0009880	3	0.034477
focal adhesion assembly	Gene Ontology	GO:0048041	3	0.034477
ruffle membrane	Gene Ontology	GO:0032587	5	0.034477
heart development	Gene Ontology	GO:0007507	7	0.034707
positive regulation of smooth muscle cell proliferation	Gene Ontology	GO:0048661	4	0.034708
positive regulation of autophagy	Gene Ontology	GO:0010508	4	0.034708
protein serine/threonine kinase activity	Gene Ontology	GO:0004674	10	0.034762
cell-cell adhesion	Gene Ontology	GO:0098609	6	0.034762
bud elongation involved in lung branching	Gene Ontology	GO:0060449	2	0.034762
smoothened signaling pathway involved in dorsal/ventral neural tube patterning	Gene Ontology	GO:0060831	2	0.034762
lacrimal gland development	Gene Ontology	GO:0032808	2	0.034762
prostate gland epithelium morphogenesis	Gene Ontology	GO:0060740	2	0.034762
generation of neurons	Gene Ontology	GO:0048699	2	0.034762
mesenchymal cell differentiation	Gene Ontology	GO:0048762	2	0.034762

#Term	Database	ID	Input number	Corrected P-Value
modification-dependent protein catabolic process	Gene Ontology	GO:0019941	2	0.034762
thyroid hormone binding	Gene Ontology	GO:0070324	2	0.034762
negative regulation of neuron differentiation	Gene Ontology	GO:0045665	4	0.034762
cellular response to growth factor stimulus	Gene Ontology	GO:0071363	4	0.034762
ruffle	Gene Ontology	GO:0001726	5	0.034762
outflow tract septum morphogenesis	Gene Ontology	GO:0003148	3	0.034762
hair follicle morphogenesis	Gene Ontology	GO:0031069	3	0.034762
chemorepellent activity	Gene Ontology	GO:0045499	3	0.034762
viral life cycle	Gene Ontology	GO:0019058	3	0.034762
positive regulation of osteoblast differentiation	Gene Ontology	GO:0045669	4	0.036084
sensory perception of sound	Gene Ontology	GO:0007605	6	0.036084
ion transport	Gene Ontology	GO:0006811	5	0.037284
potassium channel activity	Gene Ontology	GO:0005267	3	0.037342
myelin sheath	Gene Ontology	GO:0043209	3	0.037342
positive regulation of DNA replication	Gene Ontology	GO:0045740	3	0.037342
regulation of ERK1 and ERK2 cascade	Gene Ontology	GO:0070372	3	0.037342
positive regulation of MAP kinase activity	Gene Ontology	GO:0043406	4	0.037507
structural constituent of cytoskeleton	Gene Ontology	GO:0005200	5	0.038073
nuclear body	Gene Ontology	GO:0016604	9	0.038636
extracellular matrix organization	Gene Ontology	GO:0030198	8	0.039029
brown fat cell differentiation	Gene Ontology	GO:0050873	3	0.039029
pattern specification process	Gene Ontology	GO:0007389	3	0.039029
intracellular signal transduction	Gene Ontology	GO:0035556	10	0.039029
response to type I interferon	Gene Ontology	GO:0034340	2	0.039029
regulation of cardiac muscle contraction by calcium ion signaling	Gene Ontology	GO:0010882	2	0.039029
retinal dehydrogenase activity	Gene Ontology	GO:0001758	2	0.039029
drug transmembrane transport	Gene Ontology	GO:0006855	2	0.039029
regulation of keratinocyte proliferation	Gene Ontology	GO:0010837	2	0.039029
artery development	Gene Ontology	GO:0060840	2	0.039029
regulation of fatty acid metabolic process	Gene Ontology	GO:0019217	2	0.039029
cell morphogenesis involved in neuron differentiation	Gene Ontology	GO:0048667	2	0.039029
positive regulation of collateral sprouting	Gene Ontology	GO:0048672	2	0.039029
axon	Gene Ontology	GO:0030424	9	0.039029
synapse assembly	Gene Ontology	GO:0007416	4	0.039102
calmodulin binding	Gene Ontology	GO:0005516	7	0.039505
hippocampus development	Gene Ontology	GO:0021766	4	0.04035
receptor signaling pathway via JAK-STAT	Gene Ontology	GO:0007259	3	0.04035
cellular response to dexamethasone stimulus	Gene Ontology	GO:0071549	3	0.04035
ERBB2 signaling pathway	Gene Ontology	GO:0038128	3	0.04035
embryonic organ development	Gene Ontology	GO:0048568	3	0.04035
in utero embryonic development	Gene Ontology	GO:0001701	7	0.040565
lateral plasma membrane	Gene Ontology	GO:0016328	4	0.042007

#Term	Database	ID	Input number	Corrected P-Value
blood vessel remodeling	Gene Ontology	GO:0001974	3	0.042735
embryonic cranial skeleton morphogenesis	Gene Ontology	GO:0048701	3	0.042735
regulation of angiogenesis	Gene Ontology	GO:0045765	3	0.042735
MAPK cascade	Gene Ontology	GO:0000165	8	0.043132
myoblast migration	Gene Ontology	GO:0051451	2	0.043132
regulation of neurotransmitter receptor localization to postsynaptic specialization membrane	Gene Ontology	GO:0098696	2	0.043132
negative regulation of organ growth	Gene Ontology	GO:0046621	2	0.043132
cellular response to testosterone stimulus	Gene Ontology	GO:0071394	2	0.043132
stem cell proliferation	Gene Ontology	GO:0072089	2	0.043132
cellular response to retinoic acid	Gene Ontology	GO:0071300	4	0.043976
positive regulation of axonogenesis	Gene Ontology	GO:0050772	3	0.044124
odontogenesis	Gene Ontology	GO:0042476	3	0.044124
positive regulation of MAPK cascade	Gene Ontology	GO:0043410	5	0.044124
transcription factor binding	Gene Ontology	GO:0008134	9	0.046773
dendrite morphogenesis	Gene Ontology	GO:0048813	3	0.046773
positive regulation of vascular associated smooth muscle cell proliferation	Gene Ontology	GO:1904707	3	0.046773
positive regulation of cell differentiation	Gene Ontology	GO:0045597	3	0.046773
semaphorin-plexin signaling pathway	Gene Ontology	GO:0071526	3	0.046773
positive regulation of axon extension	Gene Ontology	GO:0045773	3	0.046773
stress fiber	Gene Ontology	GO:0001725	4	0.046855
RNA polymerase II transcription regulatory region sequence-specific DNA binding	Gene Ontology	GO:0000977	9	0.047571
RNA splicing	Gene Ontology	GO:0008380	6	0.047617
microvillus	Gene Ontology	GO:0005902	4	0.048195
fat cell differentiation	Gene Ontology	GO:0045444	4	0.048195
transepithelial transport	Gene Ontology	GO:0070633	2	0.048195
cellular response to caffeine	Gene Ontology	GO:0071313	2	0.048195
glutamate binding	Gene Ontology	GO:0016595	2	0.048195
opioid receptor signaling pathway	Gene Ontology	GO:0038003	2	0.048195
cation channel complex	Gene Ontology	GO:0034703	2	0.048195
negative regulation of cell growth involved in cardiac muscle cell development	Gene Ontology	GO:0061052	2	0.048195
energy homeostasis	Gene Ontology	GO:0097009	3	0.048621
negative regulation of extrinsic apoptotic signaling pathway	Gene Ontology	GO:2001237	3	0.048621
homophilic cell adhesion via plasma membrane adhesion molecules	Gene Ontology	GO:0007156	6	0.048958
Wnt signaling pathway	KEGG PATHWAY	hsa04310	11	9.1E-05
Hippo signaling pathway - multiple species	KEGG PATHWAY	hsa04392	6	9.1E-05
Insulin resistance	KEGG PATHWAY	hsa04931	9	0.000163
Glucagon signaling pathway	KEGG PATHWAY	hsa04922	8	0.000851

#Term	Database	ID	Input number	Corrected P-Value
Proteoglycans in cancer	KEGG PATHWAY	hsa05205	10	0.002008
Adipocytokine signaling pathway	KEGG PATHWAY	hsa04920	6	0.003222
@@Axon guidance	KEGG PATHWAY	hsa04360	9	0.003461
Gastric cancer	KEGG PATHWAY	hsa05226	8	0.004659
Hippo signaling pathway	KEGG PATHWAY	hsa04390	8	0.005414
Insulin secretion	KEGG PATHWAY	hsa04911	6	0.007184
Rap1 signaling pathway	KEGG PATHWAY	hsa04015	9	0.007362
cAMP signaling pathway	KEGG PATHWAY	hsa04024	9	0.008041
Salivary secretion	KEGG PATHWAY	hsa04970	6	0.008118
Vascular smooth muscle contraction	KEGG PATHWAY	hsa04270	7	0.009385
Insulin signaling pathway	KEGG PATHWAY	hsa04910	7	0.010999
Renin secretion	KEGG PATHWAY	hsa04924	5	0.014039
Pathways in cancer	KEGG PATHWAY	hsa05200	14	0.015961
Focal adhesion	KEGG PATHWAY	hsa04510	8	0.016612
EGFR tyrosine kinase inhibitor resistance	KEGG PATHWAY	hsa01521	5	0.021628
Regulation of actin cytoskeleton	KEGG PATHWAY	hsa04810	8	0.023497
cGMP-PKG signaling pathway	KEGG PATHWAY	hsa04022	7	0.024089
Metabolic pathways	KEGG PATHWAY	hsa01100	26	0.028037
Protein digestion and absorption	KEGG PATHWAY	hsa04974	5	0.030178
Estrogen signaling pathway	KEGG PATHWAY	hsa04915	6	0.03296
Signaling pathways regulating pluripotency of stem cells	KEGG PATHWAY	hsa04550	6	0.034477
Prostate cancer	KEGG PATHWAY	hsa05215	5	0.034762
Pancreatic secretion	KEGG PATHWAY	hsa04972	5	0.034762
Aldosterone synthesis and secretion	KEGG PATHWAY	hsa04925	5	0.034762
Melanogenesis	KEGG PATHWAY	hsa04916	5	0.038073
Breast cancer	KEGG PATHWAY	hsa05224	6	0.038663
Adrenergic signaling in cardiomyocytes	KEGG PATHWAY	hsa04261	6	0.039102
Basal cell carcinoma	KEGG PATHWAY	hsa05217	4	0.039102
Parathyroid hormone synthesis, secretion and action	KEGG PATHWAY	hsa04928	5	0.042007
Oxytocin signaling pathway	KEGG PATHWAY	hsa04921	6	0.042294
Hepatitis C	KEGG PATHWAY	hsa05160	6	0.043132
Human papillomavirus infection	KEGG PATHWAY	hsa05165	9	0.048865
Glutamatergic synapse	KEGG PATHWAY	hsa04724	5	0.049496
Signal Transduction	Reactome	R-HSA-162582	68	4.02E-10
Rho GTPase cycle	Reactome	R-HSA-194840	12	5.32E-06
Metabolism	Reactome	R-HSA-1430728	47	1.63E-05
Developmental Biology	Reactome	R-HSA-1266738	30	5.9E-05
Transcriptional regulation by RUNX2	Reactome	R-HSA-8878166	10	5.92E-05
Signaling by Rho GTPases	Reactome	R-HSA-194315	17	0.00025
Transport of small molecules	Reactome	R-HSA-382551	22	0.000262
RUNX3 regulates YAP1-mediated transcription	Reactome	R-HSA-8951671	4	0.000262
Axon guidance	Reactome	R-HSA-422475	18	0.000697

#Term	Database	ID	Input number	Corrected P-Value
Netrin-1 signaling	Reactome	R-HSA-373752	6	0.000834
Disease	Reactome	R-HSA-1643685	26	0.001024
Signaling by PTK6	Reactome	R-HSA-8848021	6	0.001042
Signaling by Non-Receptor Tyrosine Kinases	Reactome	R-HSA-9006927	6	0.001042
YAP1- and WWTR1 (TAZ)-stimulated gene expression	Reactome	R-HSA-2032785	4	0.001174
Generic Transcription Pathway	Reactome	R-HSA-212436	27	0.002538
Signaling by Receptor Tyrosine Kinases	Reactome	R-HSA-9006934	15	0.002635
p75 NTR receptor-mediated signalling	Reactome	R-HSA-193704	7	0.002802
Smooth Muscle Contraction	Reactome	R-HSA-445355	5	0.002843
Defective B4GALT7 causes EDS, progeroid type	Reactome	R-HSA-3560783	4	0.002928
Defective B3GAT3 causes JDSSDHD	Reactome	R-HSA-3560801	4	0.002928
Defective B3GALT6 causes EDSP2 and SEMDJL1	Reactome	R-HSA-4420332	4	0.002928
Regulation of FZD by ubiquitination	Reactome	R-HSA-4641263	4	0.003229
Cell death signalling via NRAGE, NRIF and NADE	Reactome	R-HSA-204998	6	0.004546
SUMOylation of intracellular receptors	Reactome	R-HSA-4090294	4	0.004546
Metabolism of carbohydrates	Reactome	R-HSA-71387	11	0.005075
A tetrasaccharide linker sequence is required for GAG synthesis	Reactome	R-HSA-1971475	4	0.00544
Downregulation of ERBB2 signaling	Reactome	R-HSA-8863795	4	0.005972
SLC-mediated transmembrane transport	Reactome	R-HSA-425407	10	0.006187
RNA Polymerase II Transcription	Reactome	R-HSA-73857	27	0.007237
Muscle contraction	Reactome	R-HSA-397014	9	0.007237
Signaling by ERBB4	Reactome	R-HSA-1236394	5	0.007237
Heparan sulfate/heparin (HS-GAG) metabolism	Reactome	R-HSA-1638091	5	0.007237
ERBB2 Activates PTK6 Signaling	Reactome	R-HSA-8847993	3	0.007362
RUNX2 regulates bone development	Reactome	R-HSA-8941326	4	0.008182
Downregulation of ERBB2:ERBB3 signaling	Reactome	R-HSA-1358803	3	0.008623
Immune System	Reactome	R-HSA-168256	37	0.009293
PI3K events in ERBB2 signaling	Reactome	R-HSA-1963642	3	0.009976
DCC mediated attractive signaling	Reactome	R-HSA-418885	3	0.009976
ERBB2 Regulates Cell Motility	Reactome	R-HSA-6785631	3	0.009976
Semaphorin interactions	Reactome	R-HSA-373755	5	0.011356
Import of palmitoyl-CoA into the mitochondrial matrix	Reactome	R-HSA-200425	3	0.011356
GRB2 events in ERBB2 signaling	Reactome	R-HSA-1963640	3	0.011356
Death Receptor Signalling	Reactome	R-HSA-73887	7	0.011823
Ion channel transport	Reactome	R-HSA-983712	8	0.012084
Diseases of glycosylation	Reactome	R-HSA-3781865	7	0.012636
Nuclear Receptor transcription pathway	Reactome	R-HSA-383280	4	0.015239
Diseases associated with glycosaminoglycan metabolism	Reactome	R-HSA-3560782	4	0.016349
Metabolism of lipids	Reactome	R-HSA-556833	17	0.01751
Gene expression (Transcription)	Reactome	R-HSA-74160	27	0.019427
SHC1 events in ERBB2 signaling	Reactome	R-HSA-1250196	3	0.019746
Signaling by Hippo	Reactome	R-HSA-2028269	3	0.019746

#Term	Database	ID	Input number	Corrected P-Value
Sema4D induced cell migration and growth-cone collapse	Reactome	R-HSA-416572	3	0.02203
Signaling by ERBB2	Reactome	R-HSA-1227986	4	0.023523
Signaling by NOTCH3	Reactome	R-HSA-9012852	4	0.024457
Glycosaminoglycan metabolism	Reactome	R-HSA-1630316	6	0.02447
Post-translational protein modification	Reactome	R-HSA-597592	26	0.025435
SUMO E3 ligases SUMOylate target proteins	Reactome	R-HSA-3108232	7	0.025517
Chondroitin sulfate/dermatan sulfate metabolism	Reactome	R-HSA-1793185	4	0.025517
NOTCH2 Activation and Transmission of Signal to the Nucleus	Reactome	R-HSA-2979096	3	0.025573
Signaling by WNT	Reactome	R-HSA-195721	10	0.025994
RUNX2 regulates osteoblast differentiation	Reactome	R-HSA-8940973	3	0.027104
Formation of the cornified envelope	Reactome	R-HSA-6809371	6	0.02774
SUMOylation	Reactome	R-HSA-2990846	7	0.028079
Sema4D in semaphorin signaling	Reactome	R-HSA-400685	3	0.028995
Macroautophagy	Reactome	R-HSA-1632852	5	0.028995
TCF dependent signaling in response to WNT	Reactome	R-HSA-201681	8	0.0303
EPH-Ephrin signaling	Reactome	R-HSA-2682334	5	0.0303
NOTCH3 Activation and Transmission of Signal to the Nucleus	Reactome	R-HSA-9013507	3	0.0303
Transcriptional regulation by RUNX3	Reactome	R-HSA-8878159	5	0.031523
Metabolism of proteins	Reactome	R-HSA-392499	33	0.03206
Fructose metabolism	Reactome	R-HSA-5652084	2	0.034762
NRAGE signals death through JNK	Reactome	R-HSA-193648	4	0.034762
Constitutive Signaling by Aberrant PI3K in Cancer	Reactome	R-HSA-2219530	4	0.039029
Defective CHST14 causes EDS, musculocontractural type	Reactome	R-HSA-3595174	2	0.039029
Defective CHST3 causes SEDCJD	Reactome	R-HSA-3595172	2	0.039029
Defective CHSY1 causes TPBS	Reactome	R-HSA-3595177	2	0.039029
HS-GAG biosynthesis	Reactome	R-HSA-2022928	3	0.04035
Activated NOTCH1 Transmits Signal to the Nucleus	Reactome	R-HSA-2122948	3	0.04035
Nuclear signaling by ERBB4	Reactome	R-HSA-1251985	3	0.04035
Diseases of signal transduction	Reactome	R-HSA-5663202	10	0.04077
Termination of translesion DNA synthesis	Reactome	R-HSA-5656169	3	0.042735
Transport of inorganic cations/anions and amino acids/oligopeptides	Reactome	R-HSA-425393	5	0.042876
Stimuli-sensing channels	Reactome	R-HSA-2672351	5	0.043132
Autophagy	Reactome	R-HSA-9612973	5	0.043132
Ca2+ activated K+ channels	Reactome	R-HSA-1296052	2	0.043132
Myoclonic epilepsy of Lafora	Reactome	R-HSA-3785653	2	0.043132
Interferon alpha/beta signaling	Reactome	R-HSA-909733	4	0.043976
Signaling by NOTCH2	Reactome	R-HSA-1980145	3	0.044124
O-linked glycosylation	Reactome	R-HSA-5173105	5	0.046773
Negative regulators of DDX58/IFIH1 signaling	Reactome	R-HSA-936440	3	0.046773
ISG15 antiviral mechanism	Reactome	R-HSA-1169408	4	0.046855
Circadian Clock	Reactome	R-HSA-400253	4	0.046855

#Term	Database	ID	Input number	Corrected P-Value
Activation of PPARGC1A (PGC-1alpha) by phosphorylation	Reactome	R-HSA-2151209	2	0.048195
PPARA activates gene expression	Reactome	R-HSA-1989781	5	0.049496
