# Supplementary material for: Transcriptome analysis reveals gene expression changes of pigs infected with non-lethal African swine fever virus
Source: Genet Mol Biol. 2023 Oct 13;46(3):e20230037. doi: 10.1590/1678-4685-GMB-2023-0037 (PMC10578457; doi:10.1590/1678-4685-GMB-2023-0037)
Supplement: Table S8 - [file 1415-4757-GMB-46-3-e20230037-s10.pdf]

Genetics and Molecular Biology

https://doi.org/10.1590/1678-4685-GMB-2023-0037

# Supplementary Material to "Transcriptome analysis reveals gene expression changes of pigs infected with non-lethal African swine fever virus"

**Table S8** - The enriched pathways of DEGs in spleen using KOBAS 3.0.

| #Term | Database | ID | Input number | Corrected P-Value |
|---|---|---|---|---|
| extracellular exosome | Gene Ontology | GO:0070062 | 28 | 3.9E-10 |
| protein binding | Gene Ontology | GO:0005515 | 64 | 1.9E-09 |
| extracellular space | Gene Ontology | GO:0005615 | 21 | 1.46E-07 |
| mitochondrion | Gene Ontology | GO:0005739 | 19 | 1.46E-07 |
| extracellular region | Gene Ontology | GO:0005576 | 20 | 8.59E-06 |
| cytosol | Gene Ontology | GO:0005829 | 34 | 1.15E-05 |
| cytoplasm | Gene Ontology | GO:0005737 | 31 | 4.03E-05 |
| neutrophil degranulation | Gene Ontology | GO:0043312 | 10 | 6.1E-05 |
| plasma membrane | Gene Ontology | GO:0005886 | 29 | 0.000256 |
| response to ethanol | Gene Ontology | GO:0045471 | 5 | 0.000561 |
| chaperone binding | Gene Ontology | GO:0051087 | 5 | 0.000701 |
| mitochondrial proton-transporting ATP synthase complex | Gene Ontology | GO:0005753 | 3 | 0.001099 |
| proton-transporting ATP synthase activity, rotational mechanism | Gene Ontology | GO:0046933 | 3 | 0.001229 |
| mitochondrial ATP synthesis coupled proton transport | Gene Ontology | GO:0042776 | 3 | 0.001447 |
| mitochondrial matrix | Gene Ontology | GO:0005759 | 7 | 0.002805 |
| collagen-containing extracellular matrix | Gene Ontology | GO:0062023 | 7 | 0.002805 |
| cellular oxidant detoxification | Gene Ontology | GO:0098869 | 4 | 0.002805 |
| ATP biosynthetic process | Gene Ontology | GO:0006754 | 3 | 0.002805 |
| cristae formation | Gene Ontology | GO:0042407 | 3 | 0.003818 |
| structural molecule activity | Gene Ontology | GO:0005198 | 5 | 0.003818 |
| response to drug | Gene Ontology | GO:0042493 | 6 | 0.003944 |
| integral component of membrane | Gene Ontology | GO:0016021 | 22 | 0.004517 |
| proton transmembrane transport | Gene Ontology | GO:1902600 | 4 | 0.007497 |
| secretory granule lumen | Gene Ontology | GO:0034774 | 4 | 0.008633 |
| membrane | Gene Ontology | GO:0016020 | 15 | 0.008907 |
| interleukin-12-mediated signaling pathway | Gene Ontology | GO:0035722 | 3 | 0.009124 |
| peripheral nervous system myelin maintenance | Gene Ontology | GO:0032287 | 2 | 0.009124 |
| ficolin-1-rich granule lumen | Gene Ontology | GO:1904813 | 4 | 0.009966 |
| positive regulation of cholesterol esterification | Gene Ontology | GO:0010873 | 2 | 0.010161 |

| #Term | Database | ID | Input number | Corrected P-Value |
|---|---|---|---|---|
| mitochondrial proton-transporting ATP synthase complex, coupling factor F(o) | Gene Ontology | GO:0000276 | 2 | 0.010161 |
| sarcoplasm | Gene Ontology | GO:0016528 | 2 | 0.010161 |
| identical protein binding | Gene Ontology | GO:0042802 | 12 | 0.010169 |
| response to dexamethasone | Gene Ontology | GO:0071548 | 2 | 0.011267 |
| cytoskeletal protein binding | Gene Ontology | GO:0008092 | 3 | 0.011267 |
| oxidation-reduction process | Gene Ontology | GO:0055114 | 7 | 0.011267 |
| calcium ion binding | Gene Ontology | GO:0005509 | 8 | 0.011267 |
| tertiary granule lumen | Gene Ontology | GO:1904724 | 3 | 0.011267 |
| copper ion binding | Gene Ontology | GO:0005507 | 3 | 0.011267 |
| neuronal cell body | Gene Ontology | GO:0043025 | 6 | 0.011267 |
| negative regulation of ryanodine-sensitive calcium-release channel activity | Gene Ontology | GO:0060315 | 2 | 0.01394 |
| ATP synthesis coupled proton transport | Gene Ontology | GO:0015986 | 2 | 0.015362 |
| actin binding | Gene Ontology | GO:0003779 | 5 | 0.015362 |
| dendrite | Gene Ontology | GO:0030425 | 6 | 0.018035 |
| glycerophospholipid catabolic process | Gene Ontology | GO:0046475 | 2 | 0.019135 |
| growth factor activity | Gene Ontology | GO:0008083 | 4 | 0.019427 |
| NAD metabolic process | Gene Ontology | GO:0019674 | 2 | 0.020543 |
| positive regulation of cellular protein metabolic process | Gene Ontology | GO:0032270 | 2 | 0.020543 |
| phosphatidylinositol-3-phosphatase activity | Gene Ontology | GO:0004438 | 2 | 0.022267 |
| regulation of release of sequestered calcium ion into cytosol by sarcoplasmic reticulum | Gene Ontology | GO:0010880 | 2 | 0.024014 |
| positive regulation of macrophage derived foam cell differentiation | Gene Ontology | GO:0010744 | 2 | 0.024014 |
| costamere | Gene Ontology | GO:0043034 | 2 | 0.025903 |
| positive regulation of inflammatory response | Gene Ontology | GO:0050729 | 3 | 0.025903 |
| neurotransmitter receptor activity | Gene Ontology | GO:0030594 | 3 | 0.026096 |
| response to toxic substance | Gene Ontology | GO:0009636 | 3 | 0.026096 |
| regulation of cardiac muscle contraction by regulation of the release of sequestered calcium ion | Gene Ontology | GO:0010881 | 2 | 0.026842 |
| cholesterol homeostasis | Gene Ontology | GO:0042632 | 3 | 0.026842 |
| protein C-terminus binding | Gene Ontology | GO:0008022 | 4 | 0.027602 |
| insulin receptor signaling pathway | Gene Ontology | GO:0008286 | 3 | 0.027602 |
| calcium-independent cell-cell adhesion via plasma membrane cell-adhesion molecules | Gene Ontology | GO:0016338 | 2 | 0.027602 |
| muscle cell cellular homeostasis | Gene Ontology | GO:0046716 | 2 | 0.027602 |
| azurophil granule lumen | Gene Ontology | GO:0035578 | 3 | 0.029857 |
| positive regulation of apoptotic process | Gene Ontology | GO:0043065 | 5 | 0.030085 |
| xenobiotic metabolic process | Gene Ontology | GO:0006805 | 3 | 0.030085 |
| oxidoreductase activity, acting on the CH-OH group of donors, NAD or NADP as acceptor | Gene Ontology | GO:0016616 | 2 | 0.03218 |
| cellular response to fatty acid | Gene Ontology | GO:0071398 | 2 | 0.03218 |

| #Term | Database | ID | Input number | Corrected P-Value |
|---|---|---|---|---|
| cellular response to oxidative stress | Gene Ontology | GO:0034599 | 3 | 0.032414 |
| transforming growth factor beta receptor signaling pathway | Gene Ontology | GO:0007179 | 3 | 0.033007 |
| protein-containing complex binding | Gene Ontology | GO:0044877 | 5 | 0.033236 |
| cytochrome-c oxidase activity | Gene Ontology | GO:0004129 | 2 | 0.033236 |
| ion transmembrane transport | Gene Ontology | GO:0034220 | 4 | 0.033331 |
| ruffle | Gene Ontology | GO:0001726 | 3 | 0.033501 |
| signaling receptor binding | Gene Ontology | GO:0005102 | 5 | 0.03361 |
| triglyceride catabolic process | Gene Ontology | GO:0019433 | 2 | 0.03361 |
| regulation of glucose metabolic process | Gene Ontology | GO:0010906 | 2 | 0.03361 |
| response to oxidative stress | Gene Ontology | GO:0006979 | 3 | 0.03434 |
| basolateral plasma membrane | Gene Ontology | GO:0016323 | 4 | 0.03434 |
| fibronectin binding | Gene Ontology | GO:0001968 | 2 | 0.03434 |
| hydrogen peroxide catabolic process | Gene Ontology | GO:0042744 | 2 | 0.03434 |
| response to organic substance | Gene Ontology | GO:0010033 | 2 | 0.03434 |
| triglyceride homeostasis | Gene Ontology | GO:0070328 | 2 | 0.035397 |
| phosphatidylcholine acyl-chain remodeling | Gene Ontology | GO:0036151 | 2 | 0.035397 |
| regulation of mitochondrial membrane potential | Gene Ontology | GO:0051881 | 2 | 0.035397 |
| osteoblast differentiation | Gene Ontology | GO:0001649 | 3 | 0.036018 |
| phosphatidylinositol dephosphorylation | Gene Ontology | GO:0046856 | 2 | 0.036423 |
| integral component of plasma membrane | Gene Ontology | GO:0005887 | 10 | 0.036423 |
| reactive oxygen species metabolic process | Gene Ontology | GO:0072593 | 2 | 0.0375 |
| brown fat cell differentiation | Gene Ontology | GO:0050873 | 2 | 0.0375 |
| cornification | Gene Ontology | GO:0070268 | 3 | 0.038476 |
| mitochondrial inner membrane | Gene Ontology | GO:0005743 | 5 | 0.039417 |
| microtubule associated complex | Gene Ontology | GO:0005875 | 2 | 0.040888 |
| negative regulation of cell migration | Gene Ontology | GO:0030336 | 3 | 0.041018 |
| intermediate filament | Gene Ontology | GO:0005882 | 3 | 0.041977 |
| positive regulation of vascular associated smooth muscle cell proliferation | Gene Ontology | GO:1904707 | 2 | 0.04362 |
| tricarboxylic acid cycle | Gene Ontology | GO:0006099 | 2 | 0.04362 |
| bicellular tight junction | Gene Ontology | GO:0005923 | 3 | 0.045805 |
| cytoskeleton | Gene Ontology | GO:0005856 | 5 | 0.045805 |
| Metabolic pathways | KEGG PATHWAY | hsa01100 | 19 | 9.98E-07 |
| Oxidative phosphorylation | KEGG PATHWAY | hsa00190 | 7 | 1.23E-05 |
| Huntington disease | KEGG PATHWAY | hsa05016 | 7 | 8.87E-05 |
| Parkinson disease | KEGG PATHWAY | hsa05012 | 6 | 0.000223 |
| Thermogenesis | KEGG PATHWAY | hsa04714 | 6 | 0.001954 |
| Alzheimer disease | KEGG PATHWAY | hsa05010 | 5 | 0.003896 |
| Non-alcoholic fatty liver disease (NAFLD) | KEGG PATHWAY | hsa04932 | 4 | 0.015362 |
| Calcium signaling pathway | KEGG PATHWAY | hsa04020 | 4 | 0.028681 |
| Renin-angiotensin system | KEGG PATHWAY | hsa04614 | 2 | 0.030422 |
| Citrate cycle (TCA cycle) | KEGG PATHWAY | hsa00020 | 2 | 0.0375 |

| #Term | Database | ID | Input number | Corrected P-Value |
|---|---|---|---|---|
| Leukocyte transendothelial migration | KEGG PATHWAY | hsa04670 | 3 | 0.039106 |
| Carbon metabolism | KEGG PATHWAY | hsa01200 | 3 | 0.041977 |
| Metabolism | Reactome | R-HSA-1430728 | 26 | 5.17E-09 |
| The citric acid (TCA) cycle and respiratory electron transport | Reactome | R-HSA-1428517 | 7 | 5.56E-05 |
| Neutrophil degranulation | Reactome | R-HSA-6798695 | 10 | 6.1E-05 |
| Metabolism of lipids | Reactome | R-HSA-556833 | 11 | 0.000256 |
| Respiratory electron transport, ATP synthesis by chemiosmotic coupling, and heat production by uncoupling proteins. | Reactome | R-HSA-163200 | 5 | 0.001306 |
| Formation of ATP by chemiosmotic coupling | Reactome | R-HSA-163210 | 3 | 0.001306 |
| Innate Immune System | Reactome | R-HSA-168249 | 11 | 0.003816 |
| Cristae formation | Reactome | R-HSA-8949613 | 3 | 0.003896 |
| Mitochondrial biogenesis | Reactome | R-HSA-1592230 | 4 | 0.004681 |
| Detoxification of Reactive Oxygen Species | Reactome | R-HSA-3299685 | 3 | 0.00486 |
| Gene and protein expression by JAK-STAT signaling after Interleukin-12 stimulation | Reactome | R-HSA-8950505 | 3 | 0.005951 |
| Immune System | Reactome | R-HSA-168256 | 15 | 0.009124 |
| Interleukin-12 signaling | Reactome | R-HSA-9020591 | 3 | 0.009124 |
| Interleukin-12 family signaling | Reactome | R-HSA-447115 | 3 | 0.012194 |
| Organelle biogenesis and maintenance | Reactome | R-HSA-1852241 | 5 | 0.021764 |
| PI Metabolism | Reactome | R-HSA-1483255 | 3 | 0.026842 |
| Cell junction organization | Reactome | R-HSA-446728 | 3 | 0.029857 |
| Phospholipid metabolism | Reactome | R-HSA-1483257 | 4 | 0.03361 |
| Biological oxidations | Reactome | R-HSA-211859 | 4 | 0.035397 |
| Phase I - Functionalization of compounds | Reactome | R-HSA-211945 | 3 | 0.035407 |
| Tight junction interactions | Reactome | R-HSA-420029 | 2 | 0.036423 |
| Gluconeogenesis | Reactome | R-HSA-70263 | 2 | 0.041977 |
| Cell-Cell communication | Reactome | R-HSA-1500931 | 3 | 0.048365 |
