# Supplementary material for: Transcriptome analysis reveals gene expression changes of pigs infected with non-lethal African swine fever virus
Source: Genet Mol Biol. 2023 Oct 13;46(3):e20230037. doi: 10.1590/1678-4685-GMB-2023-0037 (PMC10578457; doi:10.1590/1678-4685-GMB-2023-0037)
Supplement: Table S9 - [file 1415-4757-GMB-46-3-e20230037-s11.pdf]

Genetics and Molecular Biology

https://doi.org/10.1590/1678-4685-GMB-2023-0037

Supplementary Material to "Transcriptome analysis reveals gene expression changes of pigs infected with non-lethal African swine fever virus"

Table S9 - Overlapping genes with our study.

Overlapping (Jaing et al., 2017)		Overlapping (Yang et al., 2021)			Overlapping (Hualin Sun., 2021)		
**GRG**	**OURT**	**12h**	**24h**	**36h**	**Healthy vs. acutely infected, dead pigs**	**Healthy vs. cohabiting asymptomatic pigs**	**Cohabiting asymptomatic vs. acutely infected, dead pigs**
S100A8	S100A8	CCDC114	ACHE	ACE	FGFRL1	AMPH	CD19
APOA4	ANGPTL4	CHST10	AHRR	AHNAK2	FXYD7	ARNT2	CD22
CXCL10	CXCL10	COL6A2	ARC	AHRR	HSPA2	AZGP1	CD79A
SERPINA3-2	NOS2	GANC	ATP8B2	APH1B	IGFBP1	BAG2	CD79B
UBA52	NCF2	IFIT1	BACH2	ARC	ROGDI	BLNK	CNFN
NOS2	SNRPD2	LOC100156553	BCAS4	ARHGAP6	SVIP	BTK	CR1
IDO1	ACSL1	LOC110258215	CD209	ATF3	TMPRSS2	C2H19orf24	CXCR2
SLPI	PHF23	PLIN1	CEMIP	BBS1		C9H1orf116	FRMPD1
MIR122	TRIM10	SCNN1A	CTTNBP2NL	BID		CD14	GSG1L
IFIT1	CMKLR1	TFAP2C	CXCL10	CCL2		CD79A	GSG1L
ROGDI	NFE2	ZMYND15	CYP26A1	CEMIP		EIF2S1	HSH2D
UBB	PLIN3	ZNF704	EPCAM	CLCA1		ESPN	LMAN1
ACSL1	NLRC5		EPHB3	CLIC4		ESPN	MMP3
AATK	FLOT1		FCN1	CRHBP		FGFR3	NUPR1

**Overlapping (Jaing et al., 2017)**		**Overlapping (Yang et al., 2021)**		**Overlapping (Hualin Sun., 2021)**	
SOX6	GPR84	FGD4	CXCL10	FGFRL1	PAX5
CD5L	ISG15	GCNT4	DDIT3	FOS	PPARG
CRAT	LOC595122	HOPX	DDX58	GADD45G	
SLC4A1	SOCS3	HR	DUSP10	GOLIM4	
SYT3	RBM38	IDO1	EPCAM	HSH2D	
GCNT4	VARS	IFIT1	EPOP	HSPA14	
TNF	DDIT4	IFITM1	F3	HSPA2	
FES	DDX58	ISG15	GPR180	IGF1	
CDC20	MYL9	ITGA7	HR	IGFBP1	
CCL2	ATP1B1	KLHL3	IFIT1	IGFBP2	
PLIN3	COX7A2	KRT8	IFITM1	JUN	
FLOT1	FCN1	LIF	IFNA1	LMF1	
GPR84		LIMD2	ISG15	MAB21L3	
PLK2		LOC100038328	ITGA7	MAN1C1	
NUCB1		LOC100125542	JADE2	MMP3	
FAM46A		LOC100513601	LOC102159510	PRADC1	
ISG15		LOC100738425	LOC106510200	PYGO1	
MCM2		LOC110258215	LOC110258215	ROGDI	
LOC595122		LOC110258822	LOC595122	RRBP1	
IER5		LOC110258825	MRM1	SASH3	
DDIT4		LOC110261410	MT-2B	SH3GL3	
ARHGEF12		MITF	MX1	SNX32	
ARHGAP6		MX1	NDUFAB1	STARD7	
SLC25A21		OASL	NINJ1	SVIP	
TPM4		PARP12	NRP2	TFR2	
MYL9		PARVA	NT5DC1	TICRR	
RHOU		PDK4	OASL	TLX1	
KRT18		PERP	PAQR4	TMPRSS2	

Overlapping (Jaing et al., 2017)	Overlapping (Yang et al., 2021)		Overlapping (Hualin Sun., 2021)
KRT8	PITPNM2	PARP12	UBE2J1
FCN1	PLSCR3	PFKFB3	WT1
GAS7	RASA1	PHKA2	
	RIPOR2	PKD2	
	RND1	PSD3	
	S100A2	RAB4A	
	SLA2	RNF144B	
	SLC16A7	RNF152	
	SLC38A1	S100A2	
	STK38L	SEMA4A	
	TIAM2	SERPINC1	
	TNF	SLC44A3	
	UBASH3B	SLC7A8	
	WDR31	TPCN1	
	ZPLD1	WNT9A	
		ZCWPW1	
